# Interactive effects of body mass changes and species‐specific morphology on flight behavior of chick‐rearing Antarctic fulmarine petrels under diurnal wind patterns

**DOI:** 10.1002/ece3.7501

**Published:** 2021-04-06

**Authors:** Nina Dehnhard, Andrew R. Klekociuk, Louise Emmerson

**Affiliations:** ^1^ Department of Biology Behavioural Ecology and Ecophysiology Group University of Antwerp Antwerp Belgium; ^2^ Department of Agriculture, Water and the Environment Australian Antarctic Division Kingston Tas. Australia; ^3^ Norwegian Institute for Nature Research (NINA) Trondheim Norway

**Keywords:** climate change, flight cost, foraging, gust soaring, katabatic wind, movement ecology, optimization, procellariiform

## Abstract

For procellariiform seabirds, wind and morphology are crucial determinants of flight costs and flight speeds. During chick‐rearing, parental seabirds commute frequently to provision their chicks, and their body mass typically changes between outbound and return legs. In Antarctica, the characteristic diurnal katabatic winds, which blow stronger in the mornings, form a natural experimental setup to investigate flight behaviors of commuting seabirds in response to wind conditions. We GPS‐tracked three closely related species of sympatrically breeding Antarctic fulmarine petrels, which differ in wing loading and aspect ratio, and investigated their flight behavior in response to wind and changes in body mass. Such information is critical for understanding how species may respond to climate change. All three species reached higher ground speeds (i.e., the speed over ground) under stronger tailwinds, especially on return legs from foraging. Ground speeds decreased under stronger headwinds. Antarctic petrels (*Thalassoica antarctica*; intermediate body mass, highest wing loading, and aspect ratio) responded stronger to changes in wind speed and direction than cape petrels (*Daption capense*; lowest body mass, wing loading, and aspect ratio) or southern fulmars (*Fulmarus glacialoides*; highest body mass, intermediate wing loading, and aspect ratio). Birds did not adjust their flight direction in relation to wind direction nor the maximum distance from their nests when encountering headwinds on outbound commutes. However, birds appeared to adjust the timing of commutes to benefit from strong katabatic winds as tailwinds on outbound legs and avoid strong katabatic winds as headwinds on return legs. Despite these adaptations to the predictable diurnal wind conditions, birds frequently encountered unfavorably strong headwinds, possibly as a result of weather systems disrupting the katabatics. How the predicted decrease in Antarctic near‐coastal wind speeds over the remainder of the century will affect flight costs and breeding success and ultimately population trajectories remains to be seen.

## Graphical abstract

Procellariiform seabirds such as petrels and albatrosses strongly depend on wind for their energy‐efficient flight behavior. We investigated the flight behavior of three closely related species of Antarctic fulmarine petrels, which differ in wing loading and aspect ratio. The diurnal coastal wind patterns and the fact that adult chick‐rearing birds show distinct body mass changes between outbound and return legs of foraging trips form a natural experiment which we used to test multiple hypothesis on how the petrels would adapt their flight speed, flight direction, and foraging trip distance under various headwind, tailwind, and crosswind conditions.

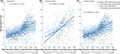

## 1 INTRODUCTION

Wind is a key feature of the environment that affects the flight costs of birds moving across their landscape to access their foraging grounds and breeding sites (e.g., Safi et al., 2013; Shepard et al., 2013). Flight styles, wing shape, and body mass (which determine the wing loading) are key characteristics that determine flight costs under different wind speeds and wind directions (Pennycuick, 2008). Procellariiform seabirds are particularly well adapted to utilize winds for energy‐efficient gust soaring (Pennycuick, 1982; Spear & Ainley, 1997a, 1997b). This is reflected by their global distribution and biodiversity patterns, which peak in the windiest parts of the Southern Ocean (Davies et al., 2010; Suryan et al., 2008).

Depending on specific wing shape, flying style, and wing loading, different seabird species have different energetic costs associated with foraging considerable distances away from their colonies or undertaking substantial migrations during the nonbreeding period (e.g., Elliott et al., 2013; Pennycuick, 2008). Within seabirds, albatrosses are well adapted to gust soaring, which enables them to fly for hours without flapping their wings (Richardson, 2011; Sachs et al., 2012), whereas most smaller procellariiforms combine gust soaring with occasional wing flapping (Gibb et al., 2017; Spear & Ainley, 1997b). The required wind speed for gust soaring is species‐specific and depends on the wing loading and thus the total wing area and body mass of the bird (Pennycuick, 2008; Sachs, 2005). If wind speeds are sufficiently high, procellariiforms can fly against the wind without flapping their wings, typically following a more tortuous track at lower average ground speed (i.e., speed of the bird flying over ground) than under cross‐ or tailwinds (Sachs et al., 2011). Nevertheless, flying against the wind, and thus under increased air speeds (i.e., speed of the bird relative to wind speed; at constant ground speed, air speed increases with head‐ and decreases with tailwind; Richardson et al., 2018), causes lower ground speeds (Wakefield et al., 2009). In addition, flying against the wind also increases the heart rate in wandering albatrosses (*Diomedea exulans*) and is thus less efficient than flying with cross‐ or tailwinds at higher ground speeds and therefore lower energy expenditure (Weimerskirch et al., 2000). Similarly, Manx shearwaters (*Puffinus puffinus*) have been shown to be more likely to fly energy‐efficiently by soaring under tailwinds and crosswinds, but less so under headwinds (Gibb et al., 2017), and Desertas petrels (*Pterodroma deserta*) have been shown to maximize ground speeds under quartering tailwinds (i.e., winds angled from the back; Ventura et al., 2020).

Favorable wind conditions are important for seabirds during the breeding season, and especially during chick‐rearing, when adults regularly commute between foraging areas and breeding colonies (Elliott & Gaston, 2005). This is illustrated by stronger wind speeds enabling shorter foraging trips and increased breeding success of wandering albatrosses at the Crozet Islands (Weimerskirch et al., 2012). Most studies that investigated the interplay between wind and flight behavior in seabirds focused on albatrosses, the largest gust‐soaring species with the highest wing loading. How winds affect the flight behavior of smaller procellariiforms such as petrels and shearwaters has been the focus of only few studies, most of which were based on visual observations (Spear & Ainley, 1997a, 1997b; but see Tarroux et al., 2016; Gibb et al., 2017). Better knowledge and understanding across more species and regions as to how seabirds make use of winds and the resultant energetic impacts from this is necessary given the dramatic changes expected for global wind patterns (IPCC, 2019), which may be beneficial for some species (Weimerskirch et al., 2012) but not others (Hass et al., 2012). This is becoming increasingly important since petrels and shearwaters are among the most threatened groups of birds in the world (Dias et al., 2019).

**FIGURE 1:**
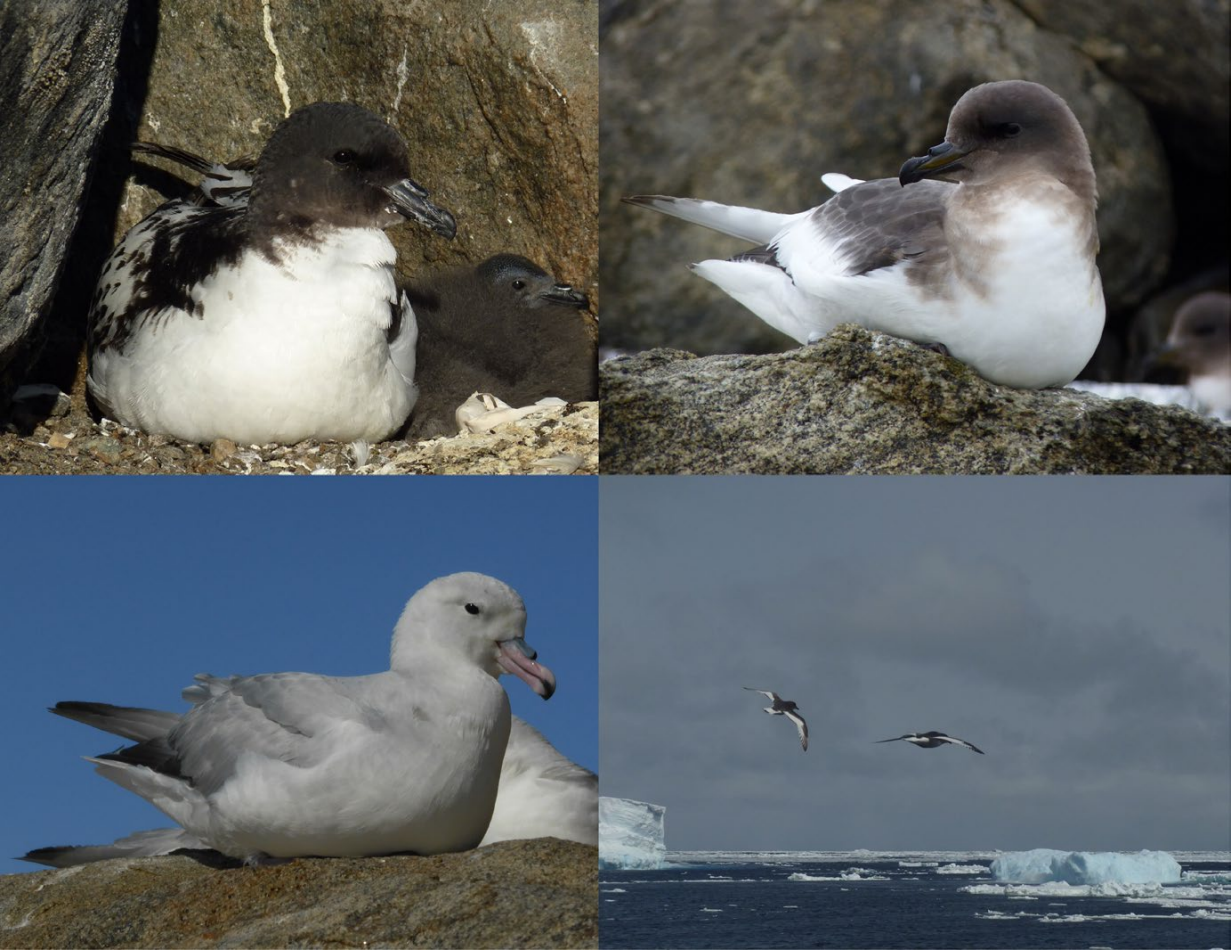
The three study species, cape petrel (top left), Antarctic petrel (top right), and southern fulmar (bottom left). The picture on the bottom right shows two Antarctic petrels gust soaring in the sea‐ice zone

The aim of this study was to investigate the flight behavior of three sympatrically breeding Antarctic fulmarine petrels in relation to local wind patterns. Cape petrels (*Daption capense*), Antarctic petrels (*Thalassoica antarctica*) and southern fulmars (*Fulmarus glacialoides*) are closely related and belong to the family Procellariidae (Figure 1). They are characterized by flap‐gliding flight (Spear & Ainley, 1997b), and reflect a gradient in average body mass, wing loading (i.e., body mass divided by total wing area), and aspect ratio (the ratio between squared wingspan and total wing area, as a descriptor of wing shape) (Table 1). Aspect ratio and wing loading have been shown to be correlated in fulmarine petrels, and the importance of both of these morphological traits on the flight behavior of these species has been documented in previous observational studies (Spear & Ainley, 1997a, 1997b).

**TABLE 1:** Average (±*SD*) body mass, wingspan, wing area, and resulting aspect ratio and wing loading for cape petrels, Antarctic petrels and southern fulmars at Hop Island, Antarctica

	Body mass in g	Wingspan in cm	Wing area in cm^2^	Wing loading in kg/m^2^	Aspect ratio	*N*
Cape petrel	469 ± 48	93 ± 3	762 ± 80	6.24 ± 0.99	11.31 ± 0.88	15
Antarctic petrel	714 ± 71	106 ± 4	957 ± 79	7.51 ± 0.95	11.86 ± 0.69	31
Southern fulmar	783 ± 85	116 ± 4	1,173 ± 91	6.65 ± 0.94	11.61 ± 0.76	25

Note

All birds were measured and weighed during the breeding season.

Coastal wind conditions in Antarctica are characterized by katabatic winds caused by cold air masses flowing down from the Antarctic plateau and moving seawards, which interact with the easterly drift of weather systems south of the Antarctic Divergence (Parish & Cassano, 2003). During the summer months, katabatic winds often show diurnal patterns, blowing stronger in the early morning hours (Parish & Cassano, 2003; Turner et al., 2009). This enables an investigation of strategies of seabirds as to whether they avoid unfavorable wind conditions when commuting to and from their foraging areas or alternatively, whether they are able to take advantage of particular wind conditions. A recent study found high overlap in the timing of foraging and space use of the three species during chick rearing, during which all three species foraged relatively close to their colony (maximum distance from nest <500 km; Dehnhard et al., 2020). While wind patterns in this area show a diurnal pattern, light levels during the austral summer, when these species are rearing their chicks, allow foraging over at least 20 hr each day (Dehnhard et al., 2020).

In detail, we aimed to test the following predictions:

1. Based on previous observational data on procellariiform seabirds including our study species (Spear & Ainley, 1997b), we predicted that birds will have higher ground speeds with higher wind speeds under tailwinds but not under cross‐ or headwinds.
2. Between species, we expected morphology and particularly wing loading and/or aspect ratio to affect average ground speeds and air speeds. Since aspect ratio and wing loading in fulmarine petrels are correlated (Spear & Ainley, 1997a) and both show a similar gradient in our three study species, identifying which of the two factors is responsible is, however, not possible in the framework of this study. Species with higher wing loading require higher air speeds and thus also higher wind speeds in order to gust soar (Pennycuick, 2008), but can then be expected to reach higher ground speeds under higher wind speeds (cf. Wakefield et al., 2009). We therefore expected species to differ in their response to increasing wind speeds resulting in different relationships between the birds’ ground speed and wind speed for each species. Based on the differences in wing loading and aspect ratio, under tailwinds we expected Antarctic petrels to reach higher ground speeds under higher wind speeds, followed by southern fulmars and last cape petrels. We expected this response to be reversed or possibly absent under headwinds and crosswinds based on previous observational studies (Spear & Ainley, 1997b).
3. Within species, we expected a differential response of ground speed in relation to wind speed between outbound and return commutes, since parental birds should return with a meal for their chicks, and thus, body mass and wing loading should be higher on return than on outbound legs. We thus predicted that under tailwinds, birds would show a steeper increase in ground speed in response to wind speed on return legs compared with outbound legs, but to show an absence of this relationship or possibly the opposite pattern under cross‐ and headwinds based on previous observational studies (Spear & Ainley, 1997b).
4. Based on prediction (1) and albatrosses behavior (Weimerskirch et al., 2000), we expected our study species to favor tailwinds and possibly crosswinds but avoid headwinds on their outbound trip to foraging grounds and their return commutes to the colony. We therefore expected (4.1) birds to adjust their flight direction in relation to wind direction to avoid unfavorable strong headwinds and crosswinds on both outbound and return legs and/or (4.2) that birds would adjust the timing of their outbound and return commutes in response to any diurnal wind pattern to allow them to avoid unfavorable winds. Finally (4.3), we expected birds to limit their maximum distance traveled from their nest when encountering headwinds on outbound legs.

In a final step, we compared wind speed and wind direction data from our study period and the area utilized by the birds with historic data and simulated data from climate change scenarios for the future, to assess whether climate change may in the long term affect our study populations.

## 2 MATERIALS AND METHODS

### 2.1 Fieldwork

Fieldwork was conducted in the Rauer Island group near Davis Research Station in the Prydz Bay region, East Antarctica, during the austral summer 2015/16. We tracked breeding cape petrels, Antarctic petrels, and southern fulmars from two mixed colonies located in the northwest of Hop Island within 2 km of each other (68.819°S, 77.689°E and 68.821°S, 77.678°E, respectively).

We used Sterna and Pica GPS loggers from Ecotone Telemetry (Gdynia, Poland), fitted with solar panels and a remote download function as detailed in Dehnhard et al. (2020). Loggers were attached to the back feathers using a combination of Tesa® tape (Beiersdorf) and warmed mastic (3M) and Loctite 401 (Henkel) to seal off tape ends. During deployments, birds were weighed (to the nearest 5 g, using spring scales), and we measured ½ wingspan (using a tape measure from the backbone to the wingtip, to the nearest 0.5 cm). We drew the outline of one wing per bird on a paper to determine average wing area per species and calculate wing loading and aspect ratio as described in Pennycuick (2008). The weight of the loggers with tape and glue was 6–8 g, and thus in the range of 1.0 to 1.7% of the birds’ average body mass (see Table 1), and below 2% of the lightest bird's body mass (lightest cape petrel weighed in this study: 410 g). Most deployments were during the incubation stage, and some additional loggers were deployed during chick‐rearing (see Dehnhard et al., 2020). The intention was to leave loggers on during the entire breeding season. Few loggers were recovered at the end of the breeding season, but most birds were only captured once (during deployment), and either preened off their loggers or lost these during molt. Here, we only included complete tracks from chick‐rearing, that is, 21 tracks of 8 Antarctic petrels, 79 trips of 8 cape petrels, and 92 trips of 10 southern fulmars, tracked between the 11 January and the 12 March.

### 2.2 Treatment of data

GPS loggers were programmed to record GPS positions at 15‐min intervals, and wet‐dry data (dive in/dive out) every second. We interpolated positions when minor data gaps were present using great circle distances of each bird to regular 15‐min intervals. Ground speed was calculated based on the great circle distance between two subsequent GPS fixes, and flight direction of the birds was calculated between the same two GPS fixes. We defined foraging trips to be those that exceeded a distance of 10 km from the nest and contained dive data. Trips were divided into outbound, middle, and return legs, following the methodology of Wakefield et al. (2009). Briefly, thresholds for outbound, middle, and return legs of foraging trips were determined on the population level based on the maximum distance reached and the proportion of the total trip time. Since the focus of our study was on the commuting part, we focused on the outbound and return legs and excluded middle sections and any periods when birds were foraging or resting and not commuting. To do so, we identified foraging locations based on the occurrence of dive events (originally recorded every second as dive in or dive out event), which were aggregated over each 15‐min GPS interval. This resulted in a binary variable which we used as indicator of foraging activity (0 = no foraging activity [no dive event]; 1 = foraging activity [one or more dives within 15‐min interval]). Since diving bouts were often followed by resting periods, during which birds were comparatively stationary (see Dehnhard et al., 2020), we further applied expectation–maximization binary clustering (EMbC; Garriga et al., 2016b). EMbC uses velocity and turning angle to classify movement data into four different clusters aligned with likely behavioral states: low velocities and low turns (LL, which could be interpreted as resting behavior), low velocities and high turns (LH, intensive search), high velocities and low turns (HL, traveling or relocation), and high velocities and high turns (HH, extensive search) (Garriga et al., 2016b). We analyzed our GPS dataset in the EMbC R package (Garriga et al., 2016a) as detailed in Dehnhard et al. (2020). Wet data (i.e., apparent diving activity) coincided mostly with EMbC states LL and LH, less frequently with EMbC state HH, and least with EMbC state HL (Appendix S2 in Dehnhard et al., 2020).

For the subsequent analyses of wind speed and wind direction on commuting legs, we only took paths of the GPS tracks into account that were not associated with diving (i.e., foraging activity = 0) and annotated as EMbC state HL (high speed, low turning angle = commuting) or EMbC state HH (high speed, high turning angle = extensive foraging, but possibly also tortuous flight under headwind conditions). Time stamps with EMbC states LL and LH (low speed and low or high turning angle, respectively) were excluded.

We further removed positions within 2 km of the colony to exclude the potential impact of interactions with other birds, land structures and cliffs on local wind patterns, and thus flight behavior near the colony.

Despite this “data cleaning,” some data points with low ground speeds remained (129 records for ground speeds of <0.5 m/s, within a dataset of 6,356 data points in total). We cannot be 100% certain that birds in these instances were in fact commuting. In few cases, the low ground speeds may have been due to birds tacking against unfavorably strong headwinds or using active flapping flight. While this is unlikely to explain all cases, we decided against excluding data points with a ground speed below a certain—arbitrarily set—threshold. We assume that the size of our dataset is sufficiently large to yield robust results with the inclusion of such potential outliers.

We extracted the times for sunrise, sunset, nautical dusk, and nautical dawn (when the sun is 12° below the horizon) for each of the birds’ GPS positions in the R‐package *maptools* (Bivand & Lewin‐Koh, 2016) to determine light levels experienced by the birds during their foraging trips. Time of the day is given as local time.

Wind speed and direction at 10 m height was extracted from gridded forecast data (Antarctic Mesoscale Prediction System (AMPS) Polar Weather and Research Forecasting (Polar WRF) model version 3.7.1 (Bromwich et al., 2013) with 3 hr by 10 km horizontal resolution; http://www2.mmm.ucar.edu/rt/amps/wrf_grib/) and matched in time and space to the GPS position data of the birds using *raadtools* (Sumner, 2017). Polar WRF provides higher resolution than current meteorological reanalyses and performs adequately in evaluating surface wind in the Antarctic (Bromwich et al., 2013). As in Tarroux et al. (2016), we used forecast data 12 hr after each analysis to allow the model to adequately equilibrate with the analysis cycle.

We calculated the absolute difference between the birds’ flight direction and wind direction (hereafter ΔDir_fw_), which was on a scale from 0° to 180°. Since wind direction is defined as the direction from which the wind is coming, while flight direction is the direction into which the bird is flying, ΔDir_fw_ is at 90° if a bird is flying perpendicular to the wind (i.e., crosswind), decreasing if the bird is flying against the wind (with maximum headwind at 0°), and increasing if a bird is flying with the wind (maximum tailwind at 180°). To compare wind conditions that the birds experienced at sea on their foraging trips with those near their breeding colony, we obtained hourly wind speed and wind direction data from the two nearest weather stations, that is, Davis Research Station (68.577° S, 77.968° E; 30 km north‐northeast of Hop Island) and Zhong Shan Station (69.374° S, 76.372° E; 80 km south‐southwest of Hop Island).

### 2.3 Statistics

All statistical procedures were run in R version 3.6.1 (R Core Team, 2020). Linear mixed models (LMMs) to test predictions 1–3 and 4.3 and generalized linear mixed models (GLMMs) to test prediction 4.1 were run in the R‐package *lme4* (Bates et al., 2011). P‐values for LMMs were computed in *lmerTest* (Kuznetsova et al., 2014), and for GLMMs by using the *ANOVA* function in R to compare the model with and without the variable of interest. Interaction terms were illustrated using the R‐package *interactions* (Long, 2019). Where appropriate, post hoc tests based on pairwise comparisons of least square means (LSM) were performed in the *emmeans* package (version 2.30‐0; Lenth, 2016) using Tukey's method for p‐value adjustment. We present marginal *R* ^2^ values (*R* ^2^_m_, for the variance explained only by fixed effects) and conditional *R* ^2^ values (*R* ^2^_c_, based on the variance explained by both fixed and random effects), calculated in R‐package *sjstats* (Lüdecke, 2021).

Generalized additive mixed models (GAMMs) to test prediction 4.2 were run in the R‐package *mgcv* (version 1.8‐3.1; Wood, 2016). Model assumptions for LMMs, GLMMs, and GAMMs were validated using the protocols described in Zuur et al. (2009) and Wood (2017). Significance level was *p* = .05.

To test predictions 1–3 and thus the influence of wind speed, wind direction, species, and trip section on ground speed of birds, we set up a global LMM with ground speed as the dependent variable and wind speed (continuous), ΔDir_fw_ (continuous), species (factor), and trip section (two‐level factor; outbound or return leg), as well as all possible 2‐way, 3‐way, and the 4‐way interactions, as explanatory variables. Trip nested within BirdID was included as random factor in the LMM. We attempted a backward stepwise model selection, by simplifying the model structure (Burnham & Anderson, 2002). However, the 4‐way interaction term between wind speed, ΔDir_fw_, species, and trip section was significant (see Results), and thus, interpretation of main effects in this main model was not straightforward. We therefore proceeded by splitting the dataset in subsets, by either species or wind direction (transforming wind direction into a categorical variable; see below) to test our detailed predictions. The dependent variable remained ground speed in all these models, and the random factor remained trip nested within BirdID, while the number and combination of explanatory variables differed depending on the predictions, as follows.

To test prediction 1, that is, whether ground speeds are affected by a combination of wind speed and wind direction relative to flight direction, we included wind speed (continuous), ΔDir_fw_ (continuous), and species (factor), as well as all possible 2‐way and 3‐way interactions as explanatory variables. The 3‐way interaction term between wind speed, ΔDir_fw_, and species was significant, indicating a different response between the three species (Section 3). To interpret differences in the response between species, we split the dataset by species and ran simplified LMMs with wind speed and ΔDir_fw_, as well as the two‐way interaction.

To test prediction 2, that is, whether the effect of wind speeds on ground speeds under tailwinds, crosswinds, and headwinds differ between species, we split the dataset by ΔDir_fw_, thereby transforming ΔDir_fw_ into three categories, with 0° ≥ ΔDir_fw_ ≤ 60° being headwind, 60° ≥ ΔDir_fw_ ≤ 120° being crosswind, and 120° ≥ ΔDir_fw_ ≤ 180° being tailwind (hereafter wind categories). LMMs were then run separately for headwind, crosswind, and tailwind and included wind speed (continuous), species (factor), and all possible 2‐way interactions as explanatory variables. To interpret the main effects, we split the dataset further by species in the case of a significant two‐way interaction or—in the case of nonsignificant interaction terms—we simplified the model by removing the interaction terms.

Prediction 3 aimed to identify whether wind affected ground speed differently on outbound versus return trip sections. Since we found a significant 4‐way interaction between wind speed, ΔDir_fw_, species, and trip section for the global model, as well as significant effects of species, wind speed, and wind categories on ground speed when testing predictions 1 and 2 (see Section 3), we set up models separately per species and wind category. Our models therefore contained wind speed (continuous), trip section (factor), and the two‐way interaction. As for prediction 2, we simplified models in the case of nonsignificant interaction terms.

To test prediction 4.1, we investigated whether birds adjusted their flight direction in response to wind direction and wind speed and tested for differences between species and trip sections. We thus ran a GLMM with ΔDir_fw_ as dependent variable, species, trip section, and wind speed as explanatory variables, as well as all possible 2‐ and 3‐way interaction terms. ΔDir_fw_ was rescaled between 0 and 1 and a binomial error distribution was used in the model to account for the fact that ΔDir_fw_ is restrained between 0 and 180°. As with the LMMs, we included trip nested within BirdID as random factors and subsequently simplified the model by removing nonsignificant interaction terms and/or continued the analyses by splitting the dataset by species and trip section. To test prediction 4.2, we investigated whether outbound and return sections of foraging trips were uniformly distributed over the course of the day and whether birds encountered headwinds, crosswinds, and tailwinds uniformly over the day. We used GAMMs to test these relationships since GAMMs allow the fitting of nonlinear responses to predictor variables, and we expected a nonlinear distribution of commuting activity over the course of the day. We thus calculated the proportion of locations per hour (separately for each bird) for either the trip section (outbound or return legs) or the three different wind categories (headwind, tailwind, and crosswind). Proportions were calculated per BirdID instead of trip, since at maximum 4 locations per hour could belong to the same trip (loggers were programmed to collect GPS data at 15‐min intervals), and the amount of variation between trips of the same individual was therefore low.

We thus ran two different sets of GAMMs: one with the proportion of outbound and return trip sections as the dependent variable, and one with the proportion of wind category encountered on the commute as the dependent variable. Species, and trip section (for the first set of GAMMs) or wind category (for the second set of GAMMs) were included as explanatory variables into the global GAMM, together with all possible 2‐way interactions. We further included hour of the day (with a tensor product smoother accounting for circularity) and also the interaction terms between hour of day and species, as well as hour of day and trip section or hour of day and wind category, respectively (as tensor product interactions). BirdID was included as a random effect. We initially set the maximum number of knots to 5 in order to avoid overfitting, and used the function gam.check to check whether models with more knots had a better fit. GAMMs were run on a binomial distribution (since the dependent variable was a proportion). We subsequently attempted to simplify the models and remove nonsignificant terms, starting with interaction terms.

Finally, to test prediction 4.3, that is, whether birds would limit their maximum distance from their nest when encountering headwinds on outbound legs, we investigated whether maximum distance from nest was affected by the average difference between the wind direction and the birds’ flight direction (ΔDir_fw_) across the outbound trip. Our dataset therefore consisted of only one data point per trip. We used LMMs with maximum distance from nest as dependent variable, species, and average ΔDir_fw_ on the outbound trip section as explanatory variables, together with the 2‐way interaction term. BirdID was included as random factor.

### 2.4 Past and future wind conditions in the study area

Monthly mean surface wind speeds for January to March in the region of the study site were obtained for the period 1979–2019 using the ERA5 reanalysis (Hersbach et al., 2020). Linear trends over this time period were analyzed separately per month using linear regressions.

To obtain more specific information for our region of interest and time of year specifically for the future, we examined simulations from the 6th Coupled Model Intercomparison Project (CMIP6; Eyring et al., 2016). CMIP6 model data were obtained from the Earth System Grid Federation (https://esgf.llnl.gov/). We selected one ensemble member from models having generally different heritage. The CMIP6 models used were ACCESS‐ESM1‐5, AWI‐CM‐1‐1‐MR, BCC‐CSM2‐MR, CanESM5, GFDL‐ESM4, IPSL‐CM6A‐LR, MIROC6, and MRI‐ESM2‐0. All models accurately reproduced the strength of the meridional surface wind speed in comparison with observational data provided by the ERA5 reanalysis. However, there was less agreement with the speed of the zonal wind, with differences of up to approximately ± 3 m/s across models compared with ERA5. Over the historical period, the interannual variability of the zonal wind was well captured in the models, although the variability in the meridional component was larger than in ERA5.

## 3 RESULTS

### 3.1 Wind conditions at the coast and encountered on foraging trips

**FIGURE 2:**
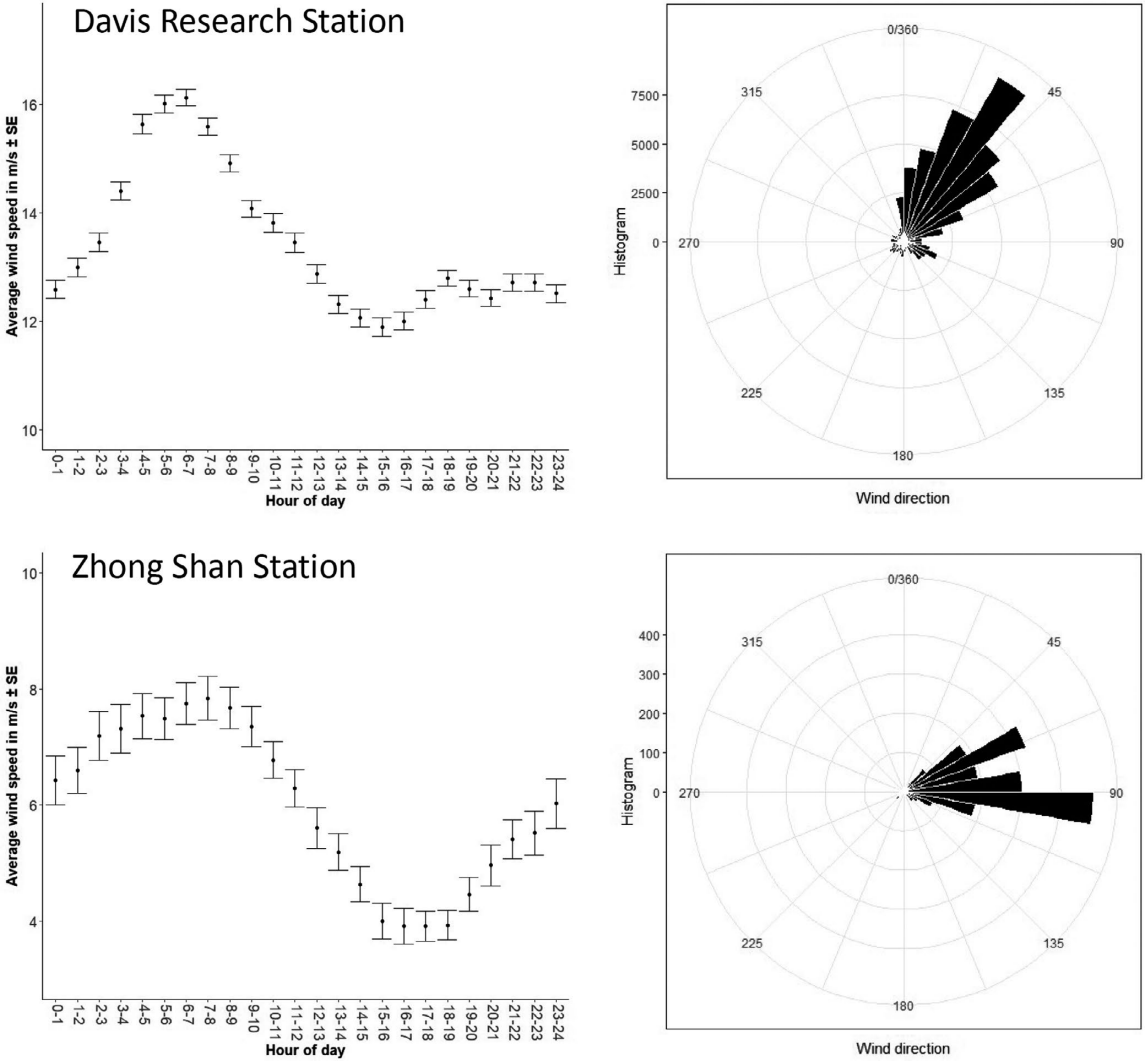
Plot showing wind speed over the course of the day (left) and the predominating wind direction (right) at Davis Research station (located 30 km north‐northeast of Hop Island) and Zhong Shan station (located 80 km south‐southwest from Hop Island) during the study period (11 January to 11 March 2016)

**FIGURE 3:**
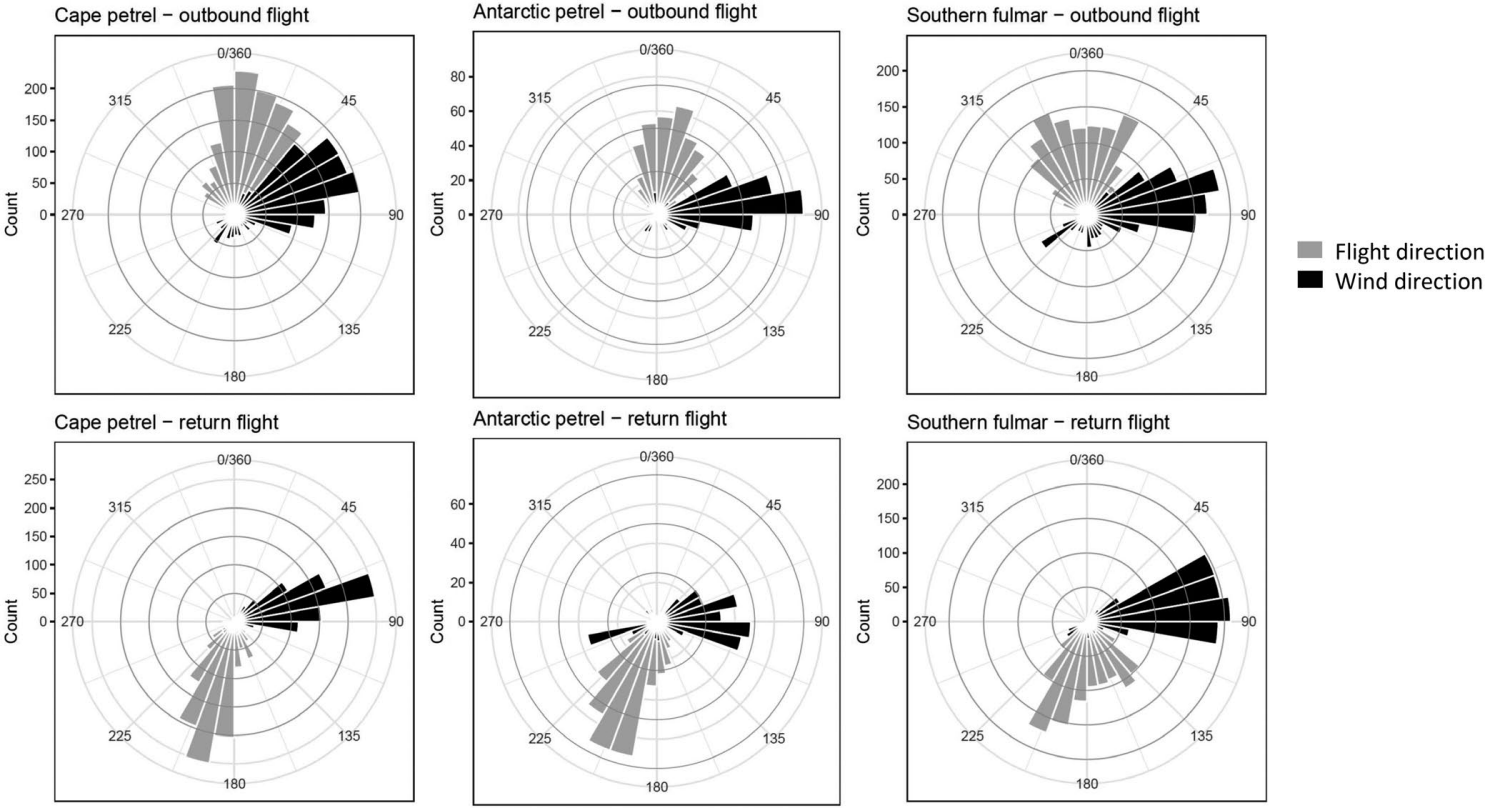
Circular histograms of flight direction of Antarctic petrels, cape petrels, and southern fulmars and experienced wind direction on outbound and return sections of foraging trips. Note that flight direction is the direction into which the bird is flying (i.e., flight direction of 180° means the bird flies southwards), while wind direction is the direction from which the wind is blowing (i.e., wind direction of 90° means the wind is blowing from the east)

Predominant wind direction in the coastal area around the breeding site was from the northeast to east. Wind speeds peaked in the early morning and were lowest at midday (Figure 2). On their commute to foraging areas, birds experienced mostly easterly winds (Figure 3). During outbound legs, birds of all three species headed into northwesterly to northeasterly directions, while flight directions were south to southwest during return legs (Figure 3, Appendix S1). The majority of foraging trips described a loop in clockwise direction, in which case birds flew eastwards during the middle section (Appendix S2). This pattern was more distinct for Antarctic petrels (19 out of 21 foraging trips) than for the other two species (50 out of 79 foraging trips of cape petrels and 58 out of 91 foraging trips of southern fulmars, respectively).

### 3.2 Ground speed in relation to wind speed, ΔDir_fw_, species, and trip section

Ground speed was significantly affected by the 4‐way interaction between wind speed, wind direction relative to flight direction (ΔDir_fw_), species, and trip section (Table 2, Model m_full).

**TABLE 2:** Outcomes of linear mixed models (LMMs) investigating the effects of wind speed, wind direction relative to flight direction (ΔDir_fw_), species, and trip section (outbound or return commute) on ground speed (as dependent variable in all models). Starting with the initial full model (m_full), models were reduced in complexity and the dataset split to investigate the effects in detail and test predictions 1, 2, and 3 (see Section 2). Significant effects are marked in bold, test statistics refer to the variables marked in red in the main model

Model name	Explanatory variables	Interaction terms	Data	Test statistic for variables marked in red
m_full	wind speed + ΔDir_fw_ + species + trip section	all possible 2‐way interactions + 3‐way interactions + wind speed*ΔDir_fw_*species*trip section	All	***F*_2_ = 11.58, *p* < .001**
Testing prediction 1				
m1	wind speed + ΔDir_fw_ + species	all possible 2‐way interactions + wind speed* wind speed*ΔDir_fw_*species	All	***F*_2_ = 50.77, *p* < .001**
m1.1	wind speed + ΔDir_fw_	wind speed*ΔDir_fw_	Cape petrel	***F*_1_ = 505.56, *p* < .001**
m1.2	wind speed + ΔDir_fw_	wind speed*ΔDir_fw_	Antarctic petrel	***F*_1_ = 75.44, *p* < .001**
m1.3	wind speed + ΔDir_fw_	wind speed*ΔDir_fw_	Southern Fulmar	***F*_1_ = 24.99, *p* < .001**
Testing prediction 2				
m2.1	wind speed + species	wind speed*species	Tailwind	***F*_2_ = 12.64, *p* < .001**
m2.1.1	**wind speed**		Tailwind Cape petrels	***F*_1_ = 151.17, *p* < .001**
m2.1.2	**wind speed**		Tailwind Antarctic petrels	***F*_1_ = 18.48, *p* < .001**
m2.1.3	**wind speed**		Tailwind Southern fulmars	***F*_1_ = 7.41, *p* = .007**
m2.2	wind speed + species	wind speed*species	Crosswind	*F*_2_ = 1.49, *p* = .225
m2.2_red	wind speed + **species**		Crosswind	*F*_1_ = 1.02, *p* = .312; ***F*_2_ = 3.94, *p* = .036**
m2.3	wind speed + species	wind speed*species	Headwind	***F*_2_ = 15.60, *p* < .001**
m2.3.1	**wind speed**		Headwind Cape petrels	***F*_1_ = 71.75, *p* < .001**
m2.3.2	**wind speed**		Headwind Antarctic petrels	***F*_1_ = 34.94, *p* < .001**
m2.3.3	**wind speed**		Headwind Southern fulmars	***F*_1_ = 26.10, *p* < .001**
Testing prediction 3				
m3.1.1	wind speed + trip section	wind speed*trip section	Tailwind Cape petrels	***F*_1_ = 18.74, *p* < .001**
m3.1.2	wind speed + trip section	wind speed*trip section	Tailwind Antarctic petrels	***F*_1_ = 8.95, *p* = .003**
m3.1.3	wind speed + trip section	wind speed*trip section	Tailwind Southern fulmars	***F*_1_ = 4.56, *p* = .033**
m3.2.1	wind speed + trip section	wind speed*trip section	Crosswind Cape petrels	***F*_1_ = 7.43, *p* = .006**
m3.2.2	wind speed + trip section	wind speed*trip section	Crosswind Antarctic petrels	*F*_1_ < 0.01, *p* = .975
m3.2_red	wind speed + **trip section**		Crosswind Antarctic petrels	*F*_1_ = 2.41, *p* = .122; ***F*_1_ = 5.67, *p* = .018**
m3.2.3	wind speed + trip section	wind speed*trip section	Crosswind Southern fulmars	***F*_1_ = 6.76, *p* = .009**
m3.3.1	wind speed + trip section	wind speed*trip section	Headwind Cape petrels	*F*_1_ = 0.64, *p* = .426
m3.3.1_red	**wind speed + trip section**		Headwind Cape petrels	***F*_1_ = 79.71, *p* < .001; *F*_1_ = 52.42, *p* < .001**
m3.3.2	wind speed + trip section	wind speed*trip section	Headwind Antarctic petrels	*F*_1_ = 0.13, *p* = .722
m3.3.2_red	**wind speed** + trip section		Headwind Antarctic petrels	***F*_1_ = 29.17, *p* < .001**; *F*_1_ = 2.73, *p* = .103
m3.3.3	wind speed + trip section	wind speed*trip section	Headwind Southern fulmars	*F*_1_ = 2.11, *p* = .147
m3.3.3_red	**wind speed + trip section**		Headwind Southern fulmars	***F*_1_ = 23.98, *p* < .001; *F*_1_ = 20.65, *p* < .001**

Note

*N* = positions for Antarctic petrels, cape petrels, and Southern fulmars, respectively.

**FIGURE 4:**
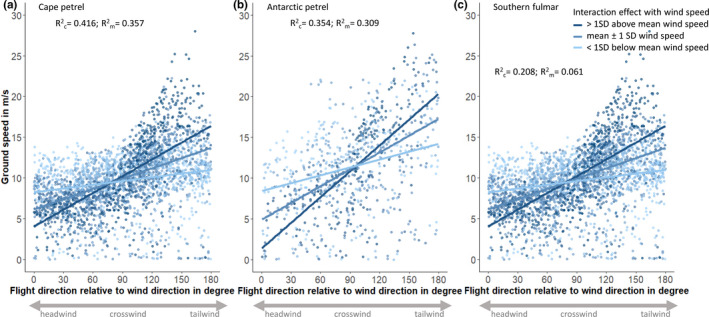
Birds’ ground speeds in response to the difference between the wind direction and the birds’ flight direction (ΔDir_fw_) for different wind speeds. Plots are based on models m1.1, m1.2, and m1.3 (Table 2). Solid lines reflect the significant interaction effects between wind speed and ΔDir_fw_ under mean ± 1 *SD* wind speeds, as well as wind speeds > 1 *SD* above and below the mean. *N* = 832 data points for Antarctic petrels, 2,972 for cape petrels, and 2,661 for southern fulmars, respectively

**FIGURE 5:**
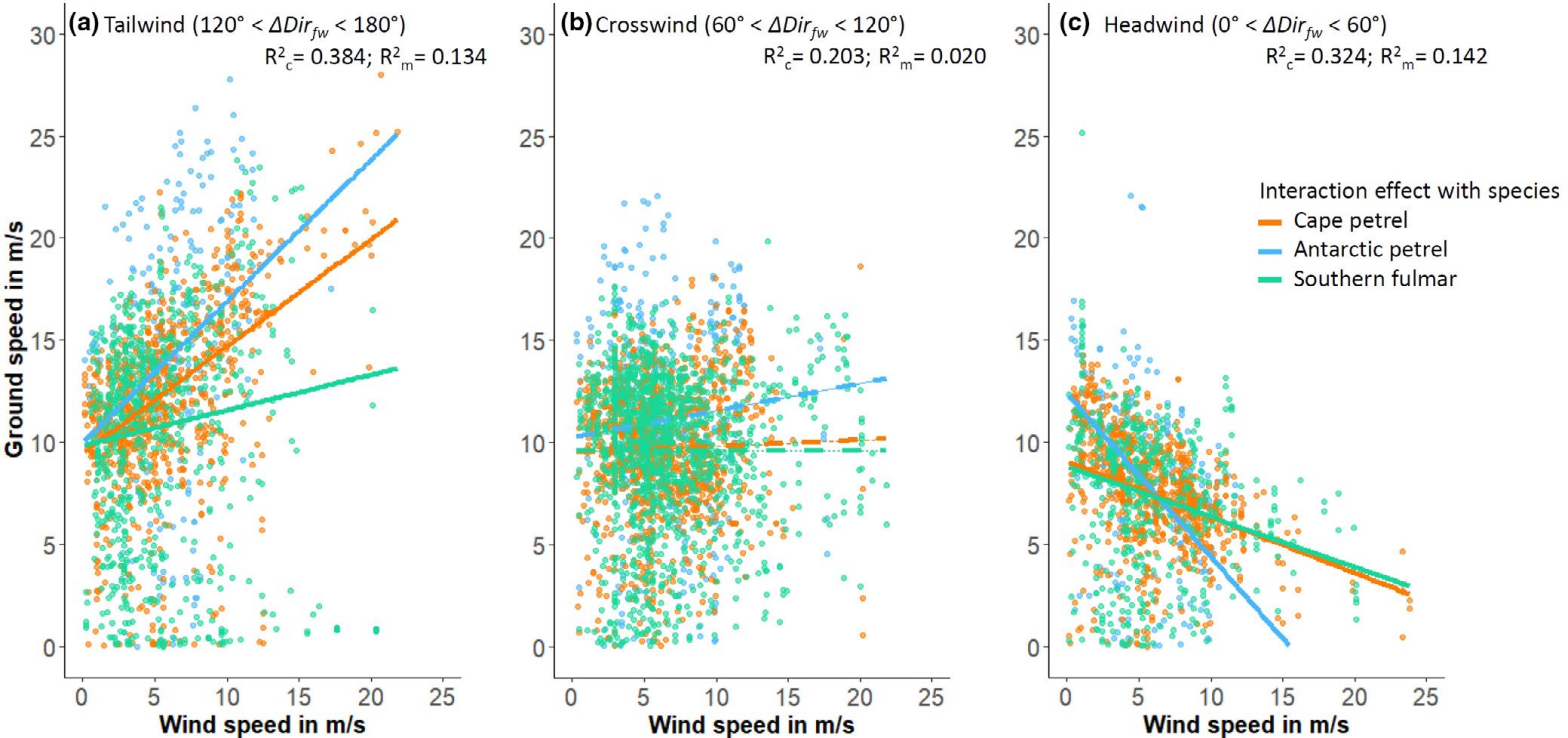
Species differences in ground speed in response to wind speed under tail‐, cross‐, and headwind in Antarctic petrels, cape petrels, and southern fulmars. Interaction effects between species and wind speed are illustrated with solid lines when significant, and dashed lines when nonsignificant. Plots are based on models m2.1, m2.2, and m2.3 (Table 2)

Testing prediction 1 (birds should have higher ground speeds with higher wind speeds under tailwinds but not under cross‐ or headwinds), the interaction between wind speed and ΔDir_fw_ was significant for all three species (Table 2, Models 1.1 to 1.3). Ground speed increased in all three species with increasing ΔDir_fw_ and thus an increasing tailwind component (Figure 4). A 45° change in ΔDir_fw_ toward more tailwind meant on average an increase by 3.15 m/s in ground speed for Antarctic petrels, 1.8 m/s for cape petrels, and 1.35 m/s for southern fulmars, respectively. In agreement with prediction 1, ground speed increased with increasing wind speed in all three species under tailwind, while the opposite was true for headwinds (Figures 4 and 5, Table 2; Models 2.1.1 to 2.1.3 and 2.3.1 to 2.3.3). As such, an increase in wind speed by 5 m/s under tailwind leads to an increase in ground speed of 3.45 m/s in Antarctic petrels, 2.6 m/s in Cape petrels, and 0.85 m/s in southern fulmars. In contrast, a 5 m/s increase in wind speed under headwinds caused a decrease in ground speed by 4 m/s in Antarctic petrels, 1.35 m/s in Cape petrels, and 1.2 m/s in southern fulmars, respectively. Also in agreement with prediction 1, wind speed had no significant effect on ground speed under crosswinds (Figure 5b, Table 2; Model 2.2_red).

In agreement with prediction 2 (morphology and particularly wing loading should affect average ground speeds of the three species), we found significant interaction terms between species and wind speed for both tailwind and headwind on the birds’ ground speed (Figure 5; Models 2.1 and 2.3), indicating a species‐specific response to different wind speeds. Under crosswinds, ground speed differed significantly between species, but the interaction between wind speed and species was not statistically significant (Figure 5b, Models 2.2 and 2.2_red). Antarctic petrels had higher average ground speeds than southern fulmars under tailwinds (LSM; *t* = 3.33, *p* = .005), and they visually showed a steeper increase in ground speed under increasing wind speeds than the other two species (Figure 5a), thus matching prediction 2. However, contrasting prediction 2, cape petrels (the species with the lowest wing loading) had intermediate ground speed levels and did not differ significantly from either southern fulmars or Antarctic petrels under tailwinds for average wind speeds (LSM; *t* ≤ |2.29|, *p* ≥ .089; Figure 5a). Also under headwinds, cape petrels and southern fulmars visually showed a very similar decrease in ground speed in response to increasing wind speeds, while Antarctic petrels showed—agreeing with prediction 2—the strongest response (Figure 5c).

**FIGURE 6:**
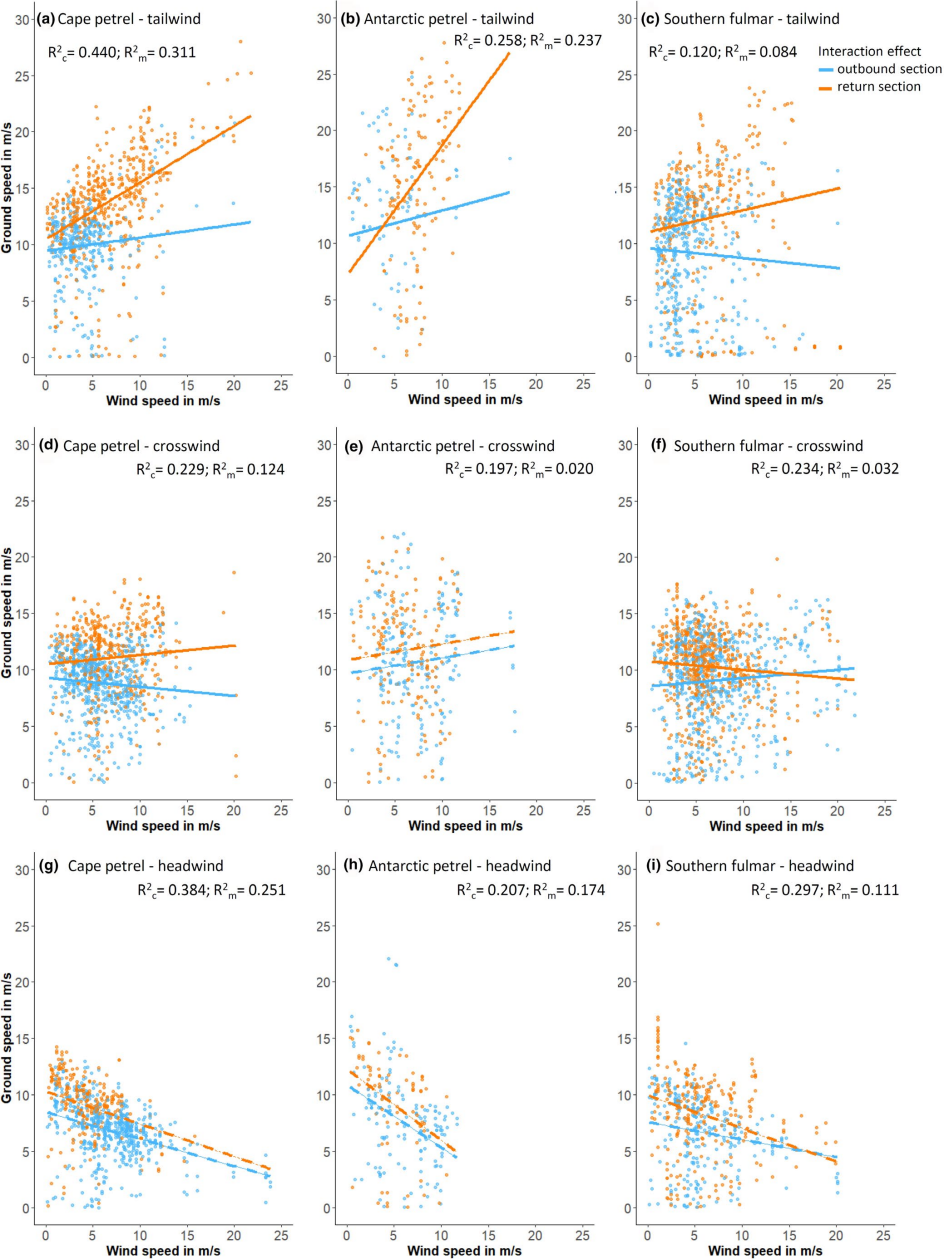
Ground speed in response to wind speed under tail‐, cross‐, and headwind and separately for outbound and return sections of foraging trips in Antarctic petrels, cape petrels, and southern fulmars. Plots are based on models m3.1.1‐m3.1.3, m3.2.1‐m3.2.3, and m3.3.1–3.3.3 (Table 2). Interaction effects between trip sections and wind speed are illustrated with solid lines when significant, and dashed lines when nonsignificant

Returning to the 3‐way interaction between wind speed, ΔDir_fw_, and trip section, the interaction between wind speed and trip section was significant for all three species under tailwinds (Table 2, Models 3.1.1 3.1.3). Matching prediction 3 (the response of ground speed in relation to wind speed should differ between outbound and return commutes), ground speed increased for all three species with a steeper slope for outbound than for return trip sections (Figure 6a–c). Under crosswinds, the interaction term between trip section and wind speed was significant only for cape petrels and southern fulmars but not Antarctic petrels (Models 3.2.1–3.2.3). The direction of the relationship was reversed between southern fulmars and cape petrels, while Antarctic petrels reached generally higher ground speeds on return than outbound trip sections (Figure 6d–f). Finally, under headwinds, interaction terms between trip section and wind speed were nonsignificant for all three species (Models 3.3.1–3.3.3; Figure 6g–i). Cape petrels and southern fulmars reached higher ground speeds on return compared with outbound legs, while there was no significant difference for Antarctic petrels (Models 3.3.1_red, 3.3.2_red and 3.3.3_red).

### 3.3 Flight direction, timing of commute, and maximum distance to colony in relation to wind conditions

**FIGURE 7:**
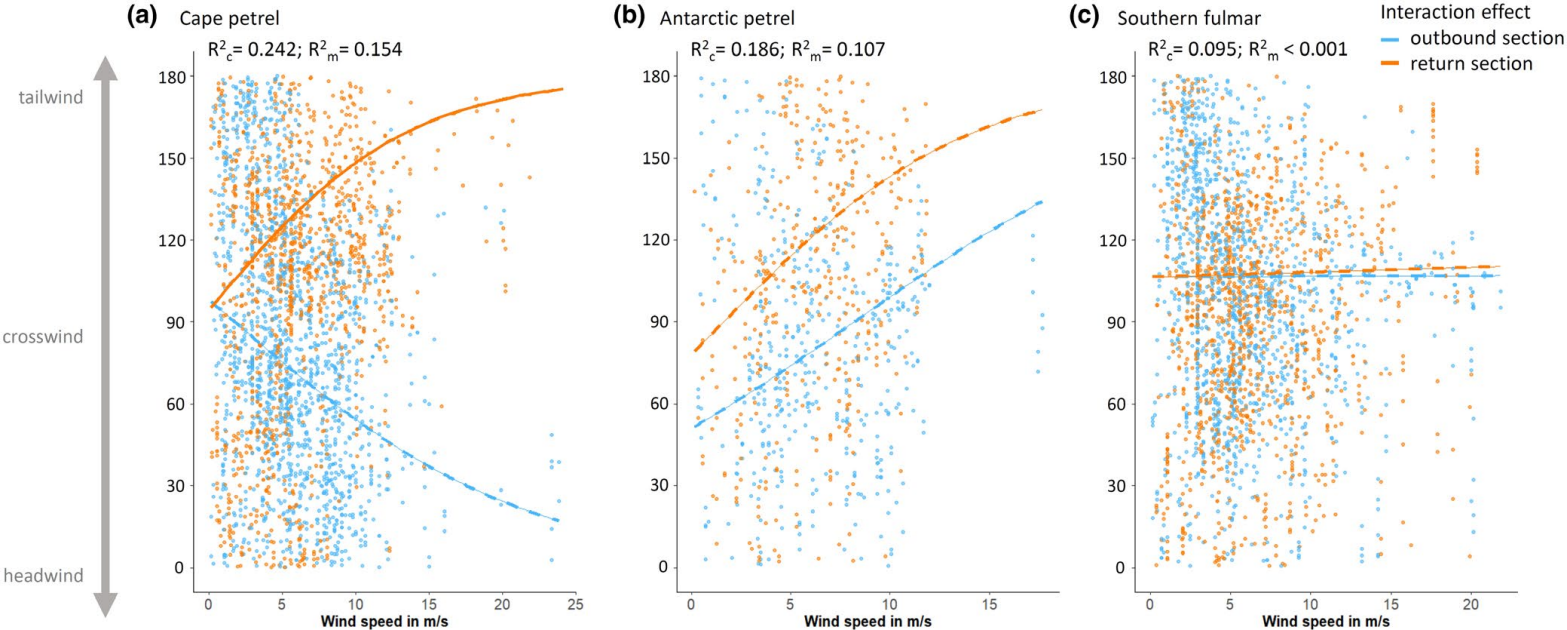
Flight direction relative to wind direction (ΔDir_fw_) for outbound and return journeys in response to wind speed under tail‐, cross‐, and headwinds. Plots are based on models m4.1.1, m4.1.2, and m4.1.3. Lines represent interaction effects between trip sections and wind speed. Solid lines represent a significant relationship between wind speed and ΔDir_fw_, and dashed lines, a nonsignificant relationship

Flight direction relative to wind direction (ΔDir_fw_) was significantly affected by the three‐way interaction between wind speed, species, and trip section (Table 3, Model m4.1_full). Cape petrels and Antarctic petrels experienced on average smaller angles between flight direction and wind direction (i.e., smaller ΔDir_fw_), and thus more headwinds, on their outbound compared with return legs (LSM; *z* > |5.60|, *p* < .001), while there was no significant difference between outbound and return legs for southern fulmars (LSM; *z* = 0.32, *p* = .999; Figure 7). Split by species, the interaction between wind speed and trip section was significant only in cape petrels, but not in the other two species (Table 3, Models 4.1.1, 4.1.2 and 4.1.3). ΔDir_fw_ and thus the tailwind component increased with increasing wind speeds on return legs of cape petrels (m4.1.1.2; Figure 7), and thus in agreement with prediction 4.1 (species should adjust their flight direction in relation to wind direction to avoid unfavorable strong headwinds and crosswinds). However, on outbound legs, this relationship was missing for cape petrels (m4.1.1.1; Figure 7), and support for prediction 4.1 was also absent (since the interaction terms were not significant) for Antarctic petrels and southern fulmars.

**TABLE 3:** Modeling approach to test prediction 4, which consisted of three steps. In the first step (upper part of the table), we ran generalized linear mixed models (GLMMs) with ΔDir_fw_ as dependent variable with a binomial error distribution. Models were based on the same dataset as those detailed in Table 1, with identical sample sizes. In the second step (middle part of the table), we ran generalized additive mixed models (GAMMs), which were based on the number of data per hour and bird identified as commuting (as detailed in Methods). The dependent variable was a proportion based on commuting locations per hour and BirdID). Hour of day was included as circular smoothed term. *N* = 3,768 observations in total. In the third step (bottom part of the table), we used linear mixed models to explore whether maximum distance from nest (as dependent variable) was affected by average ΔDir_fw_ on the outbound section of the foraging trip. BirdID was included as random effect. *N* = 196, with *N* = 1 per trip

Model name	Explanatory variables	Interaction terms	Data	Test statistic for variables marked in red	*R* ^2^‐values
Testing prediction 4.1: GLMMs with ΔDir_fw_ as dependent variable					
m4.1_full	wind speed + species+ trip section	all possible 2‐way interactions+ wind speed*species*trip section	All	***F*_2_ = 23.28, *p* < .001**	*R* ^2^_c_ = 0.187; *R* ^2^_m_ = 0.088
m4.1.1	wind speed + trip section	wind speed*trip section	Cape petrel	***F*_1_ = 77.11, *p* < .001**	*R* ^2^_c_ = 0.242; *R* ^2^_m_ = 0.154
m4.1.1.1	wind speed		Cape petrel Outbound legs	*F*_1_ = 0.77, *p* = .459	*R* ^2^_c_ = 0.345; *R* ^2^_m_ = 0.001
m4.1.1.2	**wind speed**		Cape petrel Return legs	***F*_1_ = 19.35, *p* < .001**	*R* ^2^_c_ = 0.270; *R* ^2^_m_ = 0.043
m4.1.2	wind speed + trip section	wind speed*trip section	Antarctic petrel	*F*_1_ = 0.72, *p* = .4275	*R* ^2^_c_ = 0.186; *R* ^2^_m_ = 0.107
m4.1.2_red	**wind speed + trip section**		Antarctic petrel	***F*_1_ = 18.85, *p* < .001; *F*_1_ = 38.74, *p* < .001**	*R* ^2^_c_ = 0.169; *R* ^2^_m_ = 0.094
m4.1.3	wind speed + trip section	wind speed*trip section	Southern fulmar	*F*_1_ = 0.02, *p* < .882	*R* ^2^_c_ = 0.095; *R* ^2^_m_ < 0.001
m4.1.3_red	wind speed + trip section		Southern fulmar	*F*_1_ = 0.03, *p* = .867; *F*_1_ = 0.06, *p* = .810	*R* ^2^_c_ = 0.095; *R* ^2^_m_ < 0.001
Testing prediction 4.2: GAMMs with proportion data as dependent variable					
m4.2.1	species + trip section + s(Hour of day)	species*trip section + s(Hour)*species + s(Hour)*trip section	All proportion data	Dev = −1.52, *p* = .475; Dev = −4.03 *p* = .257; **Dev = −74.36, *p* < .001**	*R*_adj_ = 0.130
m4.2.1_red	species + trip section + s(Hour of day)	s(Hour)*trip section	All proportion data	**Dev = −75.30, *p* < .001**	*R*_adj_ = 0.124
m4.2.2	species + wind category + s(Hour of day)	species*wind category + s(Hour)*species + s(Hour)*wind category	All proportion data	Dev = −8.57, *p* = .073; Dev = 2.81, *p* = .180; **Dev = −9.16, *p* = .049**	*R*_adj_ = 0.113
m4.2.2_red	species + wind category + s(Hour of day)	s(Hour)*wind category	All proportion data	**Dev = −10.58, *p* < .040**	*R*_adj_ = 0.101
Testing prediction 4.3: LMMs with maximum distance from nest as dependent variable					
m4.3.1_full	species + ΔDir_fw_ (average over outbound trip section)	species*ΔDir_fw_ (averaged over outbound section for each trip)	All	*F*_2_ = 0.06, *p* = .940	*R* ^2^_c_ = 0.296; *R* ^2^_m_ = 0.239
m4.3.1_red	**species + **ΔDir_fw_ (average over outbound trip section)		All	***F*_2_ = 19.53, *p* < .001**; *F*_1_ = 3.54, *p* = .061	*R* ^2^_c_ = 0.299, *R* ^2^_m_ = 0.248

**FIGURE 8:**
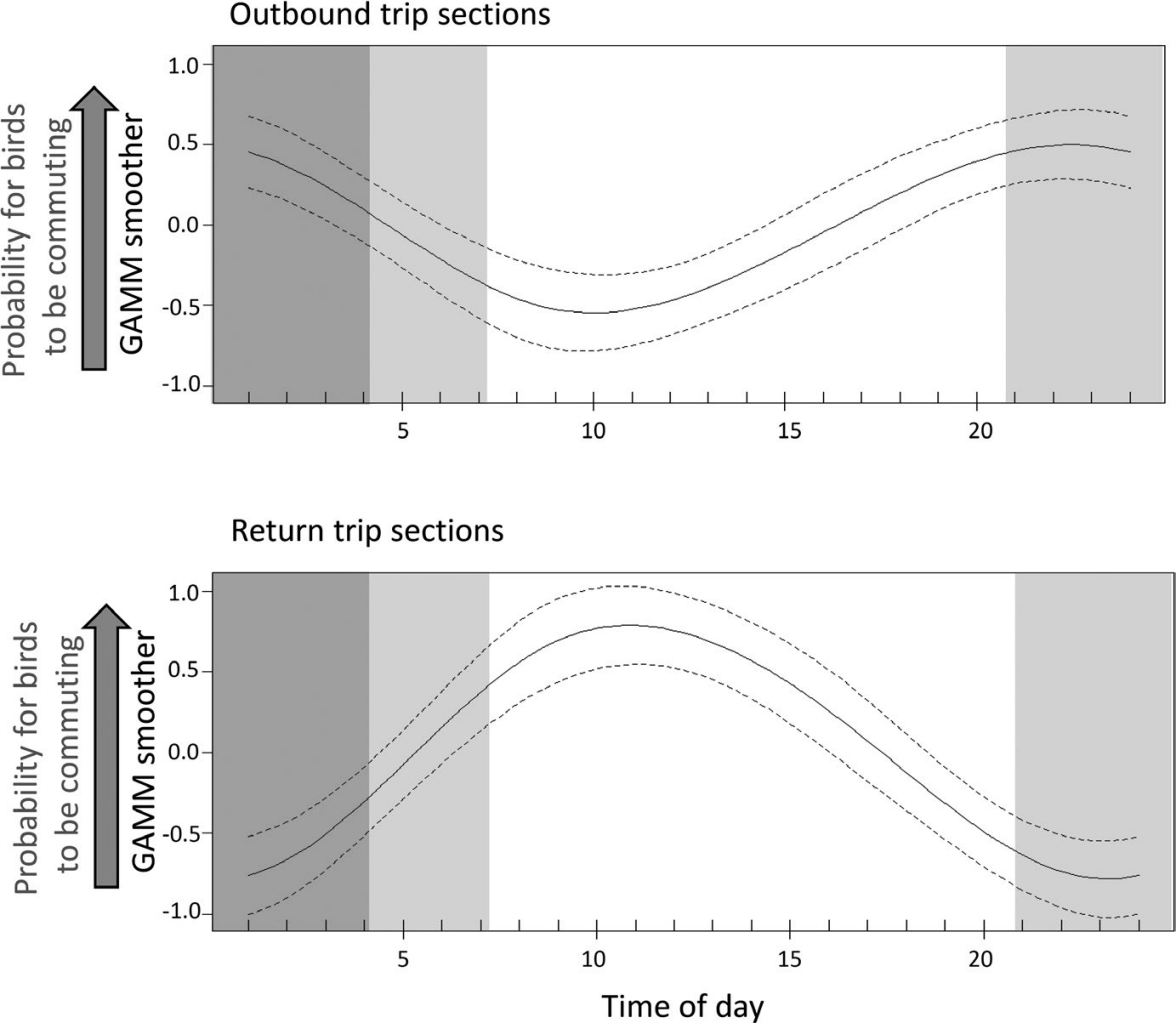
Outputs of the generalized additive mixed model (m4.2.1_red; see Table 3) illustrating the significant interaction effects between time of day and trip section (outbound vs. return legs). GAMMs were run with the proportion of birds being on either an outbound or return leg as dependent variable. Since the species*time‐of‐day effect was not significant (Results), we used the same model for all three species. Light gray background reflects maximum twilight times, and dark gray background reflects maximum periods of darkness (only experienced by southern fulmars at the end of the chick‐rearing period)

**FIGURE 9:**
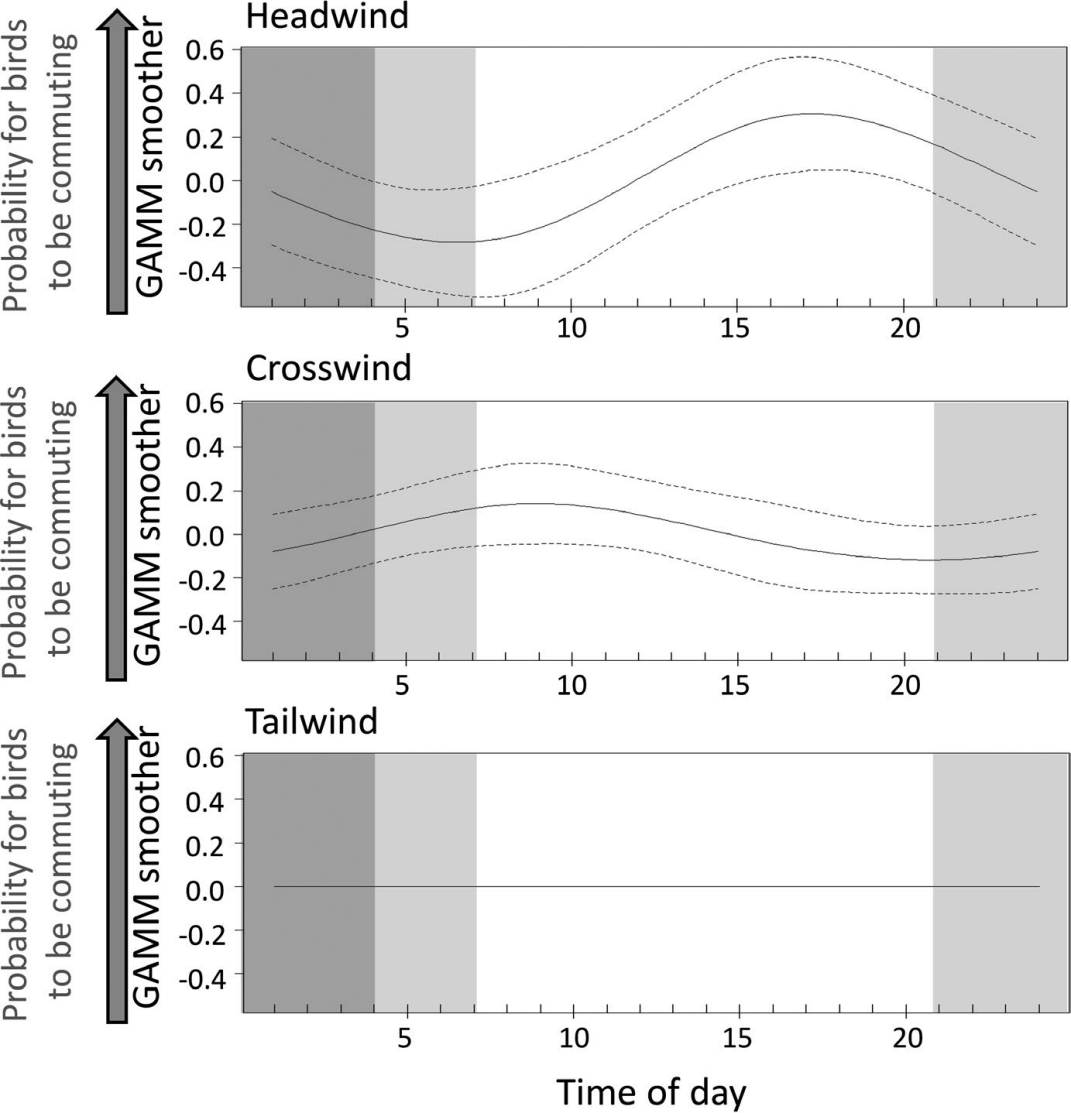
Outputs of the generalized additive mixed model (m4.2.2_red; see Table 3) illustrating the significant interaction effects between time of day and wind category (i.e., headwind, crosswind, and tailwind) on the proportion of birds commuting per hour. Since the species*time‐of‐day effect was not significant (Results), we used the same model for all three species. Light gray background reflects maximum twilight times, and dark gray background reflects maximum periods of darkness (only experienced by southern fulmars at the end of the chick‐rearing period)

GAMMs to test prediction 4.2 (commuting trips should show a distinct pattern in relation to the diurnal wind patterns so that birds can avoid unfavorable winds) reflected that outbound and return legs were not uniformly distributed across daylight hours (Figure 8). The timing of outbound and return legs did not differ significantly between species (Table 3, m4.2.1), but did significantly differ with hour of the day (significant 2‐way interaction between trip section with time of day; Table 3, m4.2.1_red). The probability of birds being on outbound legs appeared to visually match the hours of the day with higher wind speeds, and the timing of return legs coarsely matched the hours of the day with the lowest wind speeds (Figure 8). Birds experienced headwinds, tailwinds, or crosswinds at different times of the day, and this did not differ significantly between species (nonsignificant interaction term between species and wind; Table 3; m4.2.2 and m4.2.2_red; Figure 9). All three species experienced headwinds mostly over midday and in the afternoon, and thus in the hours of the day when coastal katabatic winds are typically lowest. Crosswinds were experienced mostly in the morning hours, coinciding with the time when coastal katabatic winds are starting to decline. There was no distinct daytime pattern for encountering tailwinds (Figure 9).

Finally, average ΔDir_fw_ on outbound legs had no significant effect on the maximum distance from nest that birds reached on foraging trips (Table 3, models m4.3.1_full and m4.3.1_ red). This result contradicted prediction 4.3, under which we expected birds to limit their trip distance when encountering headwinds on the outbound leg.

### 3.4 Past and future wind conditions in the study area

**FIGURE 10:**
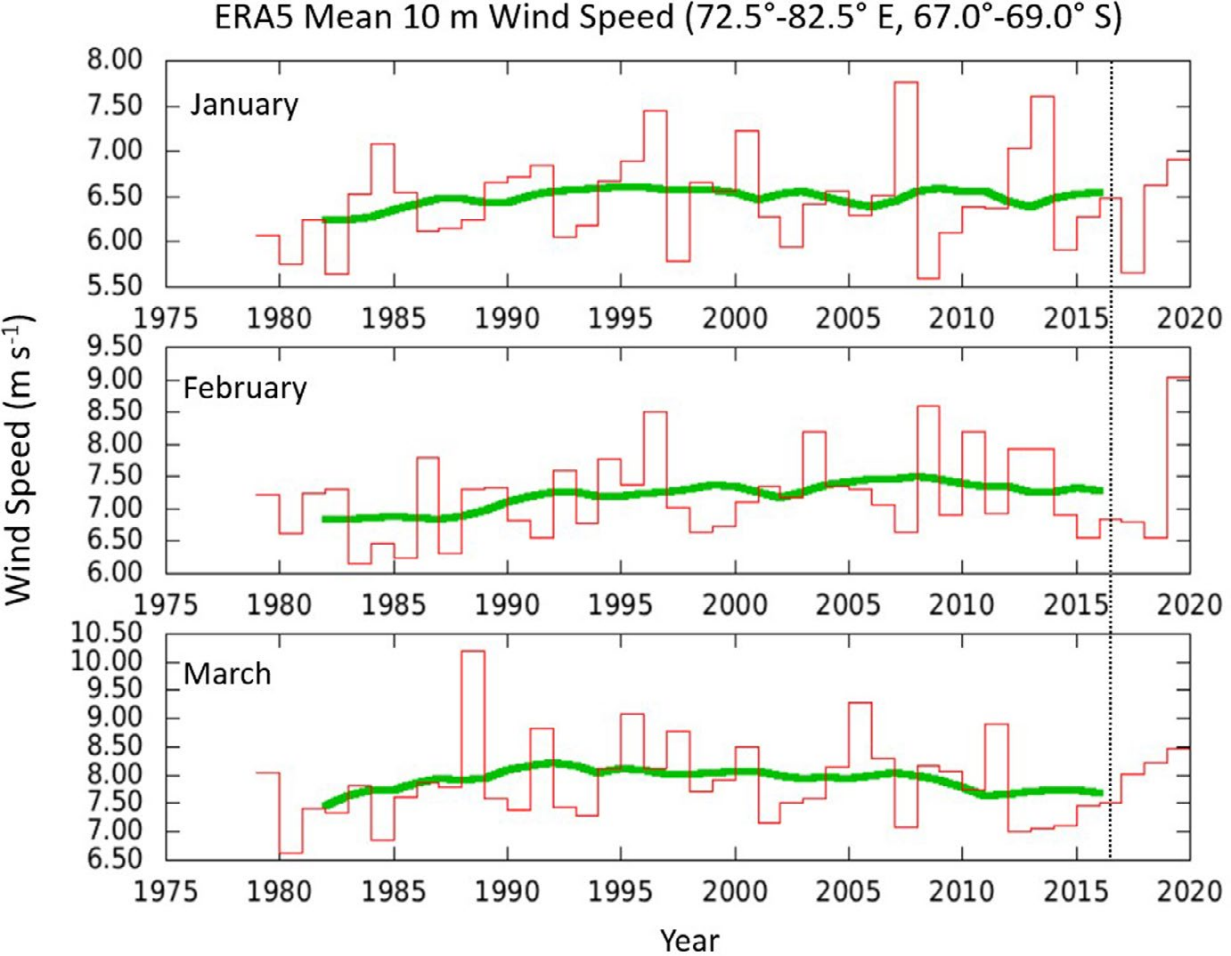
Monthly mean 10‐m wind speed from the ERA5 reanalysis averaged over the region 72.5°–82.5°E, 67.0°–69.0°S. Shown are monthly averages for January, February, and March for years 1979–2019 (red histogram) and a 10‐year running mean (green line). The year 2016 (study year) is highlighted by the vertical dotted line. Figure produced using data and tools available through the KNMI Climate Explorer (https://climexp.knmi.nl/)

Linear trends for wind speed at 10m height above sea level over the time period 1979–2019 based on the ERA5 reanalysis were not significant in any month (linear regression, all *p* > .91). For our particular study year, 2016, the monthly mean near‐coastal winds were evidently fairly typical of the average during the preceding 4 decades (Figure 10).

**FIGURE 11:**
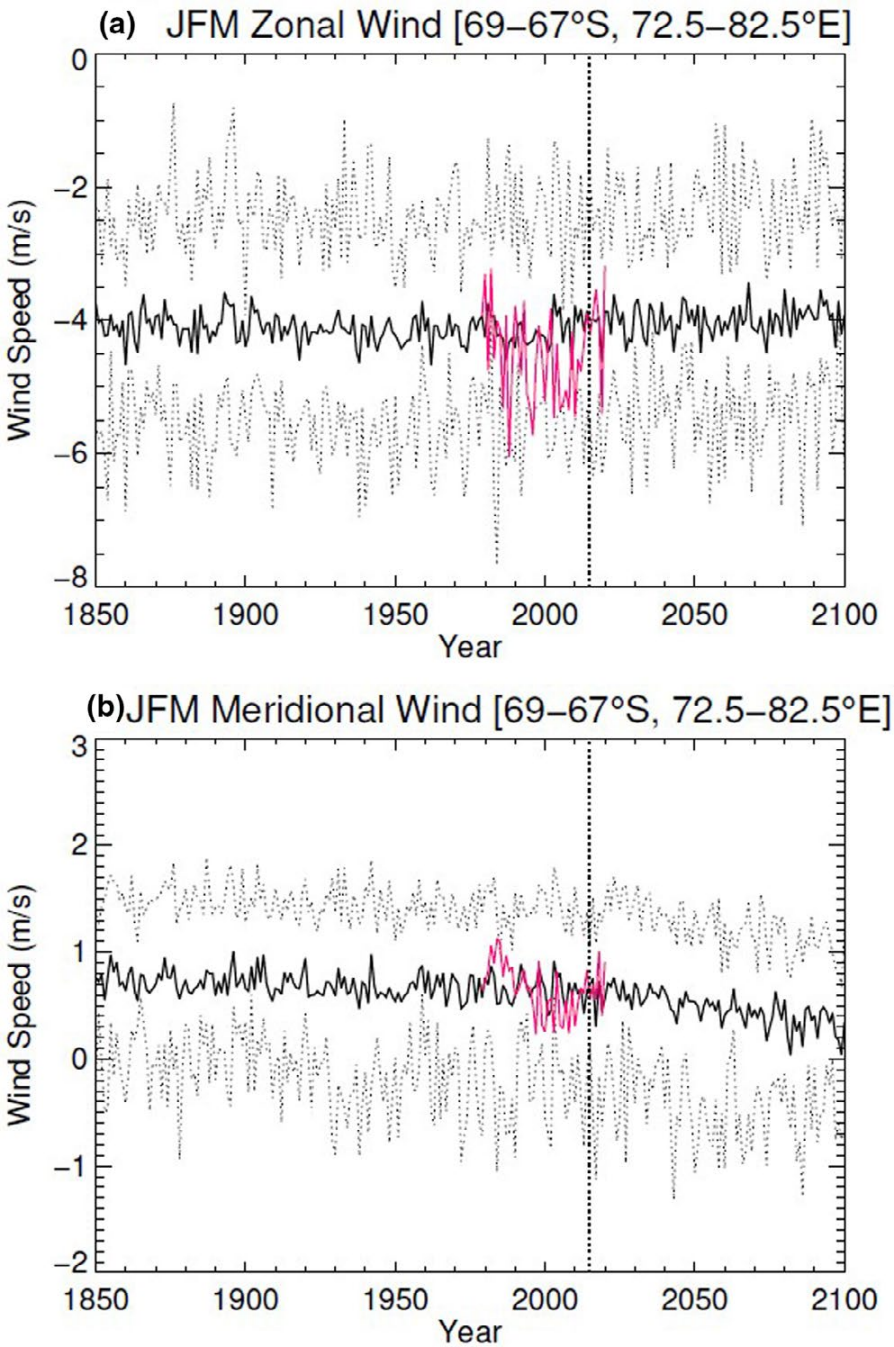
Comparison of mean January to March (JFM) 10‐m wind components from CMIP6 simulations with ERA5 reanalysis. (a) Meridional wind speed, that is, wind speed along the north–south direction. Positive values indicate southerly winds. (b) Zonal wind speed, that is, wind speed along the east–west direction. Negative values indicate easterly winds. The solid black line shows the average over the set of the first ensemble member from each of 8 CMIP6 model simulations; the red solid line shows ERA5 values for 1979–2020. The vertical dashed line marks 2015; before 2015, the CMIP6 models use “historical” (observed) forcings; from 2015 onwards, the models use the ssp585 high‐emissions scenario (Eyring et al., 2016). The dashed lower and upper lines show the 10th and 90th percentiles over the set of CMIP6 model members. Note that the vertical scales in the panels are different

There was no evidence for a trend in the easterly surface wind component in simulations for the highest emissions scenario using the CMIP6 models (Figure 11a). While there was a difference in the strength of the mean zonal wind component (i.e., the wind in the east–west direction) across models compared with the ERA5 reanalysis (ERA5 ‐ CMIP6 = 1.1 ± 0.9 m/s averaged over 1979–2019, which is significant, *p* < .001; Student's *t* test), none of the models showed any significant trends in this component. For the surface meridional wind component (i.e., the wind in the north–south direction), there was also a significant difference between ERA5 and the multimodel mean in the historical period (ERA5 ‐ CMIP6 = 0.9 ± 0.5 m/s averaged over 1979–2019, *p* < .001; Student's *t* test). However, a future decrease was apparent, with an overall trend toward weaker southerly winds (i.e., less positive values) toward the end of the century (Figure 11b). The linear trend in the CMIP6 multimodel mean is 3.1 ± 1.3 m/s per century over 2000–2100 (*p* < .001).

## 4 DISCUSSION

### 4.1 Ground speed in relation to wind speed and differences within and between species

In agreement with our prediction 1, ground speeds in all three species increased with wind speed under tailwinds, but decreased under headwinds, which matches previous observations in fulmarine petrels and albatrosses (Pennycuick, 1982; Spear & Ainley, 1997b; Wakefield et al., 2009). Antarctic petrels, and thus the species with the highest wing loading, showed the steepest response of ground speed in response to wind speeds, matching prediction 2. We had further expected that cape petrels, the species with the lowest wing loading, lowest body mass, and lowest aspect ratio, would show the weakest response of ground speed in response to wind speed among the three species, but instead, cape petrels turned out to be intermediate between Antarctic petrels and southern fulmars. This is an interesting result and may highlight the importance of other morphological or behavioral aspects besides wing loading and aspect ratio for flight behavior and utilization of winds. Among the three study species, southern fulmars had the highest body mass but showed intermediate wing loading and also intermediate aspect ratios (Table 1). This was due to the wing area of southern fulmars being comparatively larger, due to a wider wingspan and broader wings (i.e., longer primary and secondary feathers) compared with Antarctic petrels (N. Dehnhard & L. Emmerson, unpublished data), resulting in the lower wing loading and aspect ratio of southern fulmars compared with Antarctic petrels. One possible explanation for our finding could be that southern fulmars fly differently under head‐ or tailwinds than the other two species (e.g., in a different flight mode, such as using flapping flight in a different way). To test whether this is the case, one would need higher precision GPS data, ideally in combination with accelerometer data.

Within species, we found that under tailwinds, wind speeds had a greater effect on ground speeds on return than on outbound legs, matching our prediction (3) that if parental birds foraged successfully, their increased body mass should affect wing loading and thus flight characteristics. We did not measure body mass of adults before and after their foraging trips, and therefore have to assume that foraging trips of adults were—at least in most cases—associated with a weight gain, either due to self‐feeding or since parents brought food to their chicks. This is corroborated by the fact that chicks of logger birds appeared to grow normally, chick survival was high in cape petrels and southern fulmars (lower in Antarctic petrels due to predation), and we did not observe any chicks that starved; hence, foraging success did not seem to limit breeding success. Previous studies on the three study species have found meal sizes to range between 50 and over 250 g in southern fulmars and Antarctic petrels (Creuwels et al., 2010; Lorentsen, 1996) and between 3 and 55 g in cape petrels (Fijn et al., 2012), values that should affect wing loading of their parents. Our findings for increased ground speeds under stronger tailwinds on return legs also mean that the benefit from tailwinds might be highest on return legs in general, and thus agree with earlier findings that the ideal location of a colony would be downwind from feeding areas (Pennycuick, 1989; Spear & Ainley, 1997a; Tarroux et al., 2016). In Antarctica, ice‐free land that is suitable for breeding is limited, and thus, this ideal condition might be difficult to achieve. For example, where ice‐free areas near the coast is lacking, Antarctic petrels may breed on nunataks located up to 200 km inland, and face unfavorably strong crosswinds on their commute over land (Tarroux et al., 2016). Also in our study system of coastal breeding fulmarine petrels, the birds mostly encountered crosswinds—both on outbound and on return legs of foraging trips (cf. Figure 3 and Appendix S1). Conspicuously, many foraging trips described a loop in clockwise direction, that is, birds flying out in northerly to northwesterly direction, heading eastwards on the middle section of the foraging trip and returning in south to southwesterly direction toward the colony (Appendices S1 and S2). Given the predominant easterly winds, this implies birds mostly face headwinds on the middle (foraging) section of foraging trips, but crosswinds during outbound and return legs. This loop pattern may be beneficial for the commuting part, while they likely encountered headwinds during the middle section, which could reduce flight speed but possibly enhance prey detection: Procellariiforms are olfactory foragers (Nevitt, 1999; Nevitt et al., 2004), and thus, flying into headwinds during fine‐scale search for food may be beneficial (Nevitt et al., 2008).

### 4.2 Adjustment of timing of commutes, flight direction, and distance from colony to wind conditions

Given the significant positive effect of tailwinds on the birds’ ground speeds, and the observed negative impact under headwinds, we expected birds to adjust their flight direction, timing of commutes to/from foraging locations, and/or the maximum distance from nest in response to ambient wind conditions (predictions 4.1–4.3). Overall, we found mixed evidence for these predictions. There was no consistency among species of adjusting their flight direction in response to unfavorable strong headwinds (prediction 4.1), neither on outbound nor on return legs. Thus, Antarctic fulmarine petrels did not adjust their course and thus possibly their foraging location(s) to prevailing wind conditions. This result is in agreement with findings in several albatross species (Wakefield et al., 2009) and black‐legged kittiwakes (*Rissa tridactyla*) (Collins et al., 2020). Desertas petrels, on the other hand, appeared to fine‐tune their course in relation to the prevailing winds (Ventura et al., 2020), possibly due to the fact that—foraging in an oligotrophic, pelagic environment—they had to maximize trip distances to encounter their patchy and scarce prey on route. Antarctic fulmarine petrels in this study also did not adjust the maximum distance from the nest, and thus their commute distance, in relation to the encountered wind directions on outbound legs (prediction 4.3). Both of these results (i.e., not adjusting their course, foraging locations and maximum distance) can be explained by the need of parental birds to provision chicks with sufficient food at regular intervals irrespective of wind conditions. Shortening the foraging trip or adjusting the flight direction to avoid headwinds may result in birds visiting less productive foraging areas, which in turn might increase foraging costs and reduce foraging success (sensu optimal foraging theory; MacArthur & Pianka, 1966). Previous studies in seabirds have highlighted that parental birds will—within their physiological limits—adapt foraging locations and extend trip distances substantially to provision their chicks in years with low local food availability (Burke & Montevecchi, 2009; Dehnhard et al., 2016; Montevecchi et al., 2009). Given the energetic costs for flight in fulmarine petrels are among the lowest compared with other (sea‐)bird species (Pennycuick, 2002, 2008), the costs for flying a longer distance, possibly even against the wind, will be outweighed if the feeding grounds are productive, and thus, foraging success is likely to be high. As such, flying against the wind for one part of the foraging trip may come at a comparatively lower cost for a breeding bird than visiting less productive areas where foraging success is lower. One could expect, though, that birds during the nonbreeding period would be less constrained and adapt their flight direction to wind direction more flexibly, which indeed has been demonstrated in wandering albatrosses (Murray et al., 2003).

A higher proportion of all three species of fulmarine petrels were commuting away from their colony in the early morning hours and afternoon/evening hours when katabatic winds were stronger than during midday. In contrast, return trips in all three species occurred mostly between the late morning and early evening hours, and thus under lower katabatic winds. Remarkably, this same time period (i.e., late morning to early evening hours), and thus low coastal katabatic winds, coincided with birds encountering headwinds most frequently. The pattern for crosswinds was less distinct, and crosswinds were mostly encountered in the morning hours and until midday—and thus matched coarsely the timing of return trips. Finally, there was no diurnal pattern at all for when birds encountered tailwinds. To summarize, our data strongly indicate that commuting legs and the encounter of especially headwinds over the course of the day did not happen at random. Our data further suggest that fulmarine petrels adjust the timing of their outbound and possibly return legs so that they encounter headwinds when katabatic winds tend to be weak but crosswinds when katabatic winds are strong. These results therefore support our prediction that birds would adjust their timing of commutes either to benefit from katabatic winds (under crosswinds on return commutes) or to avoid headwinds (on outbound commutes) (prediction 4.2). Despite this apparent adjustment, we observed individuals of all three species encountering the full range from weak to strong head‐, cross‐, and tailwinds (Figure 3). Katabatic winds can be measured many hundred km away from the coast (Parish & Bromwich, 1991) and are therefore of influence in the entire area utilized by our study species during the chick‐rearing season. However, katabatic winds prevail strongest at the coast and get weaker further out at sea, where they also get disrupted by weather systems (Parish & Cassano, 2003). This is also why we used weather model‐derived wind data at sea to assess the birds’ flight behavior. Thus, although birds may adjust their commuting times to katabatic winds, this does not always work out for them, particularly under a passing storm.

### 4.3 Variability and trends in wind conditions

Like other species at high latitudes, Antarctic fulmarine petrels have evidently adapted to particular environmental conditions that are potentially finely balanced as a consequence of the apparent sensitivity of polar climate to anthropogenic change (Clucas et al., 2014; Descamps et al., 2017). The strength and variability of the near‐surface winds and their interaction with the katabatic flow are therefore relevant in considering whether the energetics of the birds are being positively or negatively impacted under recent conditions, and how this will play out into the future. Based on ERA5 reanalysis data from 1979 to 2019, the linear trends of near‐surface (10 m elevation) wind speed in our study region have been overall stable (Figure 9). As shown by van den Broeke and van Lipzig (2004) in a study using 14 years of high‐resolution regional atmospheric climate modeling, the Antarctic near‐surface climate, including winds, responds to variability in the Southern Annular Mode (SAM). SAM phase and 10 m wind speed to the east of Davis Research Station showed a generally negative significant correlation, and a positive, though not significant, correlation to the west of Prydz Bay and the Lambert‐Amery basin (figure 4b in van den Broeke & van Lipzig, 2004). For the region and time period relevant, no significant correlation is apparent for any month using the monthly SAM index provided by Marshall (2003; Figure 1).

There has been a tendency for a more positive phase of the SAM in recent decades, particularly in summer (Turner et al., 2005, 2014). This trend is expected to continue over the remainder of this century, and become stronger in all seasons. A more positive SAM implies an overall strengthening of the westerly wind over the Southern Ocean and a tendency for the latitude of peak winds to shift more poleward. Other modeling studies have indicated that a more general weakening of the Antarctic coastal easterly winds will occur in all seasons over the remainder of this century (Bintanja et al., 2014b; Bracegirdle et al., 2008). As discussed by Bintanja et al. (2014b), this is a consequence of the reduction in forcing by weather systems interacting with the Antarctic plateau due to the strengthening of the dynamical barrier over the Southern Ocean brought about by the positive tendency in the SAM.

When analyzing the highest emission scenario from the CMIP6 model on future wind patterns in the foraging area of our study populations, we found that easterly winds will generally prevail at similar levels as currently, while the influence of southerly winds will get weaker. Our findings for the easterly winds are in contrast to the general findings of Bracegirdle et al. (2008) and Bintanja et al. (2014b) for coastal Antarctica, and could be a consequence of the specific location of our study. We attribute the decrease in southerly winds to the effects of oceanic surface temperature changes altering the strength of the katabatic outflow in the region (van den Broeke et al., 1997). Alternatively, as shown by Bintanja et al. (2014a), model resolution is important for appropriately simulating the effects of local topography, and the decrease in the meridional wind speed could relate to a change in the interaction of trending winds aloft with the generally low‐resolution topography used in the CMIP6 models considered here.

For our study populations, the predicted decrease in southerly winds (and thus weaker katabatics) might imply less headwinds on return journeys from foraging, but less tailwind/crosswind support on outbound legs. This would translate to increased ground speeds with headwinds on return legs, and thus quicker commutes for parental birds to deliver food to their chicks, but slower outbound legs when traveling to foraging areas. How such changes in wind patterns will overall affect trip durations remains to be seen. To better assess likely impacts on the bird populations in our study area from long‐term trends and interannual variability of climate and winds in particular, further use of detailed regional climate modeling is required.

## 5 CONCLUSIONS

We demonstrated the effect of wind speeds and wind direction on the ground speeds of three species of fulmarine petrels on their commutes to and from foraging areas. Our results not only emphasize the importance of wind speed and direction for this group of gust‐soaring seabirds, but also highlight differences between species, some of which are not fully explained by morphological differences in wing loading and aspect ratio. While all three species benefitted from tailwinds, birds did not adjust their flight paths to the prevailing wind directions. However, our data suggested that birds adjusted the timing of outbound and return commutes to the diurnal katabatic winds in order to avoid strong headwinds and benefit from tailwinds and possibly crosswinds.

Our results are highly relevant in the context of a changing environment. While winds are necessary for the energy‐efficient gust‐soaring flight style of Antarctic fulmarine petrels, future changes in the diurnal katabatic wind patterns might on the one hand benefit birds since they will experience less headwinds on return journeys from foraging, but on the other hand, less tailwind/crosswind support on outbound legs might increase flight costs. The impact of these changes for the seabird populations are difficult to estimate.

## CONFLICT OF INTEREST

The authors declare they have no conflict of interest.

## AUTHOR CONTRIBUTION

**Nina Dehnhard:** Conceptualization (lead); Formal analysis (lead); Funding acquisition (equal); Investigation (equal); Methodology (lead). **Andrew R. Klekociuk:** Conceptualization (supporting); Formal analysis (supporting); Investigation (supporting); Methodology (supporting). **Louise Emmerson:** Conceptualization (equal); Data curation (supporting); Formal analysis (supporting); Funding acquisition (lead); Investigation (equal); Methodology (equal); Project administration (lead).

## Supporting information

Appendix S1

Appendix S2

## Data Availability

Biologging data are publically available through the Australian Antarctic Data Centre: https://data.aad.gov.au/metadata/records/AAS_4087_Fulmarine_petrel_tracking_study_Hop_Island_2015_16 or under https://doi.org/10.26179/5d083c180d2b7.
