# Supplementary material for: Interactive effects of body mass changes and species‐specific morphology on flight behavior of chick‐rearing Antarctic fulmarine petrels under diurnal wind patterns
Source: Ecol Evol. 2021 Apr 6;11(9):4972–91. doi: 10.1002/ece3.7501 (PMC8093695; doi:10.1002/ece3.7501)

Supplement 1, Dehnhard et al.

GPS-tracks of birds, shown separately by outbound & return trip sections. Dive locations and GPS time-stamps that were associated with resting were excluded as for the analyses (see Methods). Rosette plots give histograms of the wind direction at Davis Station (black bars) and histograms of the birds' flight direction (grey bars). The projection of maps is polar stereographic. Shades of blue reflect sea ice concentration as shown in the legend. Sea ice maps were obtained from the data archive of the University of Bremen; Spreen, Kaleschke, & Heygster, 2008) from the middle of the respective breeding stage of each species.

Cape petrels - outbound

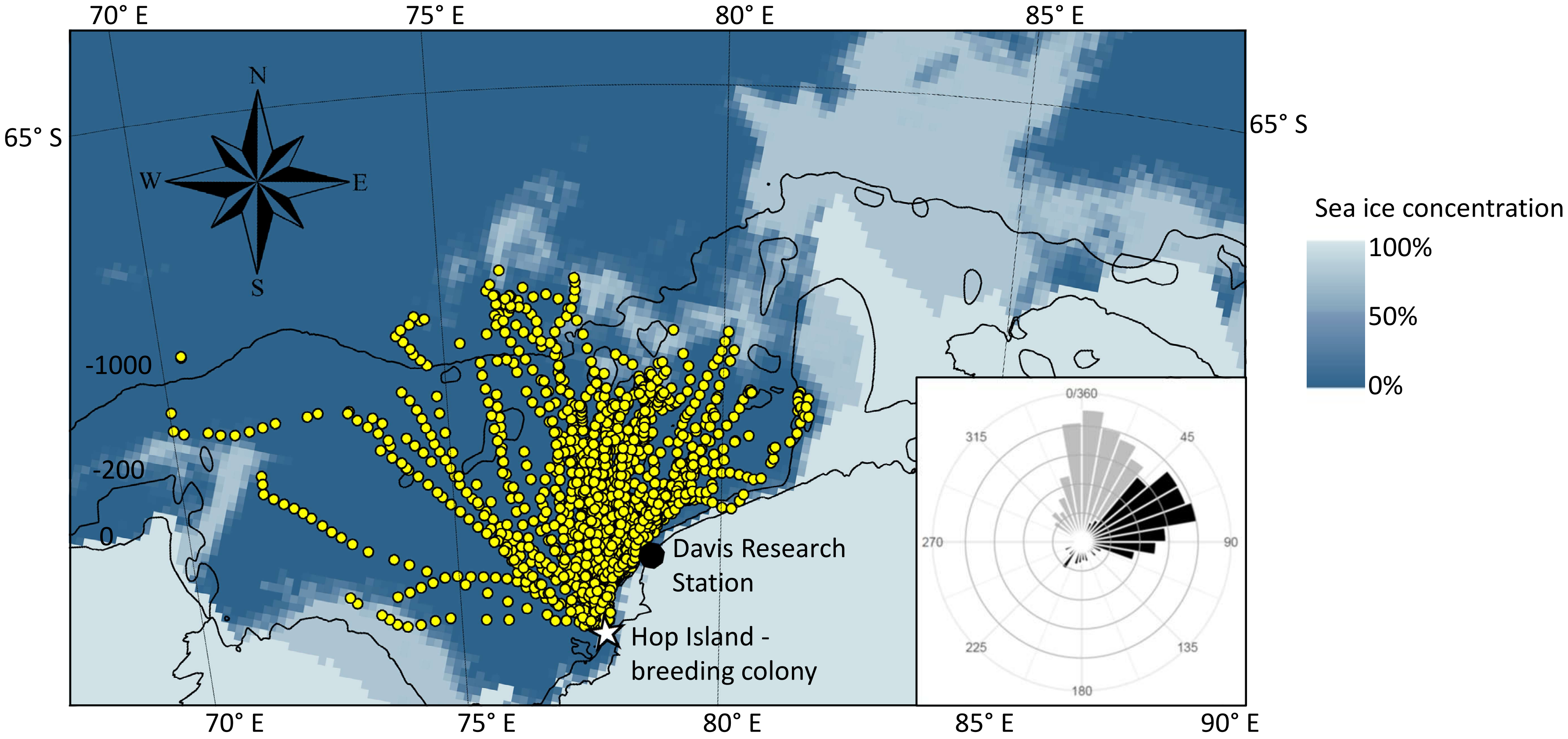

Cape petrels - return

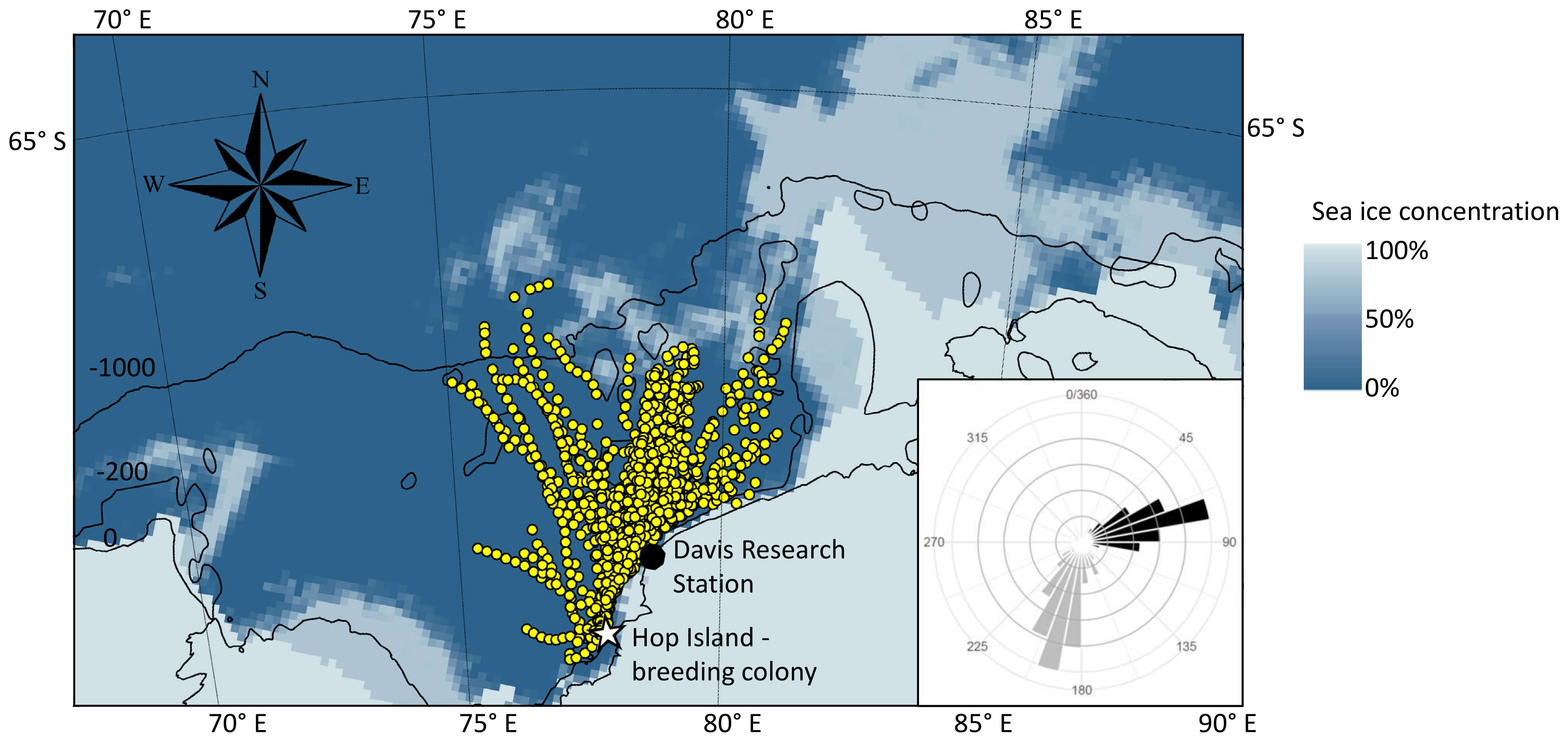

Antarctic petrels - outbound

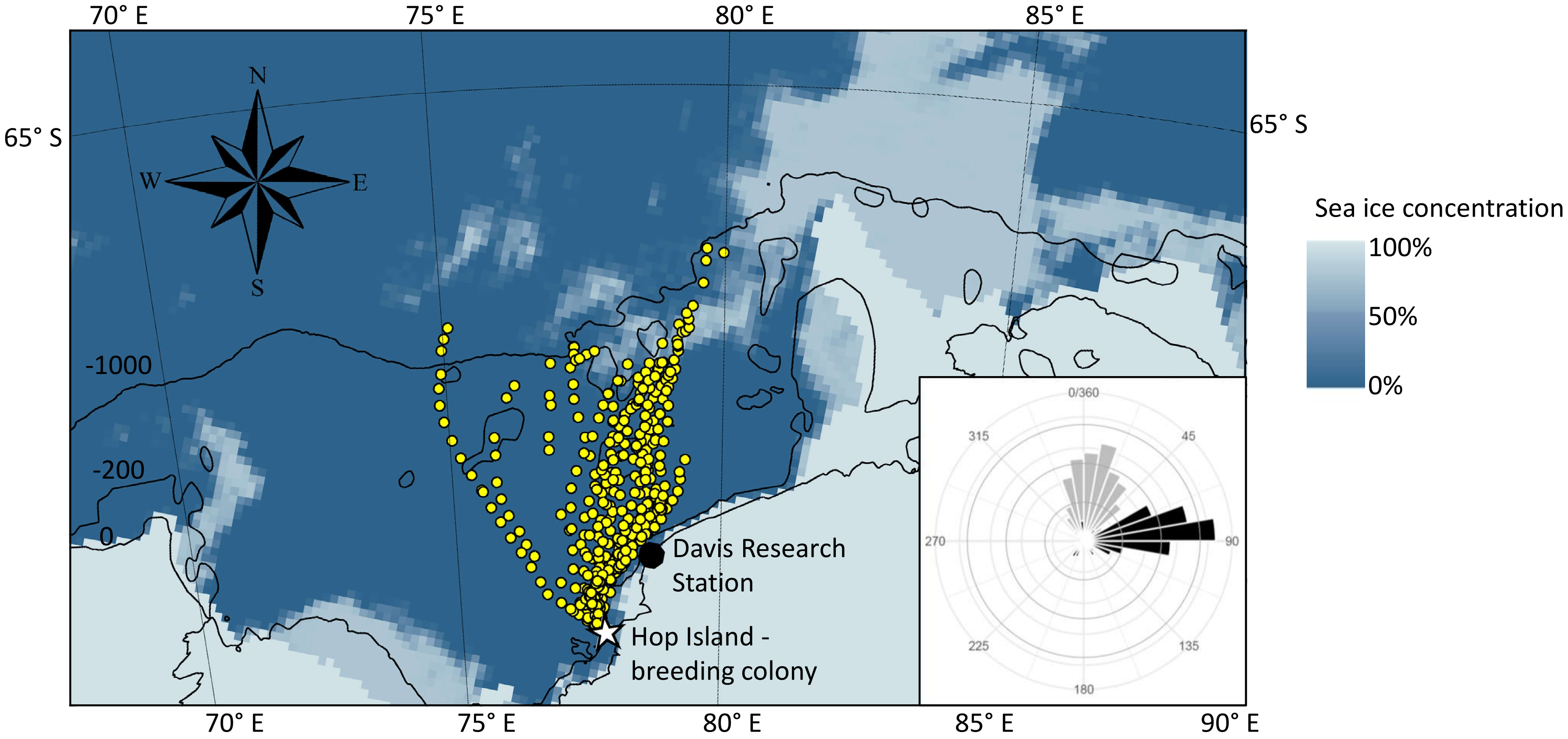

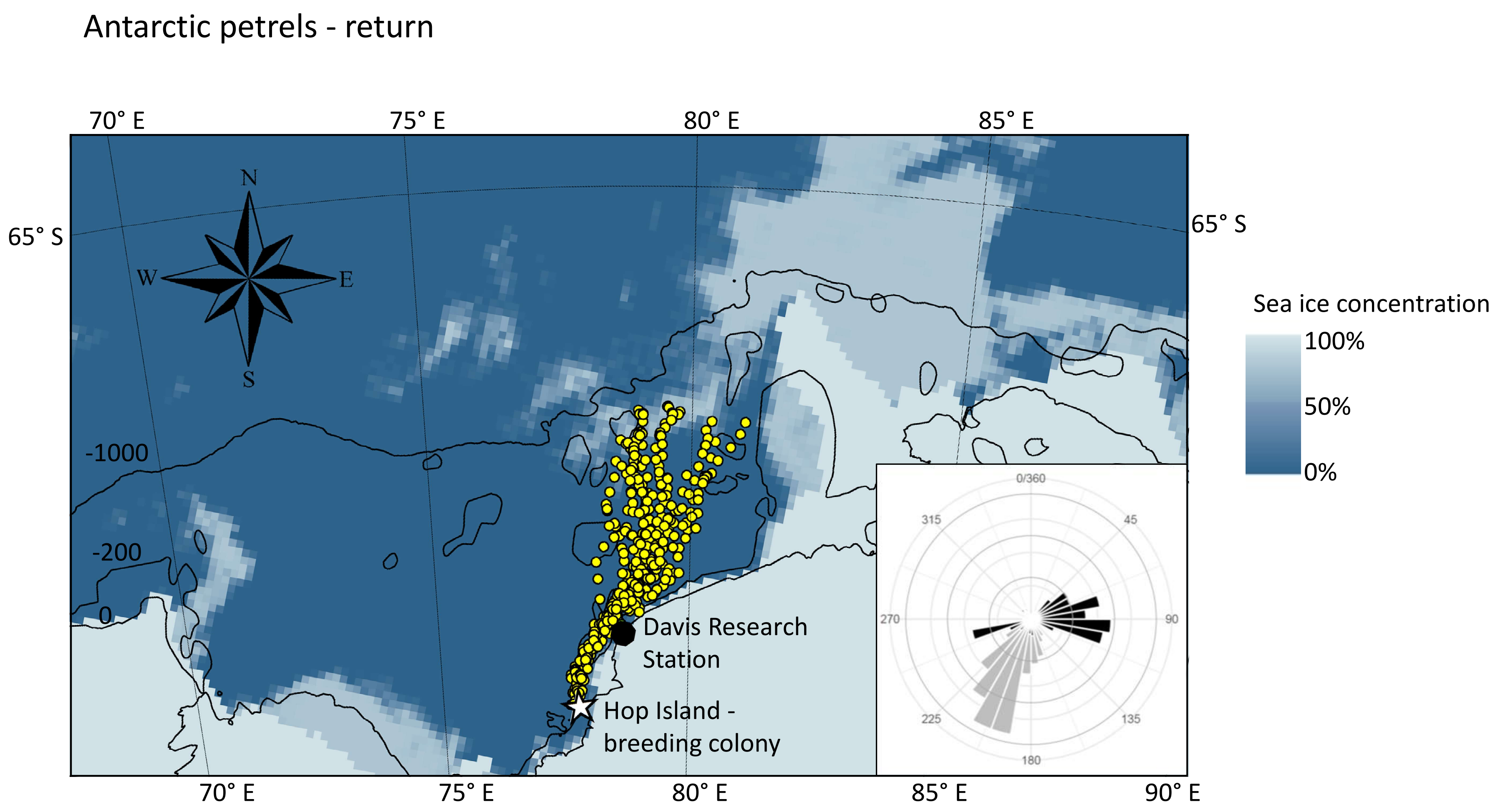
Antarctic petrels - return
70° E
75° E
80° E
85° E
65° S
65° S
N
W
E
S
-1000
-200
0
Davis Research Station
Hop Island - breeding colony
Sea ice concentration
100%
50%
0%
0/360
315
45
270
90
225
135
180
70° E
75° E
80° E
85° E
90° E

Southern fulmars - outbound

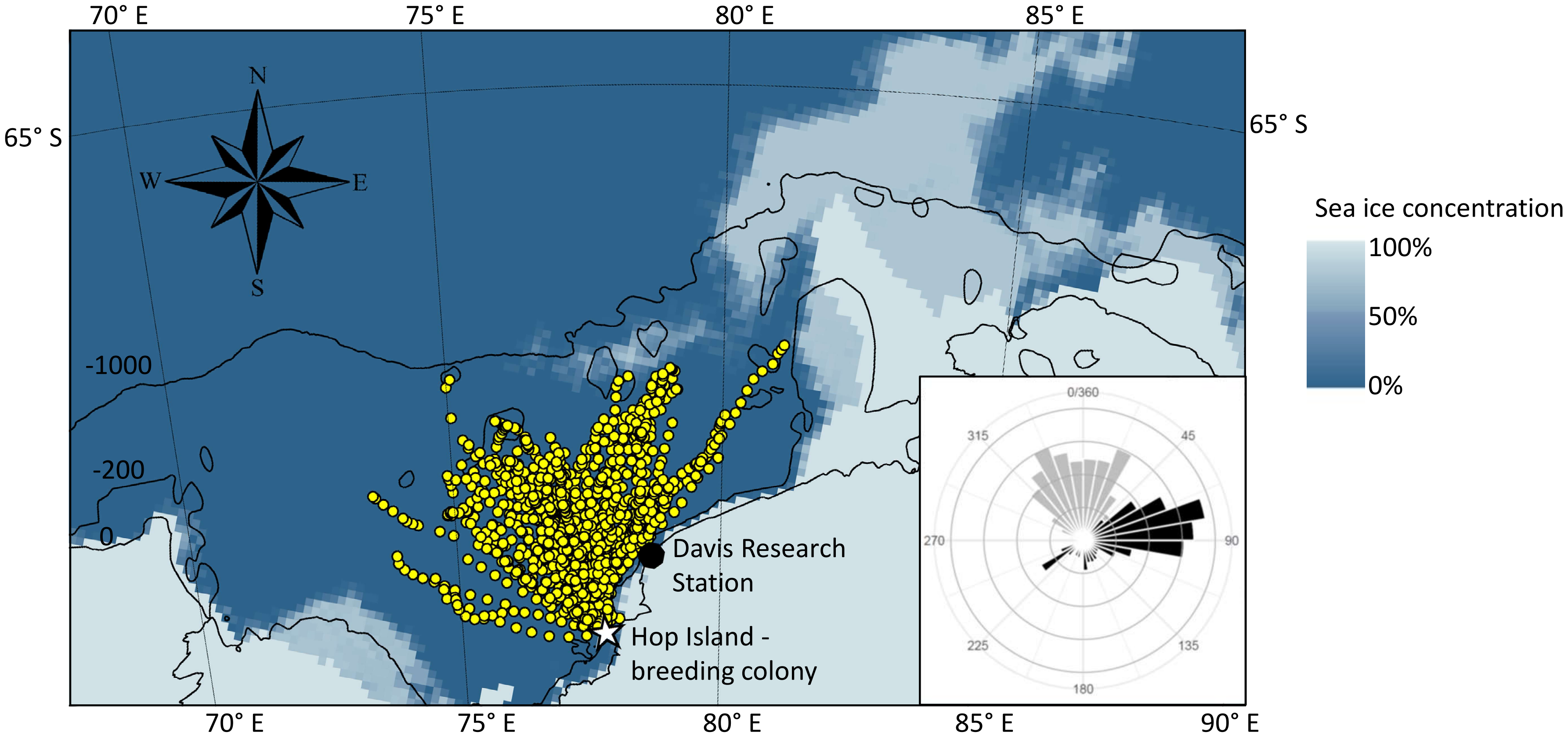

Southern fulmars - return

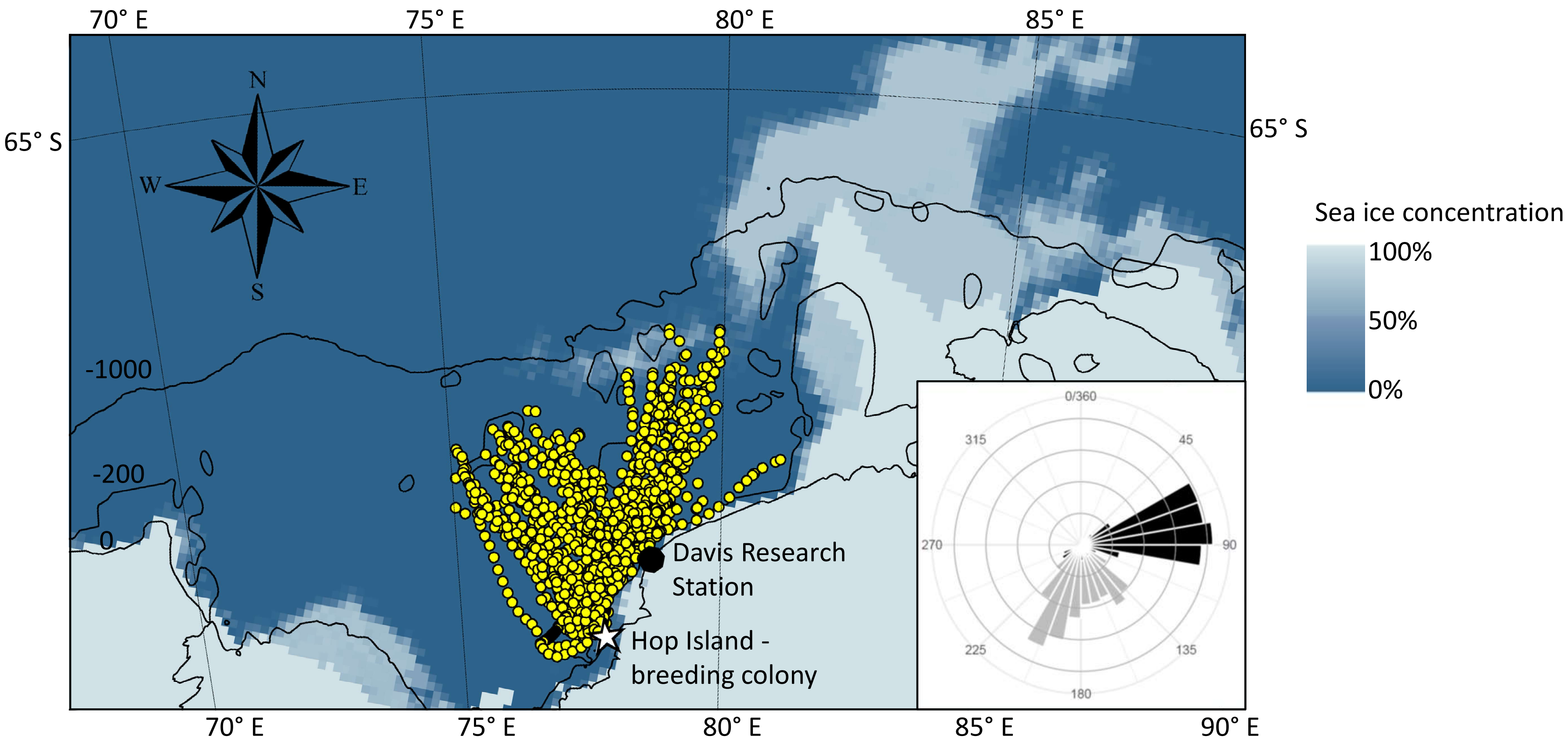

Supplement: Supplementary file 1 — Appendix S1 [file ECE3-11-4972-s002.pdf]
