# Supplementary material for: Interactive effects of body mass changes and species‐specific morphology on flight behavior of chick‐rearing Antarctic fulmarine petrels under diurnal wind patterns
Source: Ecol Evol. 2021 Apr 6;11(9):4972–91. doi: 10.1002/ece3.7501 (PMC8093695; doi:10.1002/ece3.7501)

Supplement 2, Dehnhard et al.

Complete GPS-tracks of individual birds, including those GPS time-stamps that were associated diving and resting (see Methods). White dots reflect GPS time-stamps on outbound, red on middle and grey on return trip sections, respectively. Note that outbound, middle and return legs were defined on the population level and not on a trip-to-trip level, based on maximum distance reached and the proportion of the total trip time (see Methods). The projection of maps is polar stereographic. Shades of blue reflect sea ice concentration as shown in the legend. Sea ice maps were obtained from the data archive of the University of Bremen; Spreen, Kaleschke, & Heygster, 2008) from the middle of the respective breeding stage of each species.

Cape Petrel 01

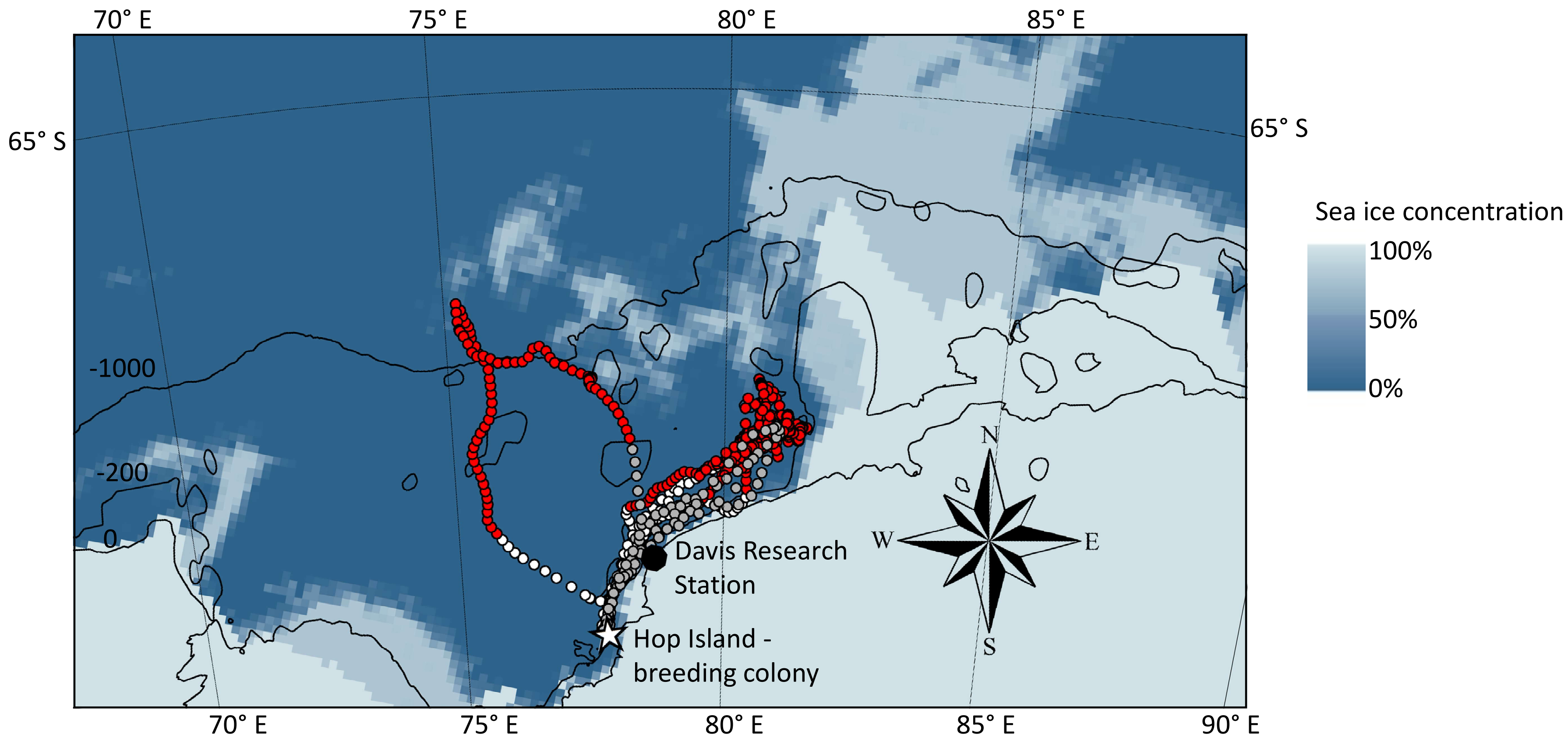

Cape Petrel 03

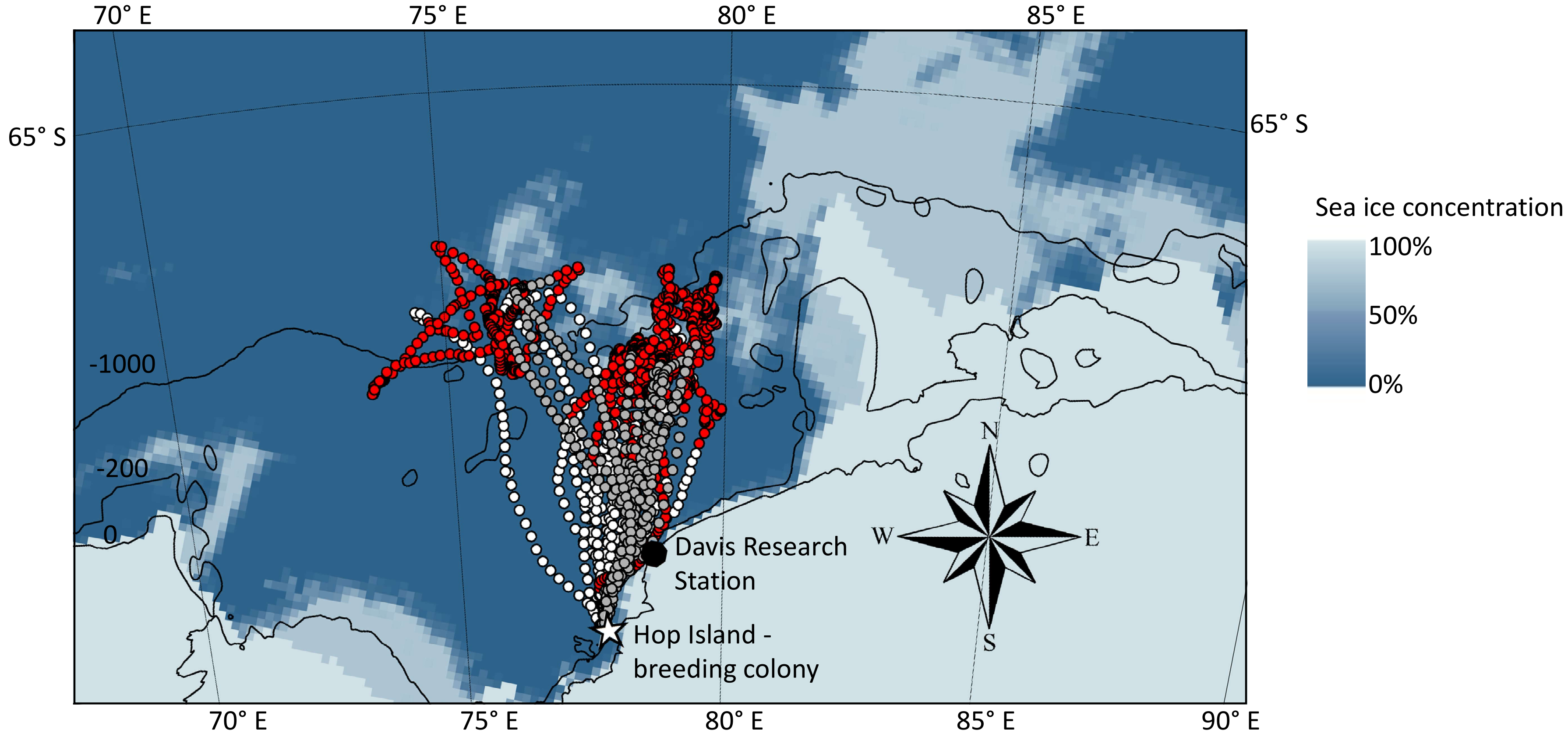

Cape Petrel 04

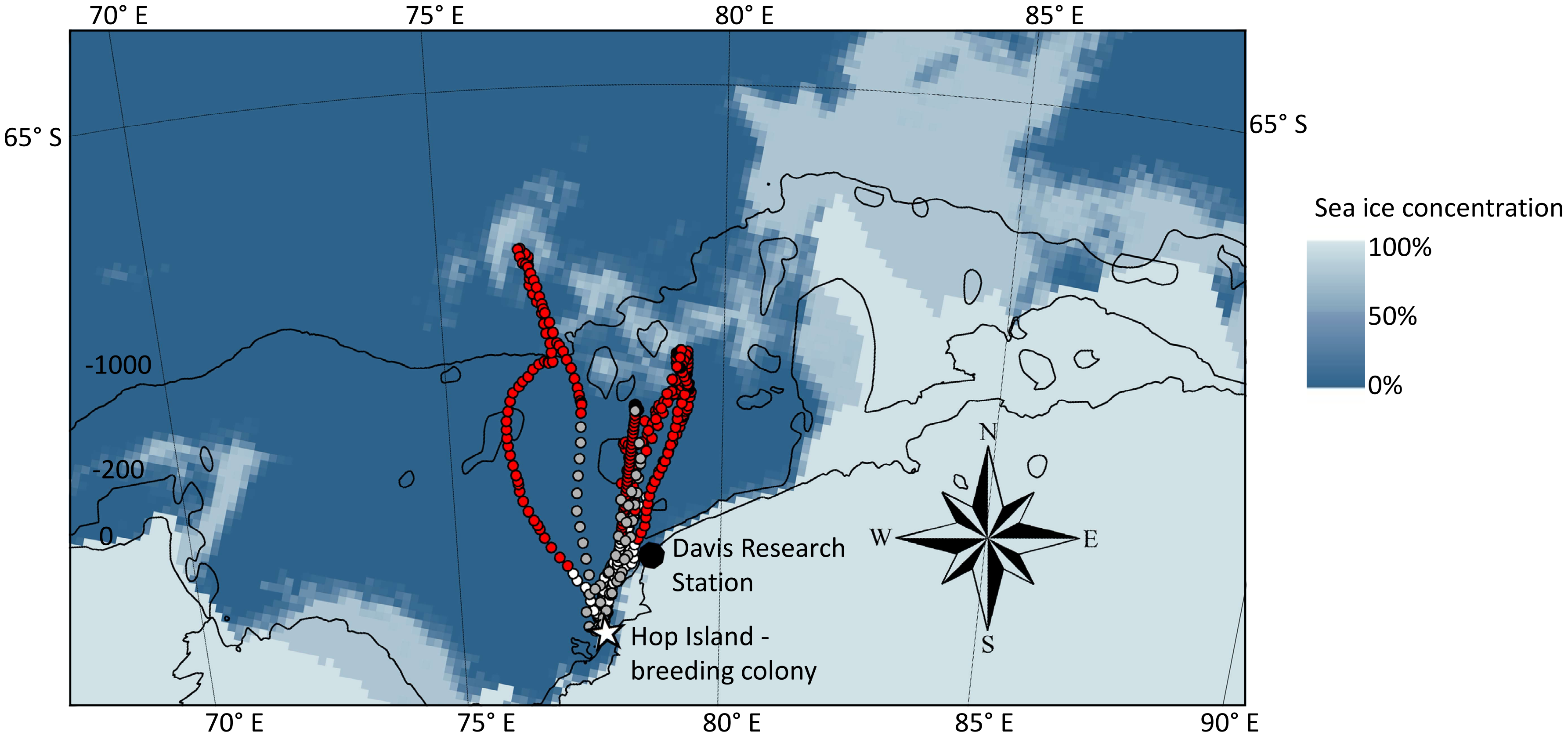

Cape Petrel 10

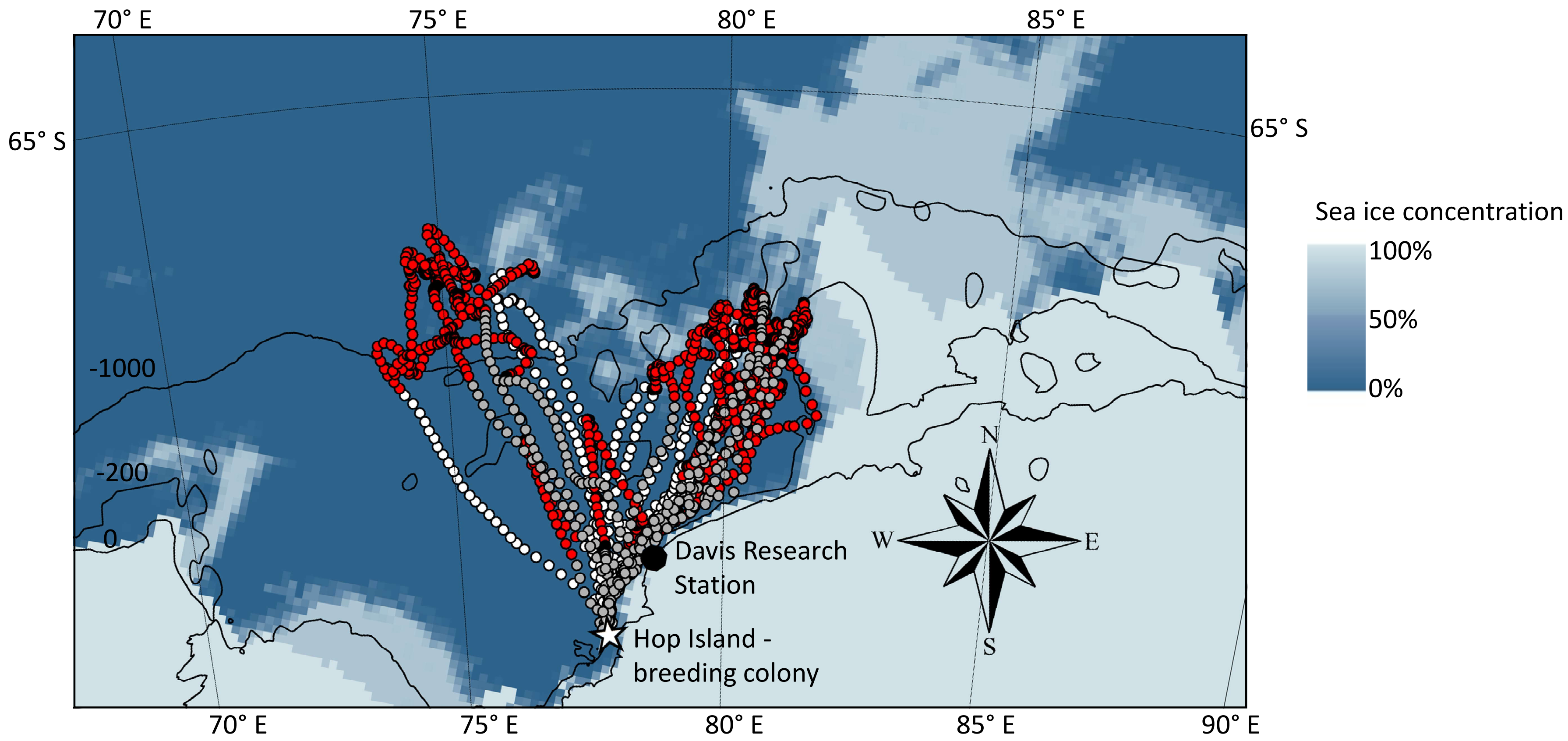

Cape Petrel 11

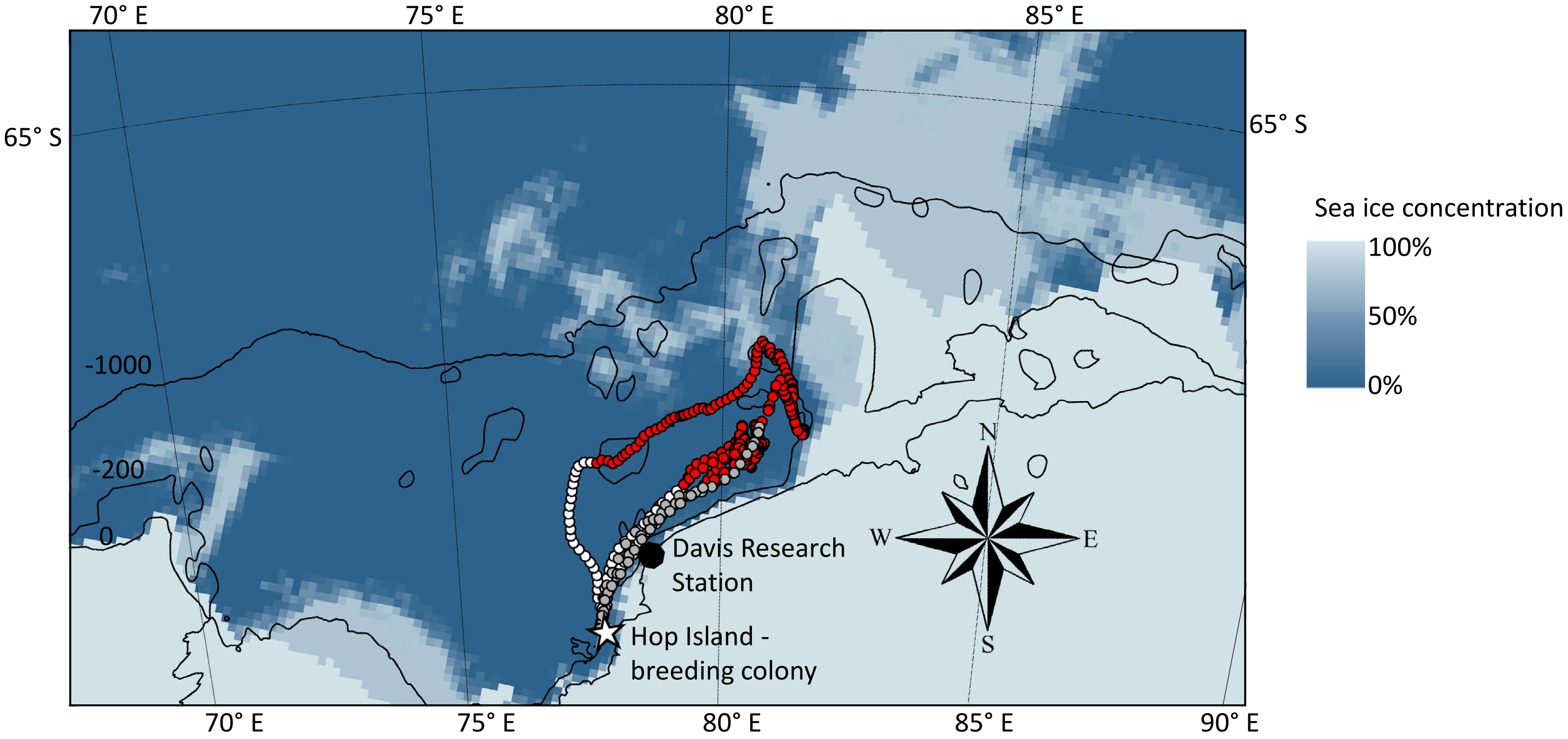

Cape Petrel 13

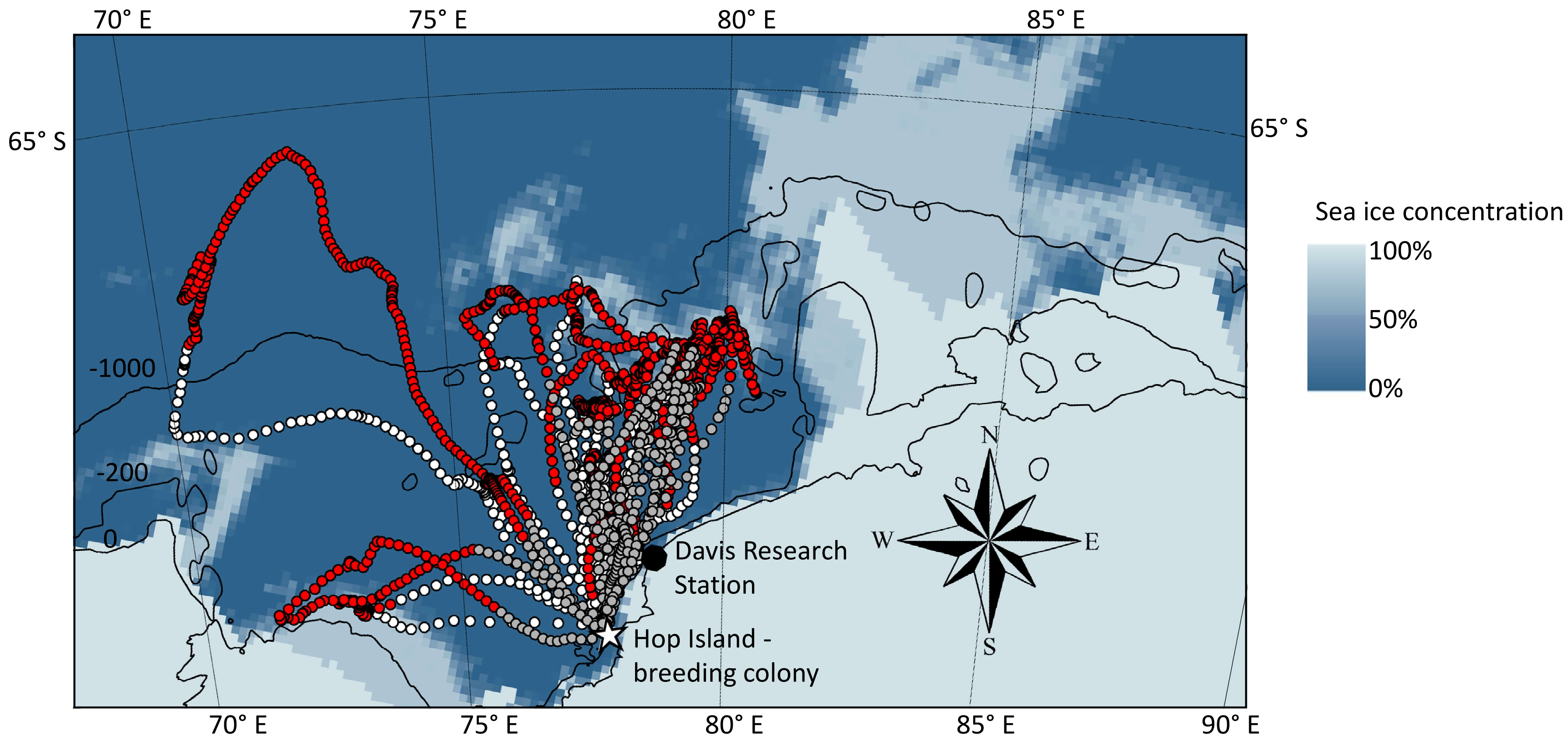

Cape Petrel 14

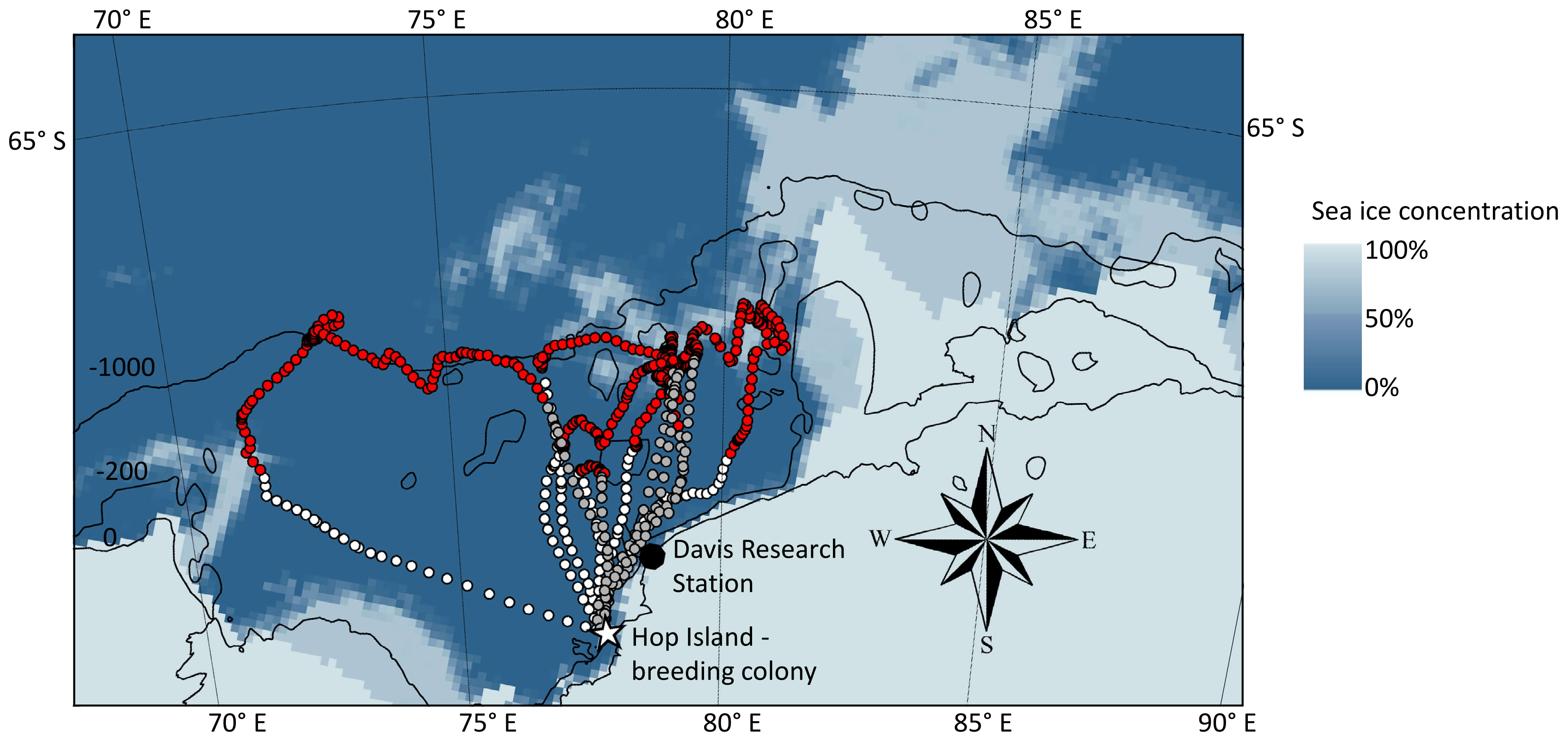

Cape Petrel 15

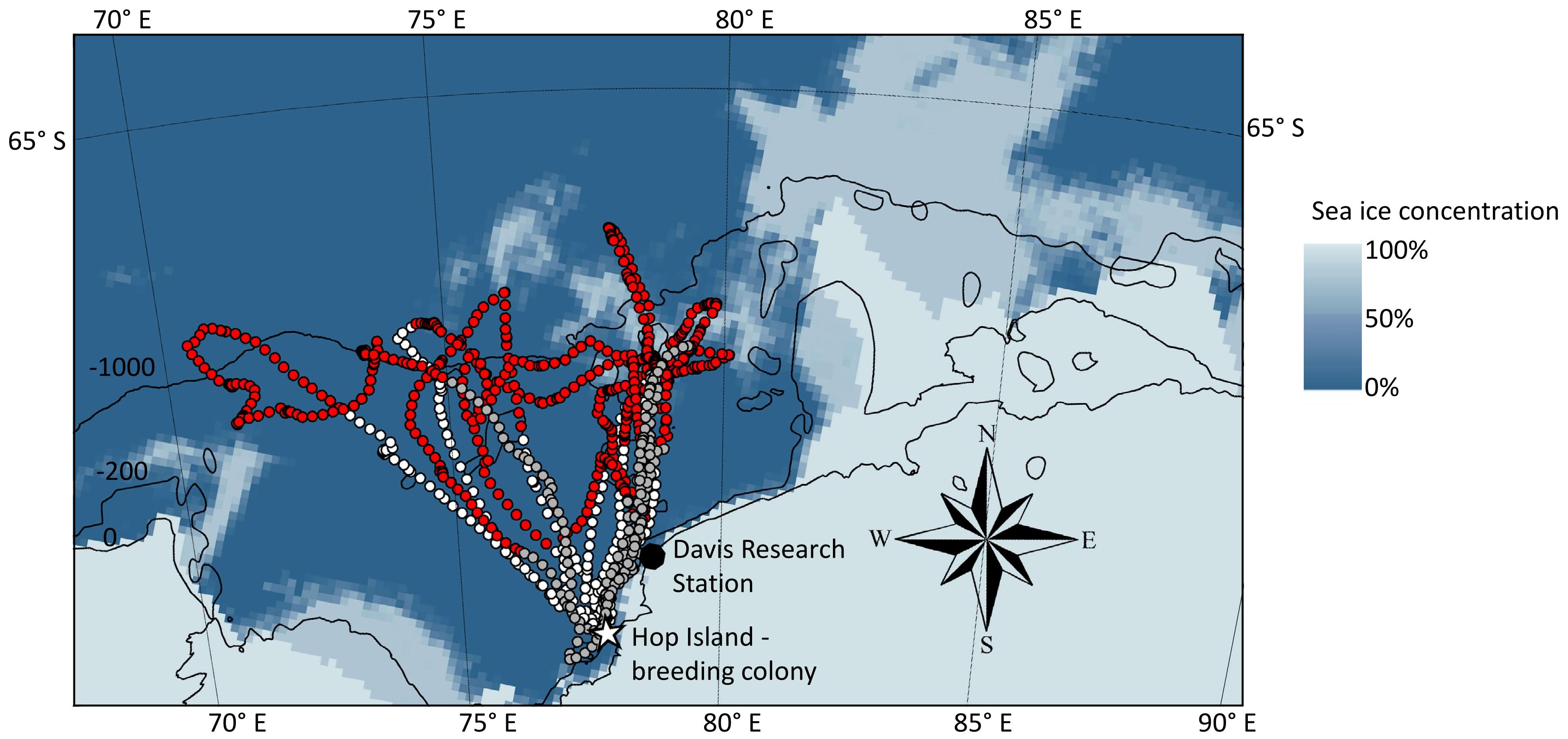

Cape Petrel 11

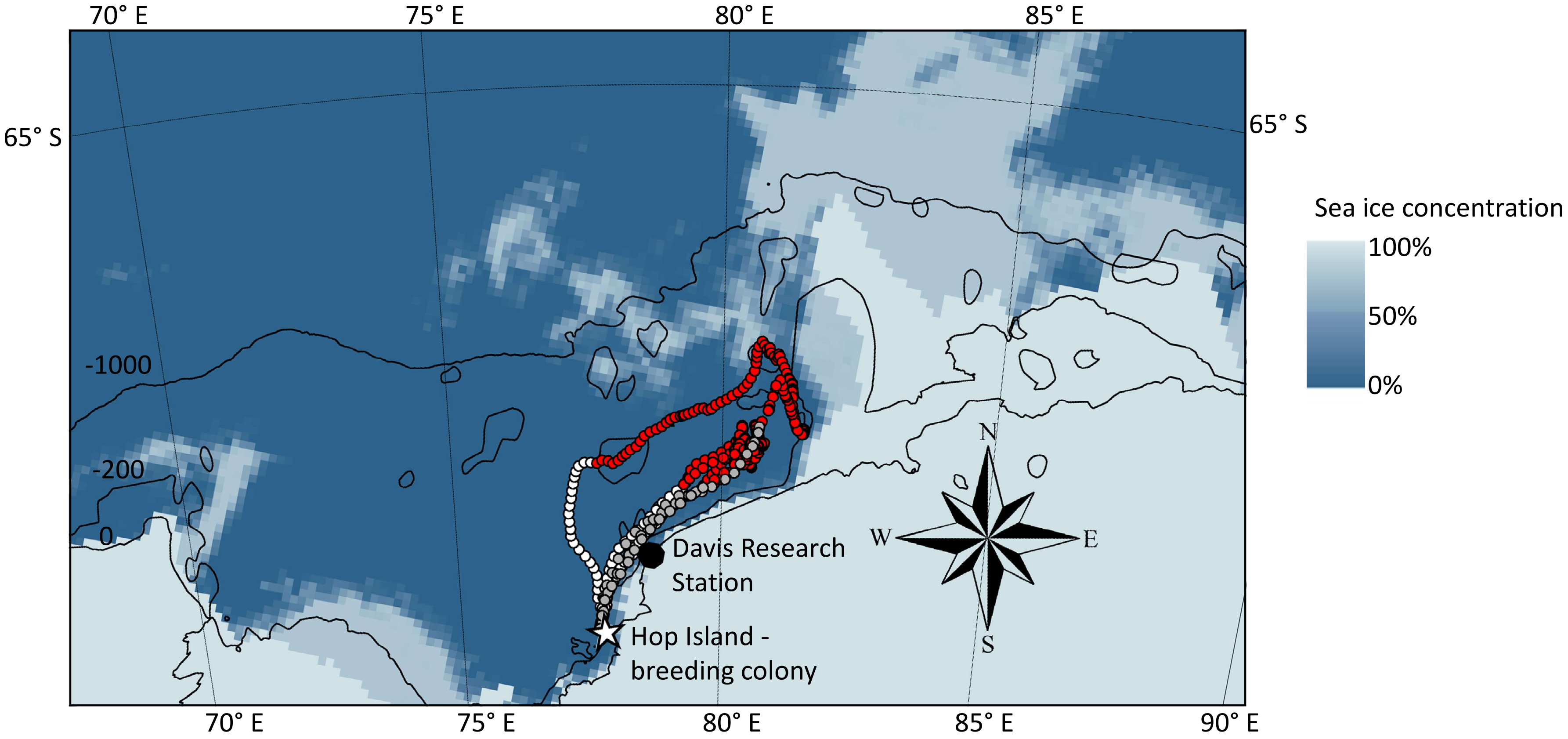

Antarctic Petrel 09

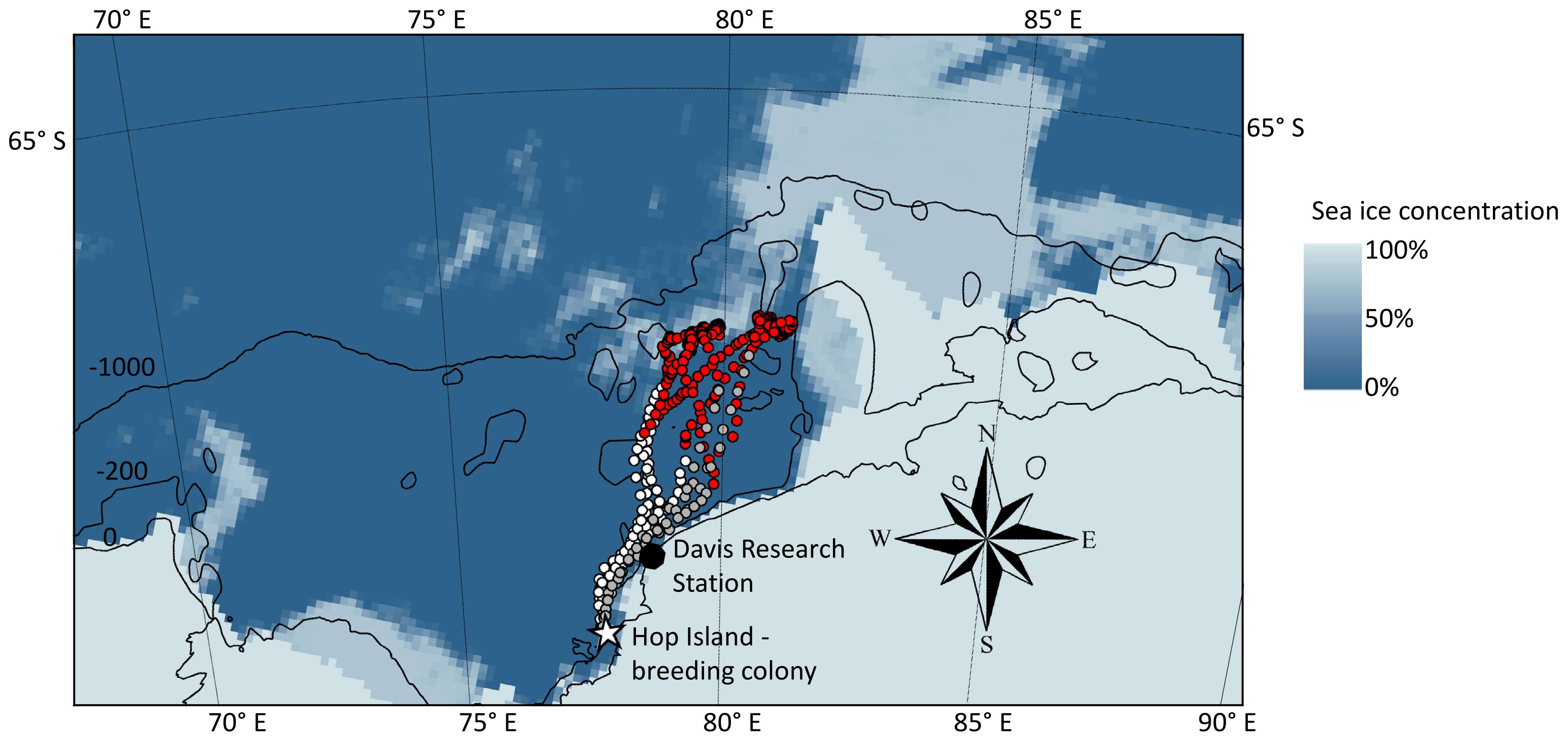

Antarctic Petrel 16

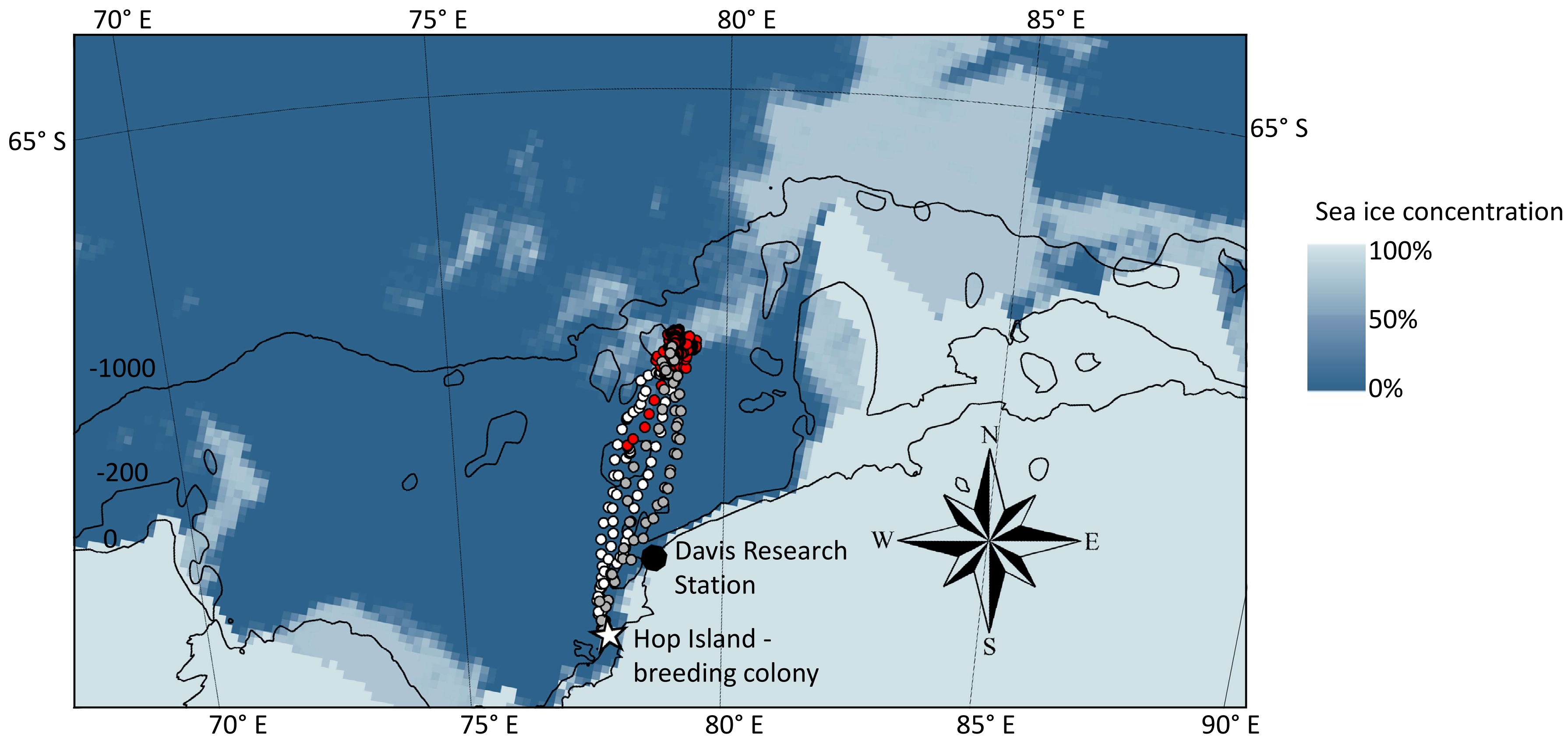

Antarctic Petrel 17

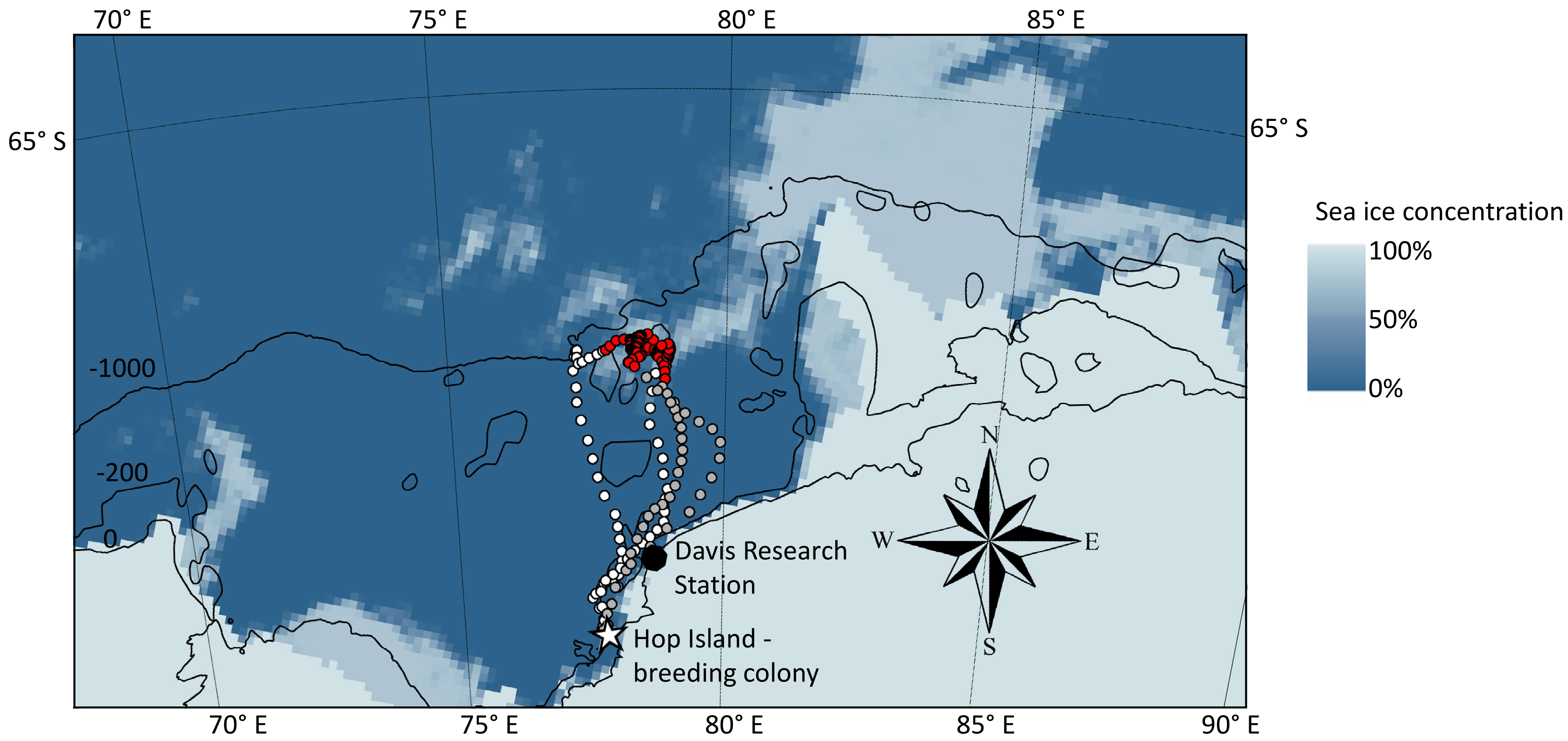

Antarctic Petrel 18

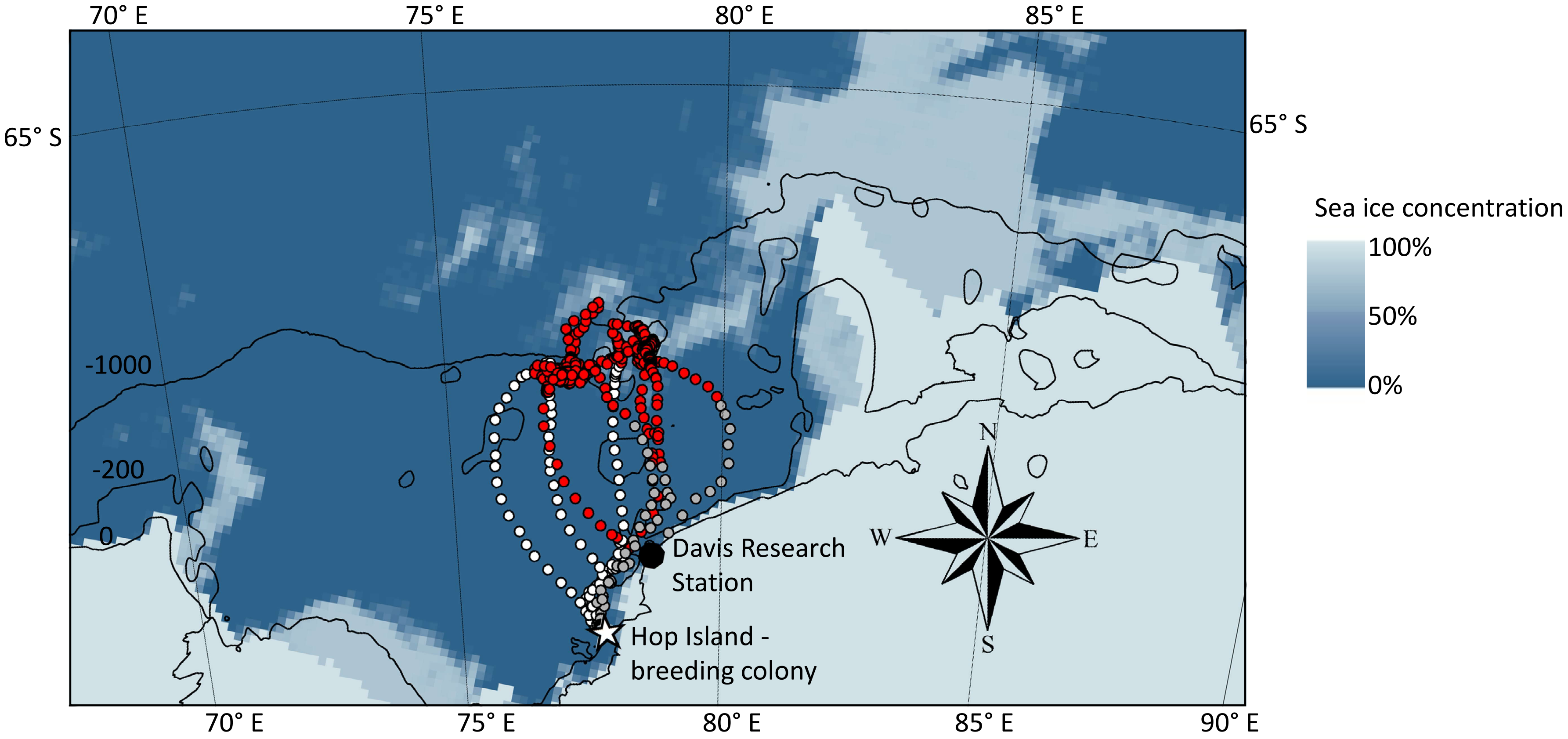

Antarctic Petrel 19

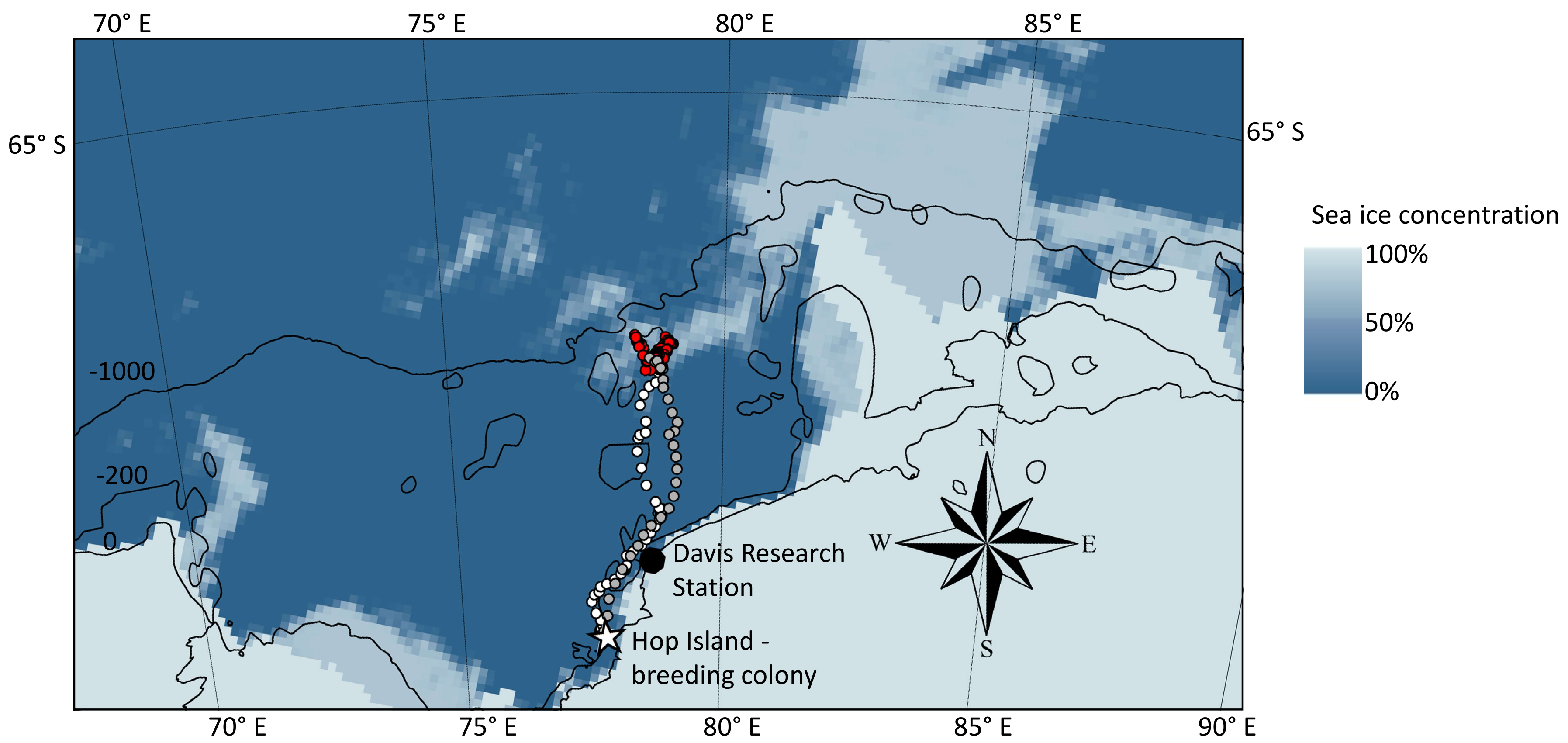

Antarctic Petrel 20

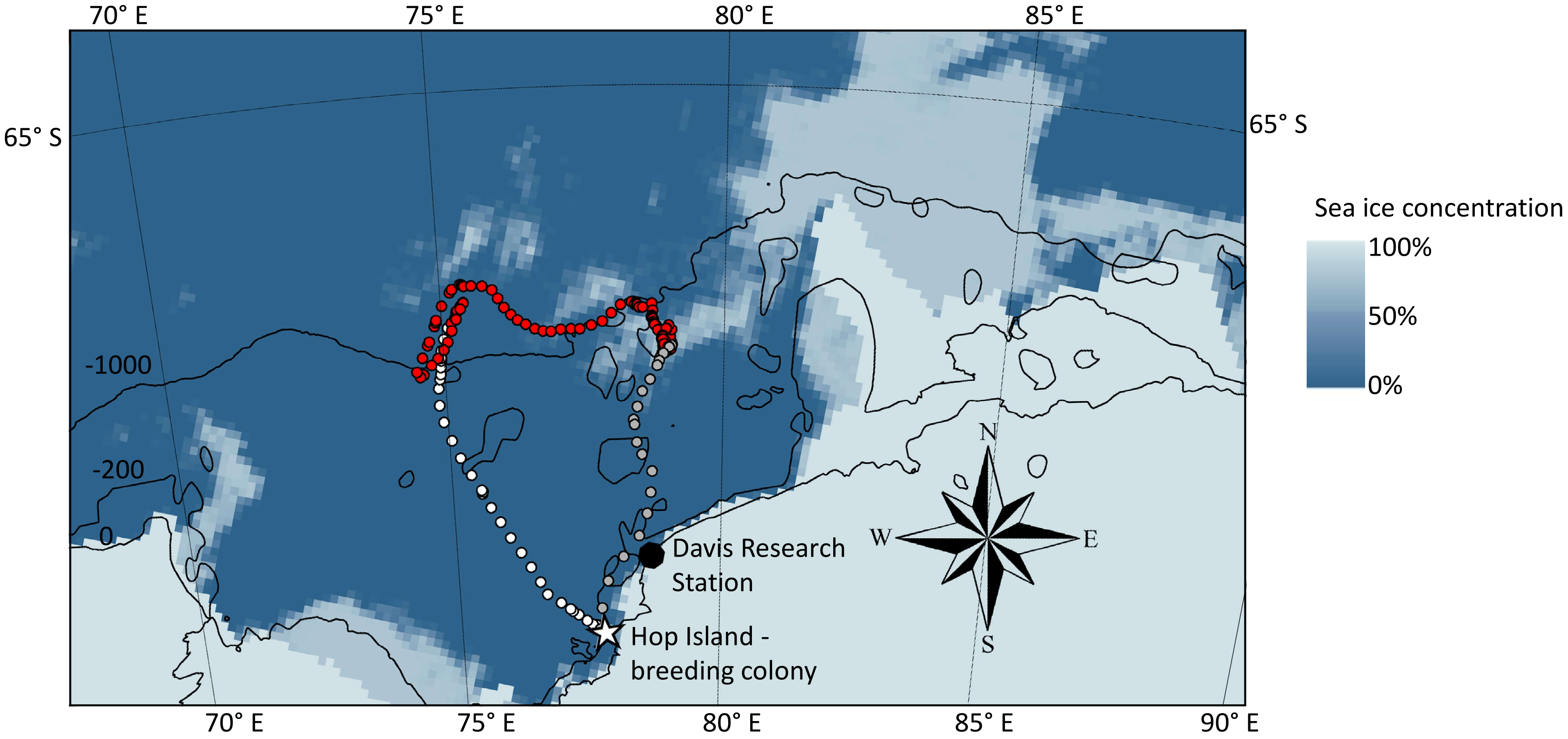

Antarctic Petrel 26

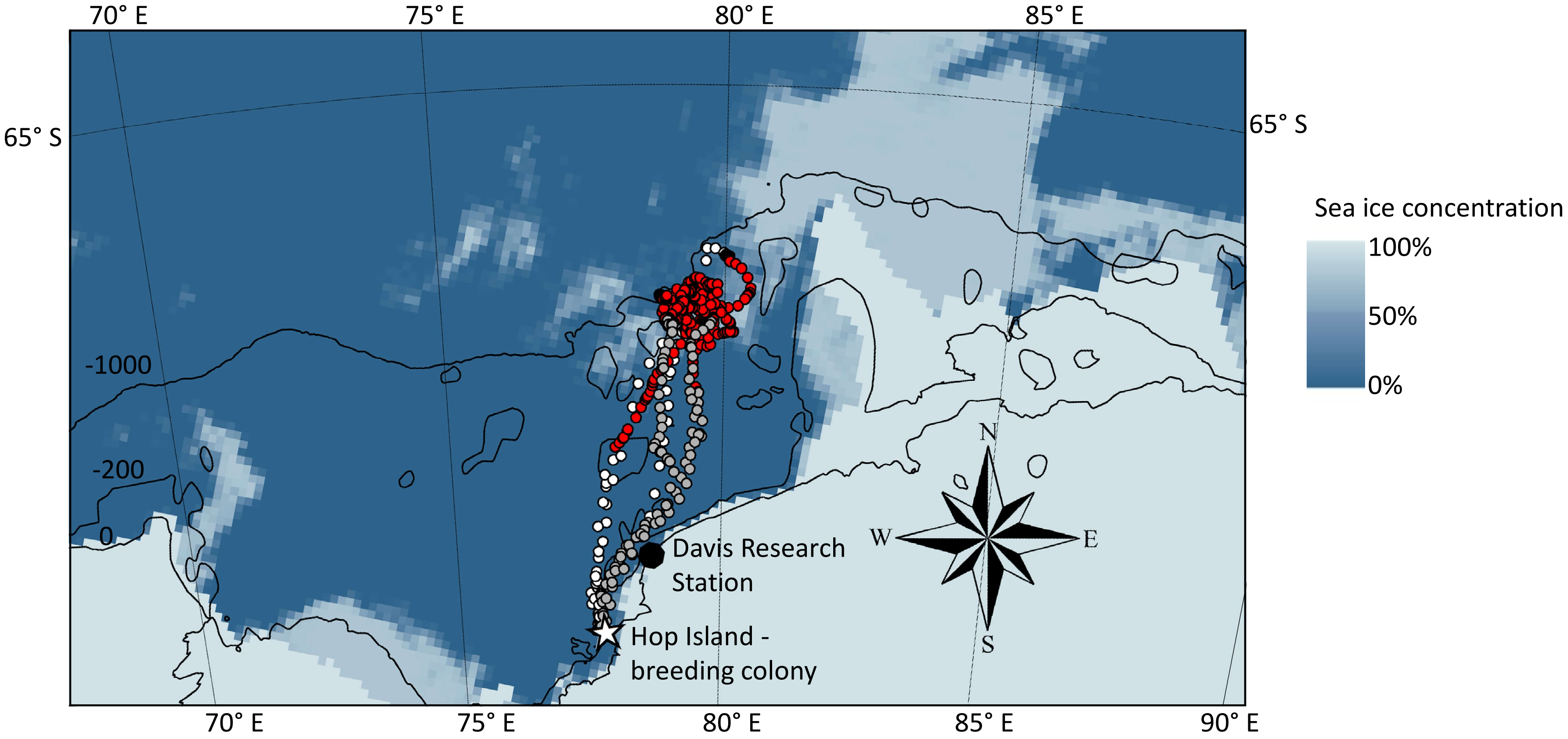

Antarctic Petrel 30

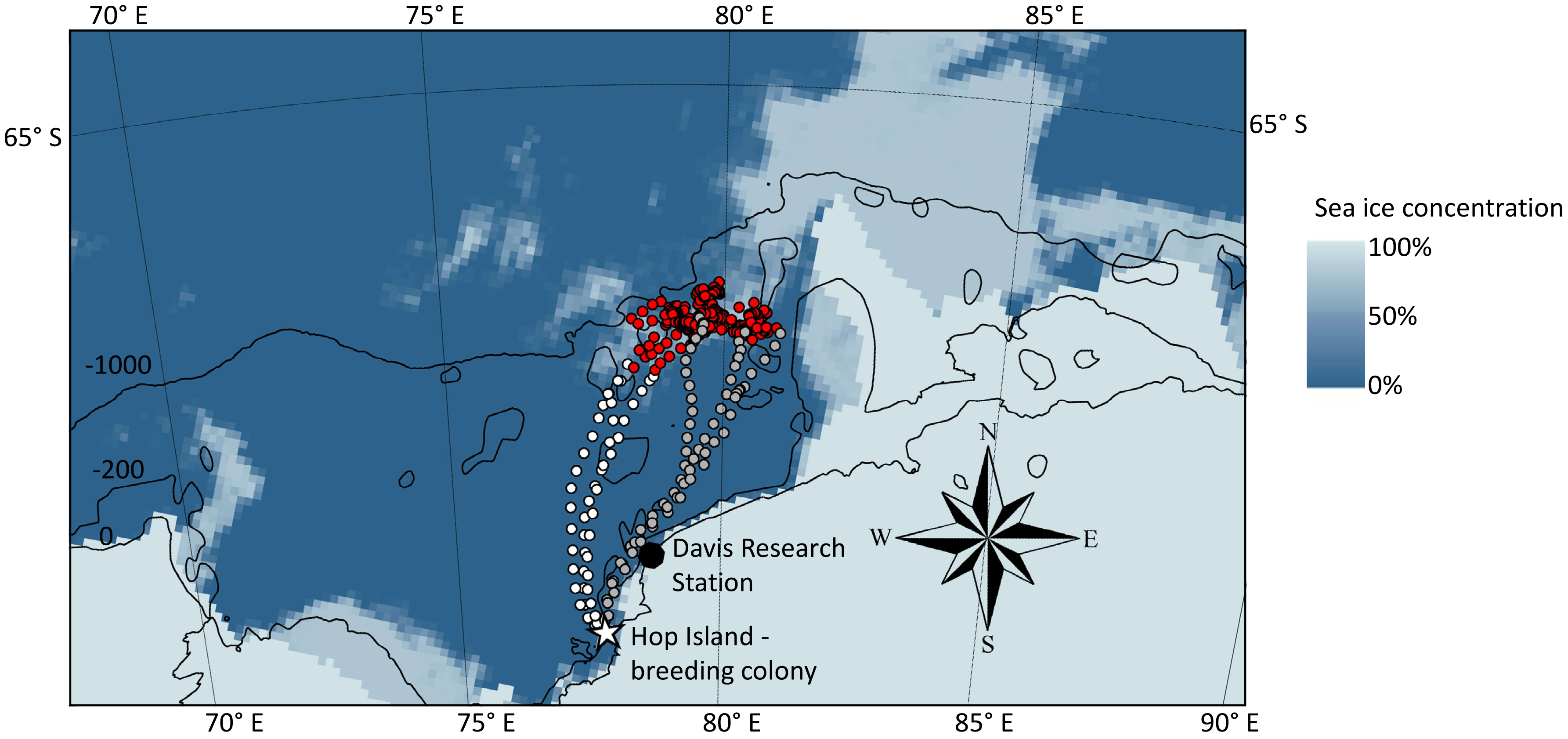

Southern Fulmar 07

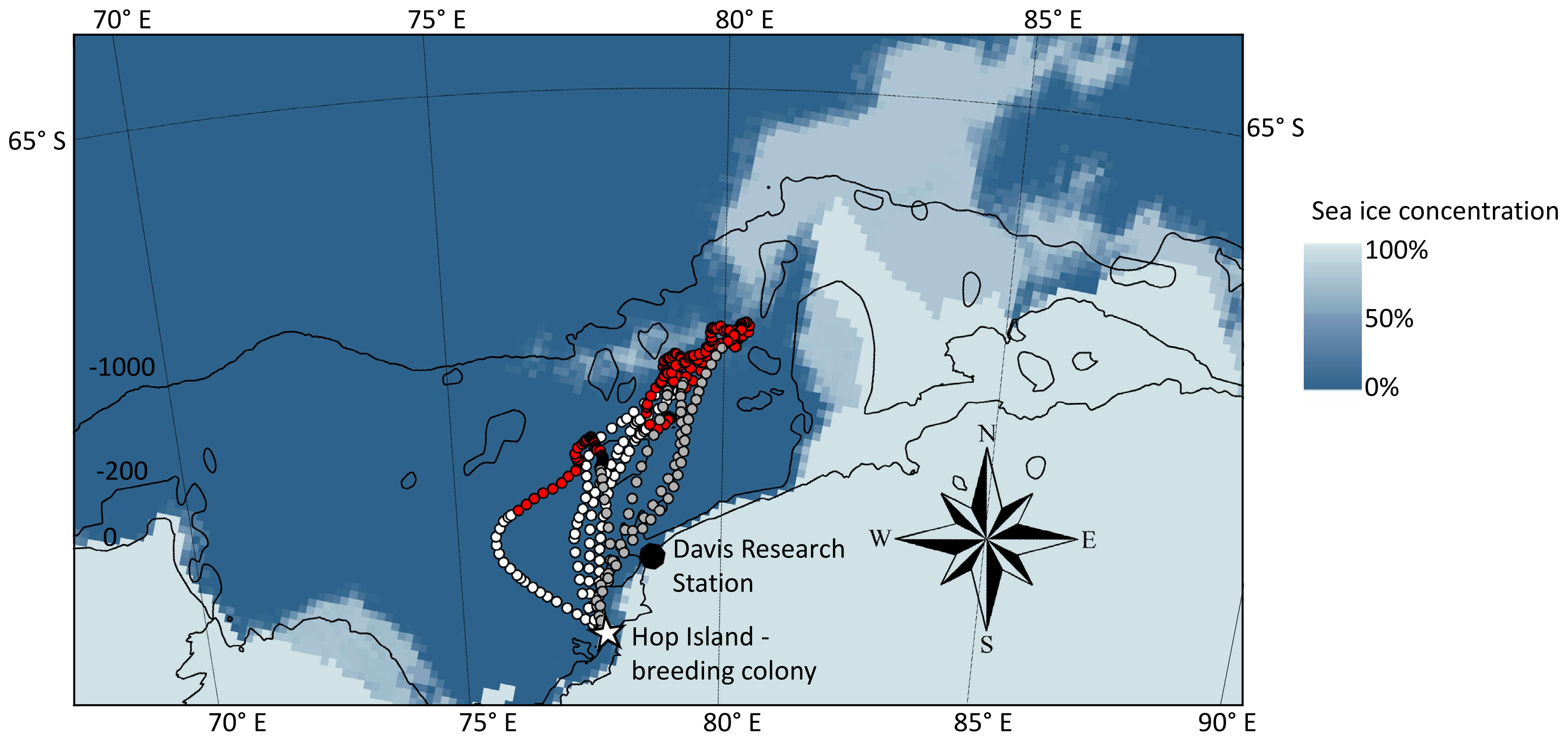

Southern Fulmar 17

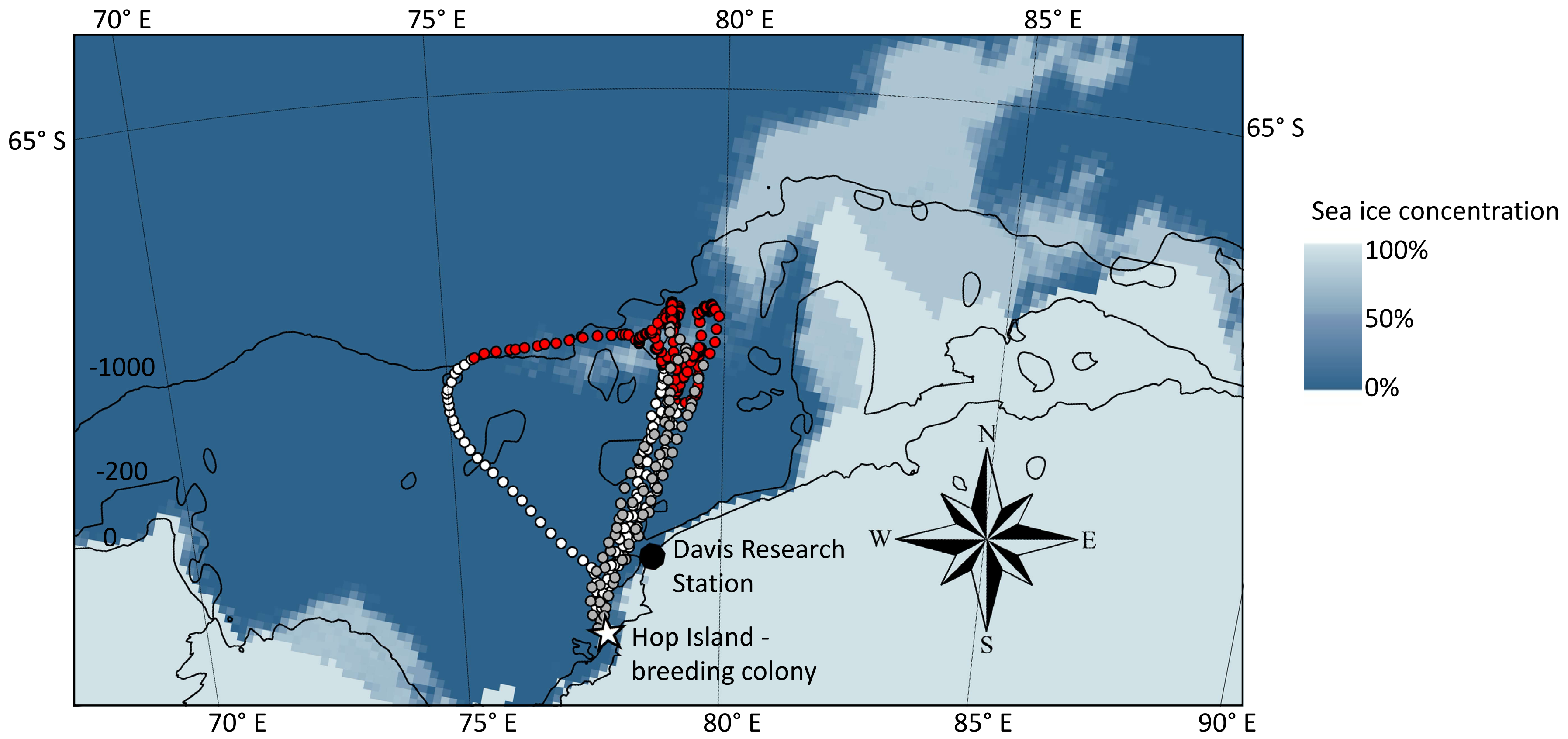

Southern Fulmar 18

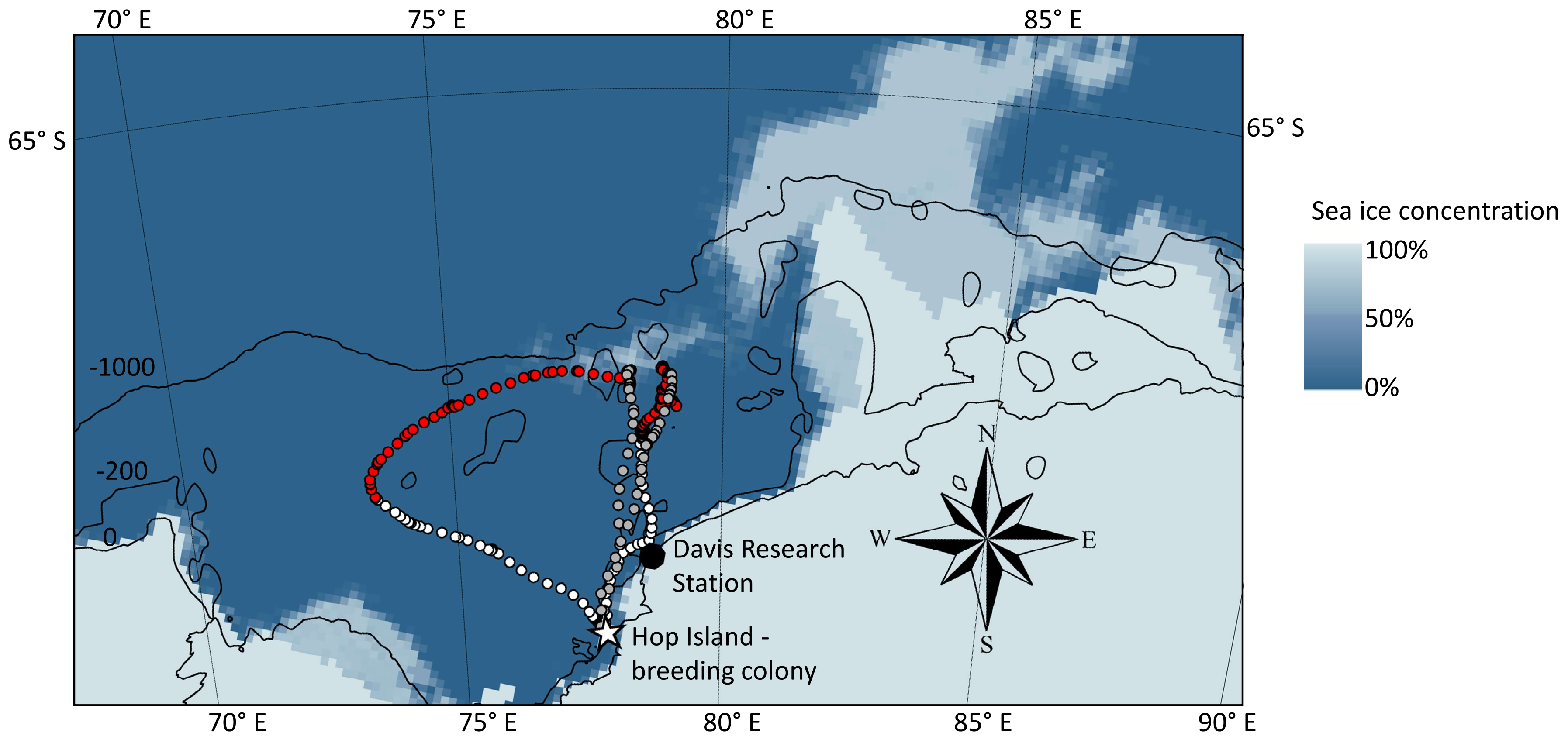

Southern Fulmar 19

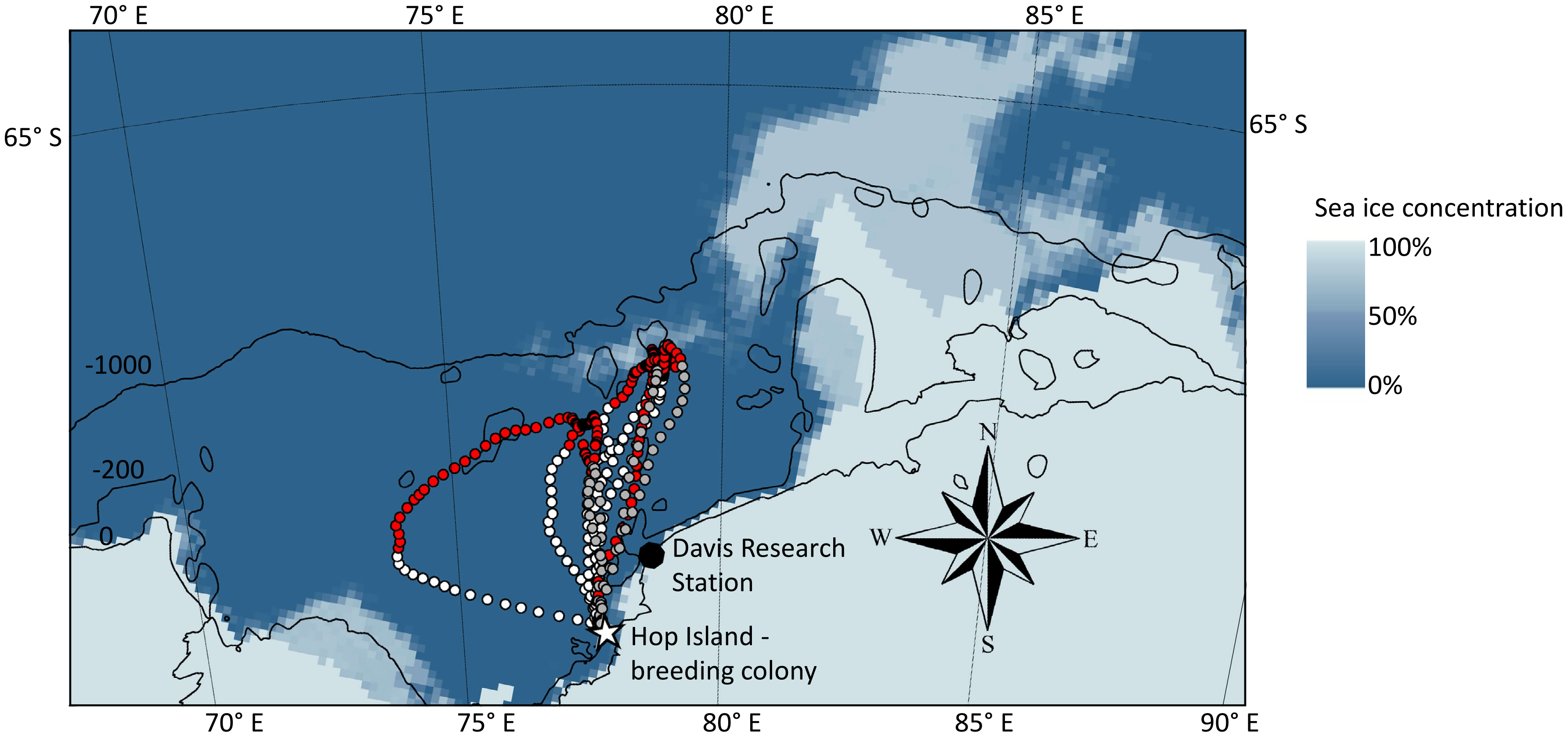

Southern Fulmar 20

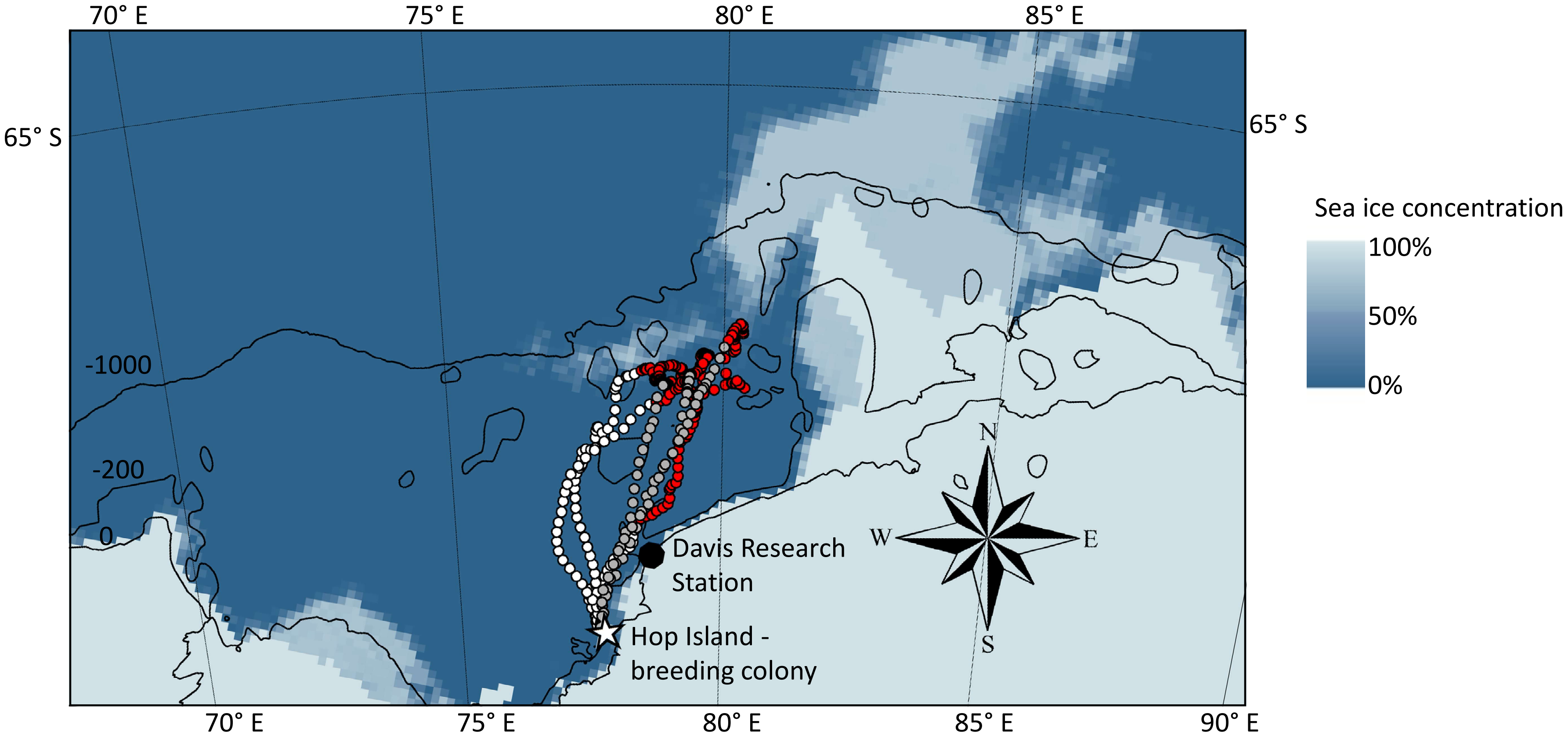

Southern Fulmar 21

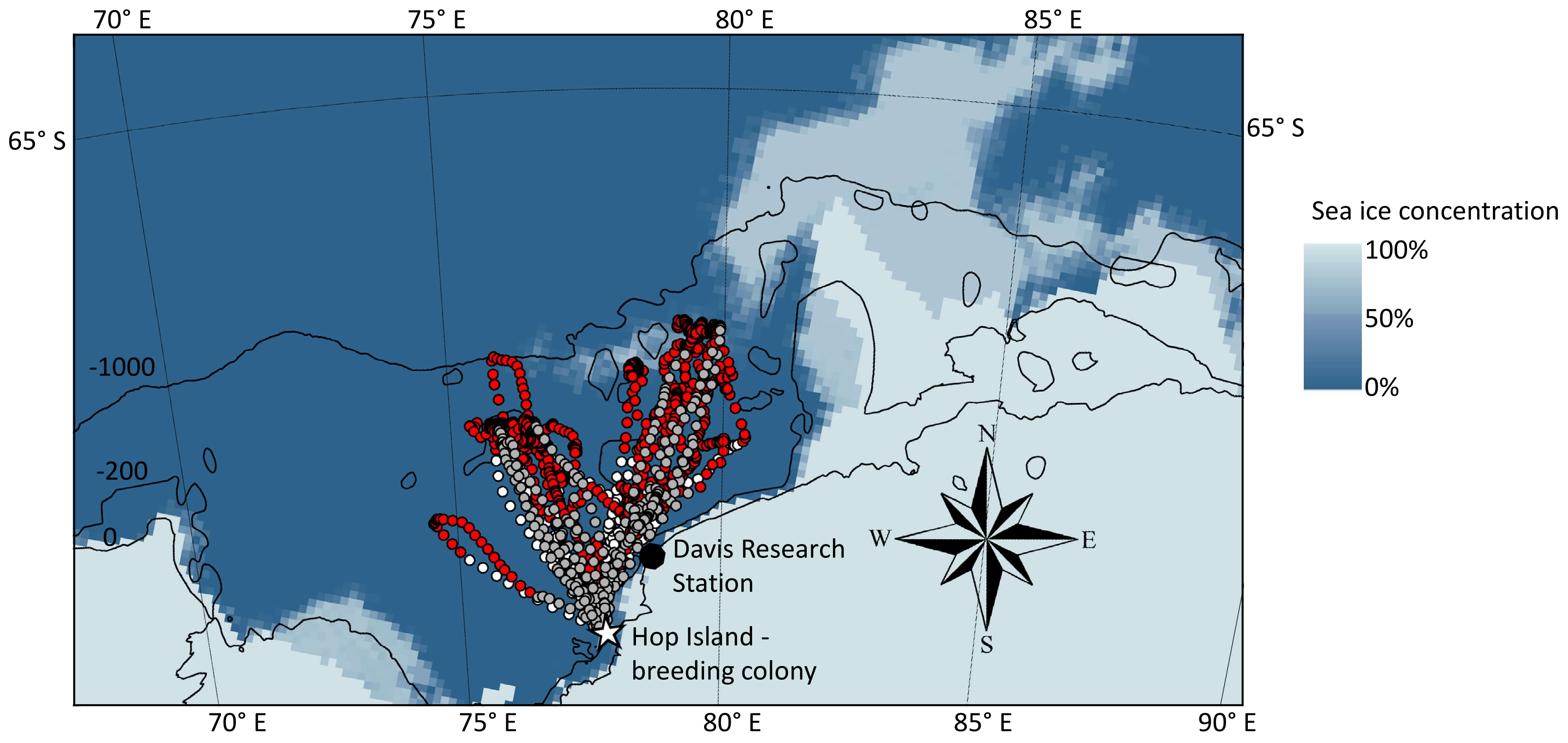

Southern Fulmar 22

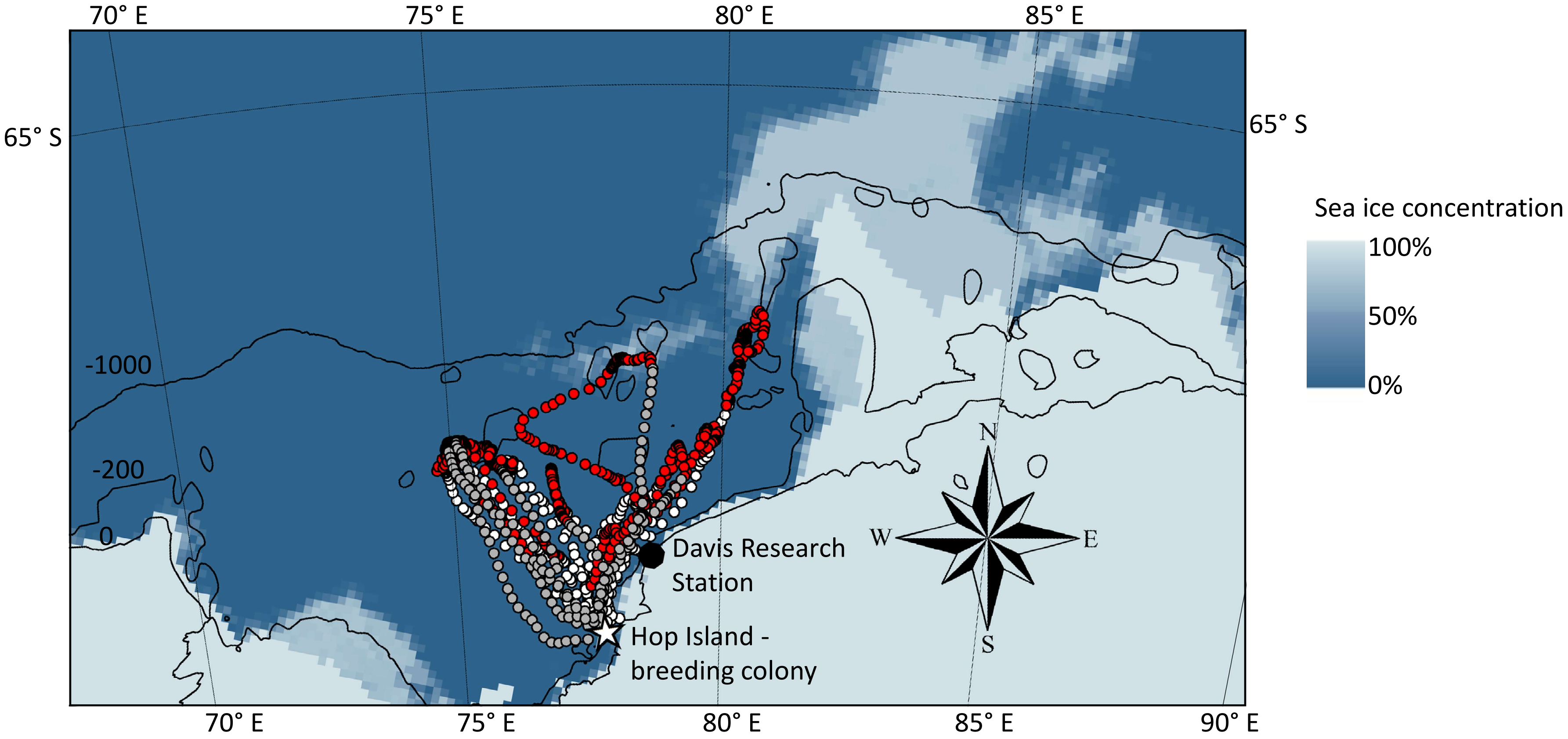

Southern Fulmar 23

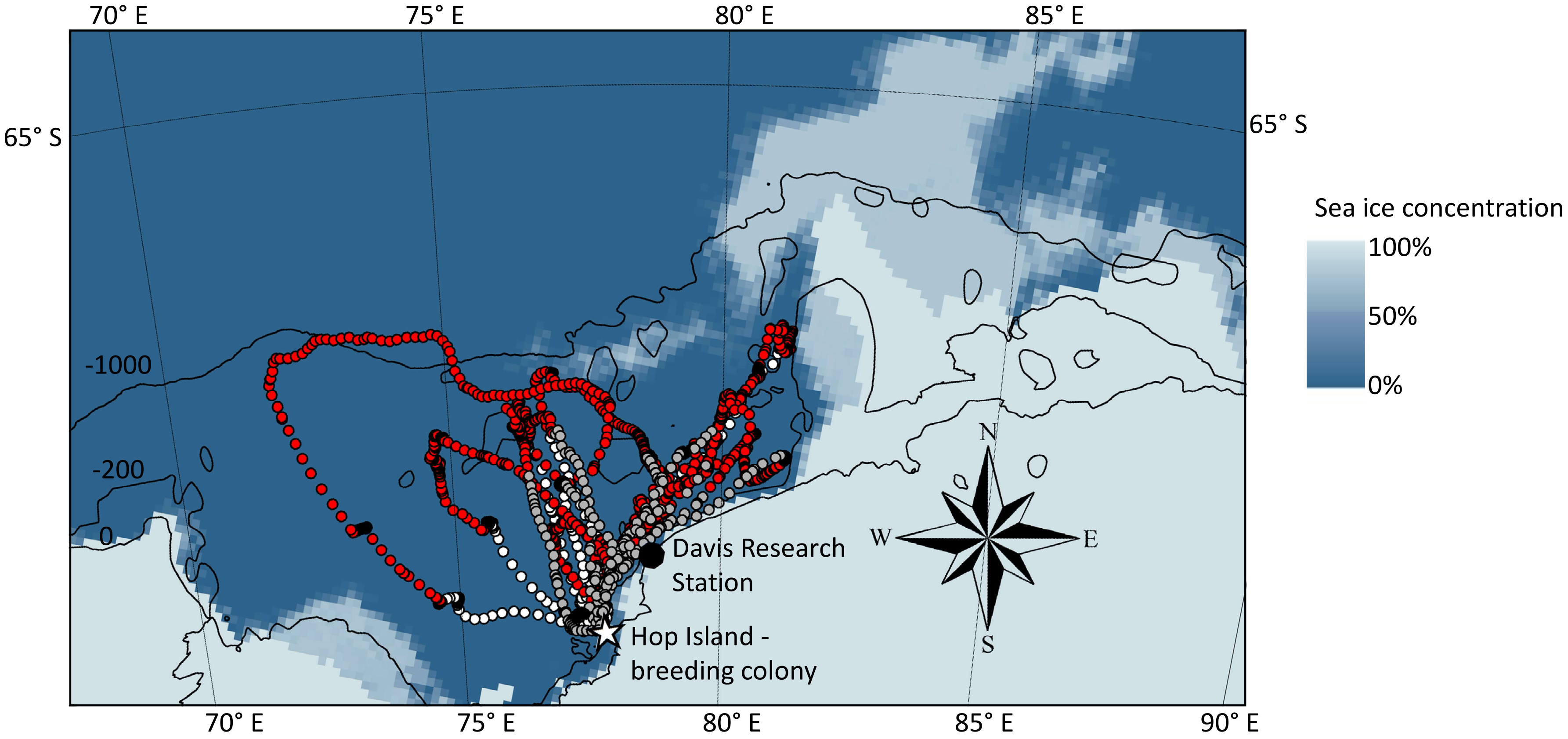

Southern Fulmar 24

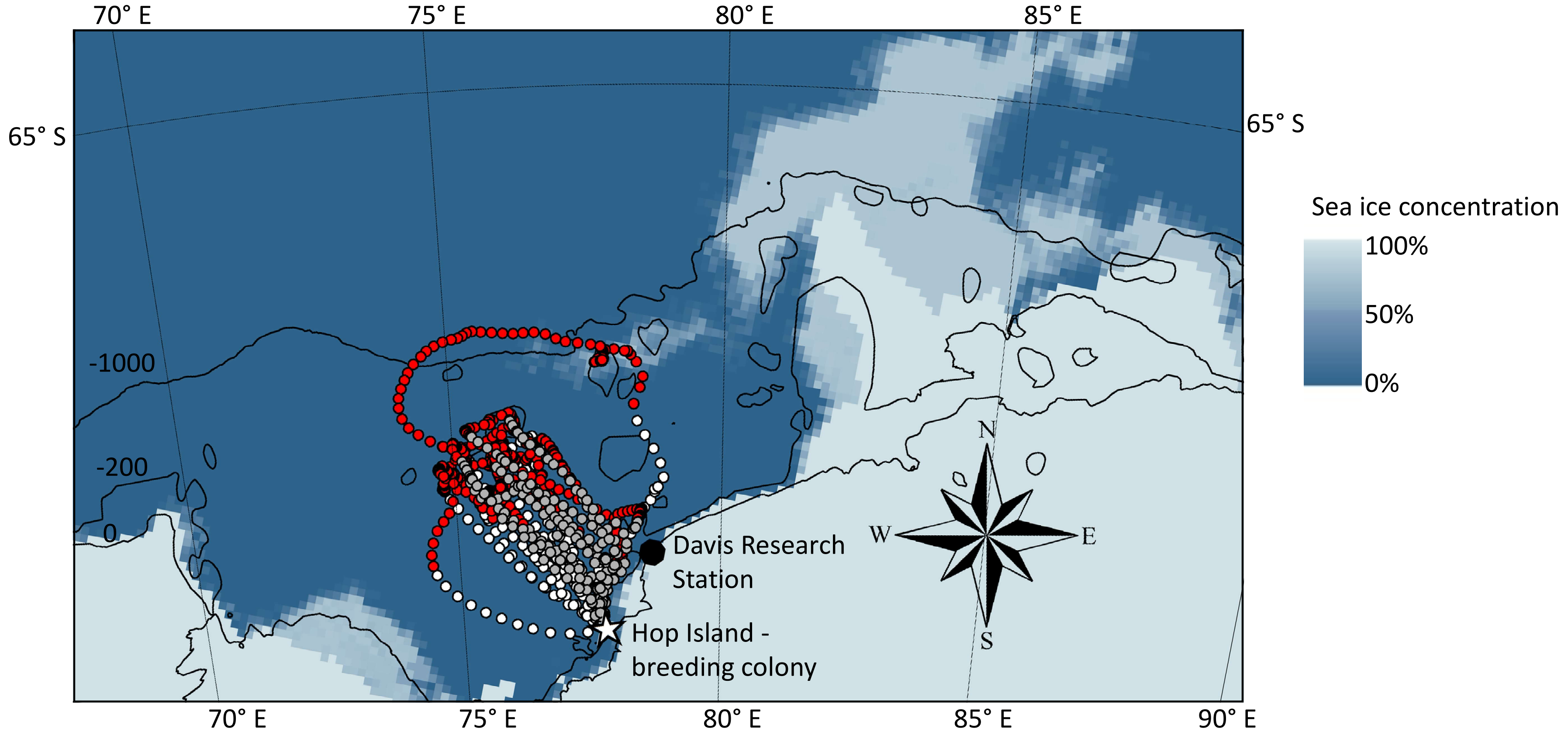

Southern Fulmar 25

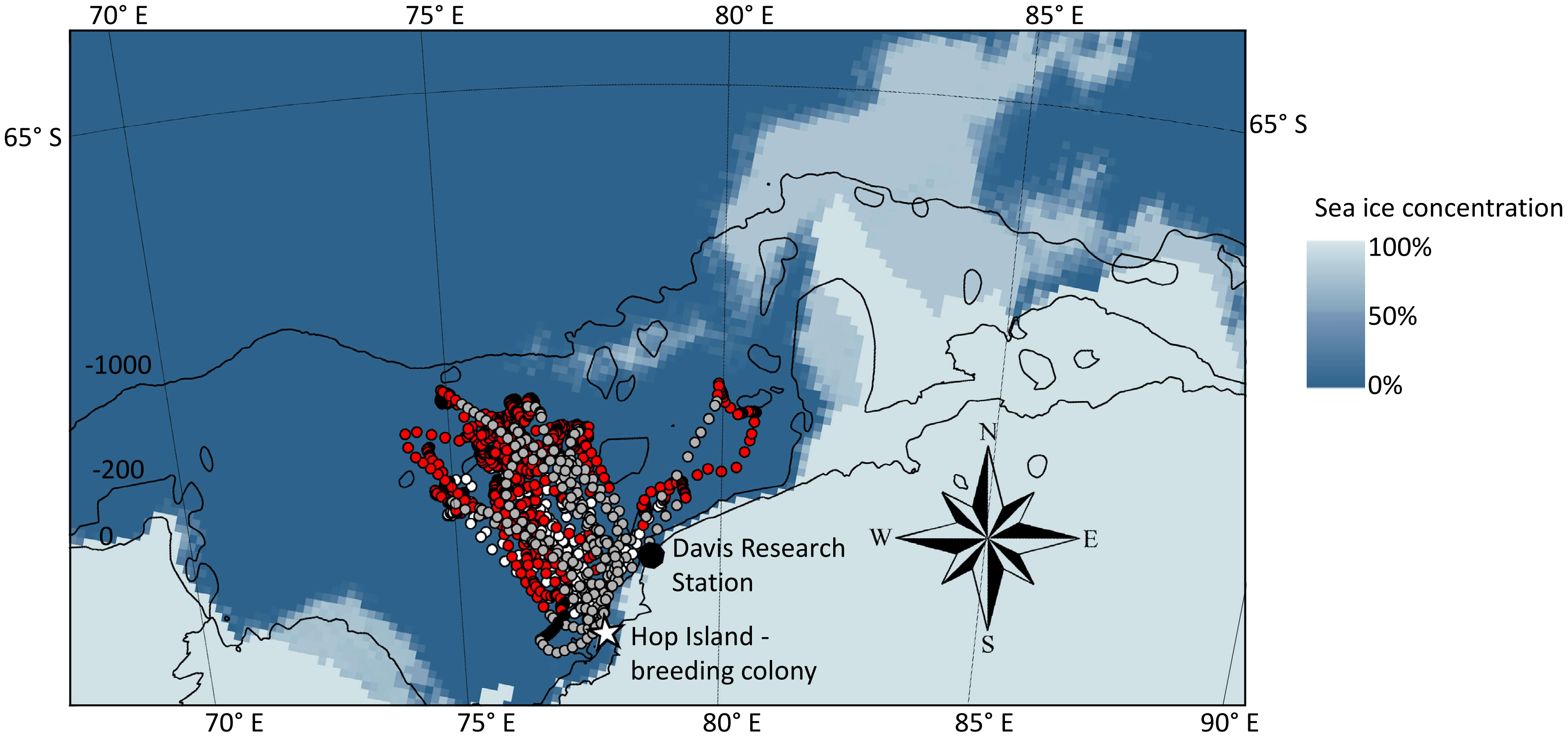

Supplement: Supplementary file 2 — Appendix S2 [file ECE3-11-4972-s001.pdf]
